# Isopentane Disproportionation in Lewis Acidic Chloroaluminate Ionic Liquid

**DOI:** 10.1021/jacs.6c03060

**Published:** 2026-06-17

**Authors:** Jiande Mai, David E. Ryan, Wei Zhang, Benjamin A. Jackson, Kiyoung Jo, Janos Szanyi, Oliver Y. Gutiérrez, Honghong Shi, Donald M. Camaioni, Rachit Khare, Mal-Soon Lee, Huamin Wang, Sungmin Kim, Johannes A. Lercher

**Affiliations:** 1 Institute for Integrated Catalysis, 6865 Pacific Northwest National Laboratory, Richland, Washington 99354, United States; 2 Physical and Computational Sciences Directorate, Pacific Northwest National Laboratory, Richland, Washington 99354, United States; 3 Department of Chemistry and Catalysis Research Center, TU München, Garching 85748, Germany

## Abstract

Lewis acidic chloroaluminate ionic liquid diluted in dichloromethane catalyzes the disproportionation of alkanes, such as isopentane, via carbenium ions in a reaction readily initiated by carbenium ion precursors such as *tert*-butyl chloride (TBC). The carbenium ion-AlCl_4_^–^ ion-pairs stabilized by the ionic liquid-dichloromethane solution are the key intermediates in two distinctive kinetic regimes, i.e., a transient regime (0–5 min) and a steady-state regime (after 5 min). The transient regime constitutes the majority of isopentane conversion and is governed by the initial carbenium ion concentration. In the steady-state regime, disproportionation occurs at a considerably lower rate, affected by the carbenium ion concentration, the concentration of the ionic liquid, and the reaction temperature. The formation of alkenes observed in the ^1^H NMR spectra of the reacting substrates, along with the DFT calculations, suggests that deprotonation of carbenium ion-pairs reduces their concentration, decreasing, in turn, the reaction rate. Kinetic modeling indicates that the transient regime is significantly controlled by the hydride transfer (*k*_HT_) and the deprotonation rate constants (*k*_DP_), while the steady-state regime is additionally influenced by the alkene protonation rate constant (*k*_–DP_). The overall activation energy of the reaction at the steady state, expressed as *E*_a,steady‑state regime_ = *E*_a,HT_ – *E*_a,DP_ + *E*_a,–DP_, was 54 kJ/mol. The reaction mechanism and the kinetics highlight the potential of Lewis acid-catalyzed conversions of hydrocarbons under remarkably mild conditions.

## Graphical abstract

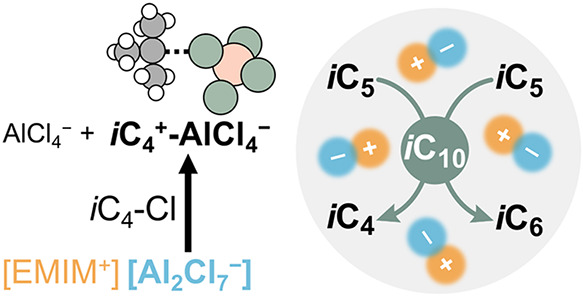

## 1 Introduction

Acid-catalyzed hydrocarbon conversions are fundamental to the petroleum and chemical industries.^1–4^ Carbenium ion-based mechanisms dominate in these conversions. Both Brønsted and Lewis acids are able to generate and stabilize carbenium ions, typically via protonation of alkenes by Brønsted acids or via hydride abstraction or hydride transfer catalyzed by Lewis acids.^5–7^ The carbenium ions have been shown to be transition states and intermediates in hydrocarbon transformations such as catalytic cracking,^8,9^ paraffin isomerization,^10,11^ olefin oligomerization,^12–14^ and short-chain alkane alkylation.^15,16^ While highly efficient, Lewis acid catalysts such as AlCl_3_ pose challenges in handling and recycling, byproduct formation, and equipment corrosion.^12,17^ Thus, easier manageable Lewis acid catalysts and robust catalytic processes retaining high efficiency and selectivity would be of high interest.

Aluminum chloride-based Lewis acidic ionic liquids are recognized as promising, “greener” alternatives to conventional acid catalysts, offering low toxicity, minimal diffusion limitations, good recyclability, and easily tunable physical and chemical properties.^18^ Moreover, their ability to stabilize carbenium ions and create a highly ionic reaction environment has enabled a wide range of carbenium ion-mediated alkane reactions, such as isomerization,^19–21^ alkylation^22–26^ and cracking.^27–29^ A tandem cracking-alkylation approach using a chloroaluminate ionic liquid (*N*-butylpyridinium chloride and AlCl_3_) that converts waste polyolefins into gasoline-range branched alkanes has been recently reported.^30,131^ Coupling the endothermic cleavage of the polymer C–C bonds with the exothermic alkylation of alkenes formed in this process enabled the complete conversion of LDPE below 100 °C. Both cracking and alkylation proceed via carbenium ions, generated by Lewis acidic anion species in chloroaluminate ionic liquid.^30,131^ The formation of carbenium ions is typically initiated by chloride abstraction from an initiator, *tert*-butyl chloride (TBC). Subsequently, the *tert*-butyl carbenium ions formed in this step abstract hydrides from the polymer chains or the alkylating agent, i.e., isopentane or isobutane.^30,131^ Cracking of the carbenium ions in the polymer strands leads to alkenes that, in turn, react with the *tert*-butyl or pentyl carbenium ions. This C–C coupling step results in the formation of a highly branched, higher-molecular-weight carbenium ion, which is subsequently converted into an alkane by hydride abstraction from the alkylating agent.

However, in the presence of aluminum halides (or aluminum halide ionic liquids) and carbenium ion initiators, isoalkanes used as alkylating reagents can also undergo conversion themselves. For example, Schneider et al. reported isopentane disproportionation induced by *tert*-butyl fluoride and boron trifluoride, producing methylpentanes and isobutane.^31^ An activity comparison showed that supported chloroaluminate ionic liquids and immobilized AlCl_3_ on silica outperformed zeolites and sulfated zirconias for the disproportionation of light paraffins.^32^ Notably, this disproportionation is commonly observed in a variety of carbenium ion-mediated alkane reactions, especially Lewis acid-catalyzed alkane isomerization^19,33–36^ and alkylation.^37–39^ Besides, the alkane disproportionation has been reported in the context of Brønsted acid-catalyzed reactions involving carbenium ions.^40–42^ Carbenium ions are highly reactive and readily undergo rearrangement and intermolecular interactions, resulting in complex reaction pathways and a broad product distribution. Understanding the reaction mechanisms, controlling the reactivity of carbenium ions, and manipulating their reaction pathways are therefore crucial for enhancing the efficiency and selectivity of the hydrocarbon conversion processes.

To fundamentally understand these Lewis acid-catalyzed conversions, particularly the reactions with iso-alkanes initiated by TBC, we studied isopentane disproportionation by combining reaction kinetics with mechanistic investigations. The latter includes the characterization of active species by *in situ* NMR analysis and DFT calculations. Our study provides a basis for improved control of the efficiency and selectivity of carbenium ion-mediated hydrocarbon conversion processes.

## 2 Results and Discussion

### 2.1 Isopentane Disproportionation in the Polyethylene Conversion

When converting isopentane together with low density polyethylene (LDPE) in a well stirred glass tube reactor, changing the amount of TBC, i.e., the carbenium ion initiator, from 0.054 to 0.22 mmol, led to a marked production of mostly C_4_ and C_6_, before significant conversion of LDPE occurred (Figure 1). Note that the ionic liquid used throughout this work is diluted in dichloromethane (see Supporting Information for details). In agreement with earlier findings, using 0.054 mmol of TBC yielded alkane products in the C_4_–C_13_ range (excluding *i*C_5_) equivalent to approximately twice the mass of LDPE converted (Figure 1A, green-shaded area).^30^ Intriguingly, when using 4-fold higher amount TBC (0.22 mmol), the relation between alkane product yield and LDPE conversion showed the identical dependence, but the intercept with the *Y*-axis indicated an alkane product yield of 120 wt % (Figure 1A, orange dots), i.e., additional 240 mg of the isopentane were converted. The reaction showed high selectivity toward C_4_ and C_6_ alkane products, indicating a bimolecular reaction between two isopentane molecules.

**Figure 1:**
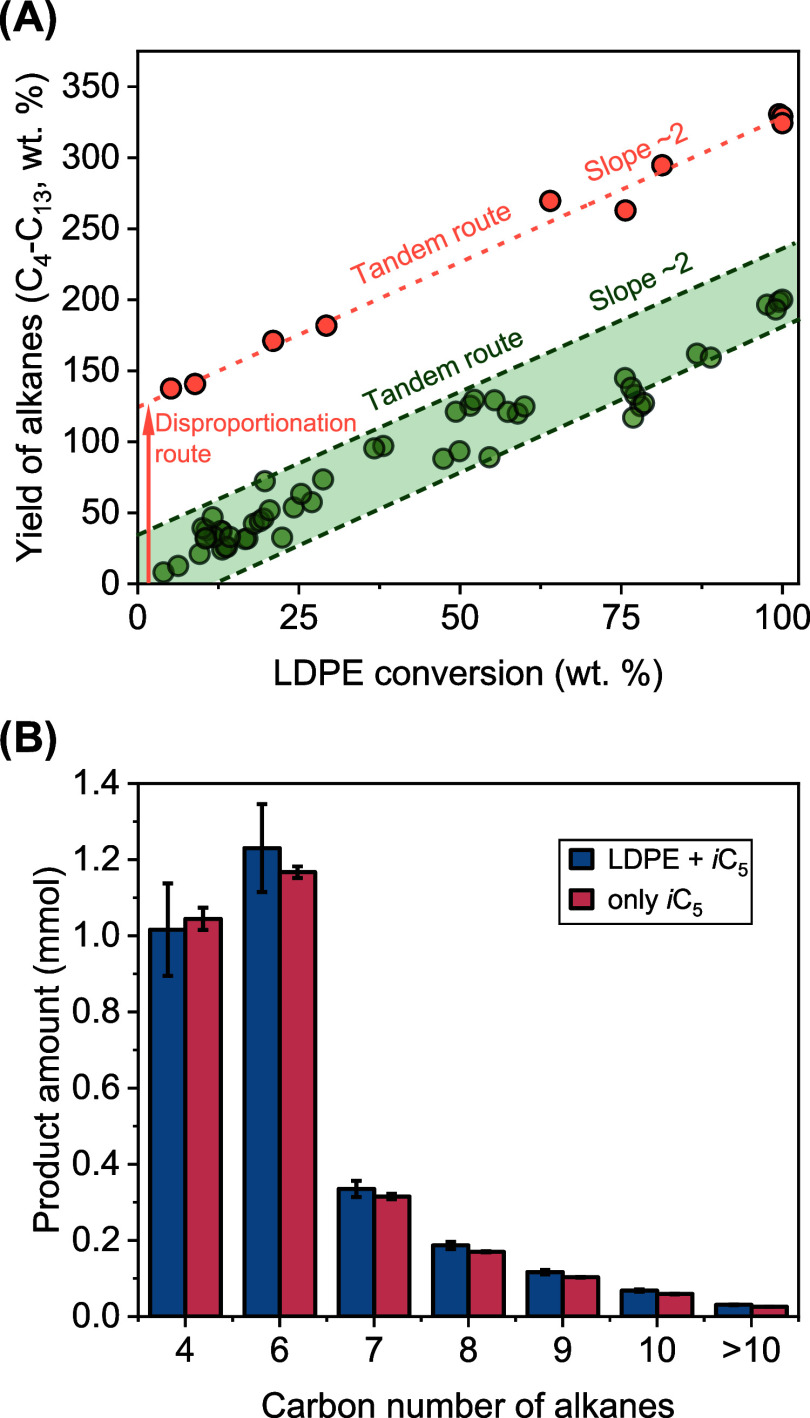
Comparison of LDPE tandem cracking-alkylation and isopentane disproportionation reaction. (A) The yield of alkanes (C_4_–C_13_, excluding *i*C_5_) plotted against LDPE conversion. Reaction conditions for the orange dots were as follows: LDPE, 200 mg; *i*C_5_, 11.1 mmol (800 mg); ionic liquid, 3 mmol; TBC, 0.22 mmol (20 mg); dichloromethane, 3 mL; and temperature, 70 °C. The green-shaded area represents a general linear fit to data obtained under similar conditions using 0.054 mmol (5 mg) TBC over a temperature range of 30–70 °C. The data in the green-shaded area are adapted with permission from ref 30. Copyright 2026, AAAS. (B) Alkane product distributions (at 5 min reaction time) from LDPE tandem cracking-alkylation (blue) and isopentane disproportionation reaction (red). Reaction conditions were as follows: LDPE, 200 mg (blue) or 0 mg (red); *i*C_5_, 11.1 mmol (800 mg); ionic liquid, 3 mmol; TBC, 0.22 mmol (20 mg); dichloromethane, 3 mL; and temperature, 70 °C.

To test this conclusion, we performed identical experiments using isopentane with and without LDPE. To examine the initial stage of the reaction, we collected the products after 5 min, a point at which the LDPE conversion was negligible. For both experiments, the product yields were approximately 240 mg (3.3 mmol isopentane converted). The product distributions were almost identical, with 75 mol % of the products corresponding to nearly equimolar amounts of C_4_ and C_6_ alkanes (Figure 1B). The results indicate that the initial product increase (∼ 5 min) during tandem cracking and alkylation with LDPE is caused by fast isopentane disproportionation; the products exceeded the concentrations of TBC by at least an order of magnitude, i.e., each TBC molecule initiated at least 10 turnovers. The identical slopes of 2 in the yield vs conversion curve for experiments with 0.054 and 0.22 mmol TBC strongly suggest that the disproportionation mainly occurs in the very initial stages of the reaction. Subsequently, a larger relative fraction of carbenium ions must reside on the polymer strands, and the normal LDPE tandem cracking-alkylation dominates.

### 2.2 Transient and Steady-State Regimes of Isopentane Disproportionation

Isopentane was converted with a high rate only during a short time span, approximately in the first 5 min. Afterward, it was converted with a rate that was approximately an order of magnitude slower (Figure 2A). Thus, it is helpful to partition the time-resolved profiles (Figure S1A) into two regimes: the initial “transient regime” (0–5 min) and the subsequent slow “steady-state regime” (5–60 min). This marked change in conversion rate is consistent with the observation in Figure 1A, where the isopentane disproportionation only occurs during the initial stage of the reaction.

**Figure 2:**
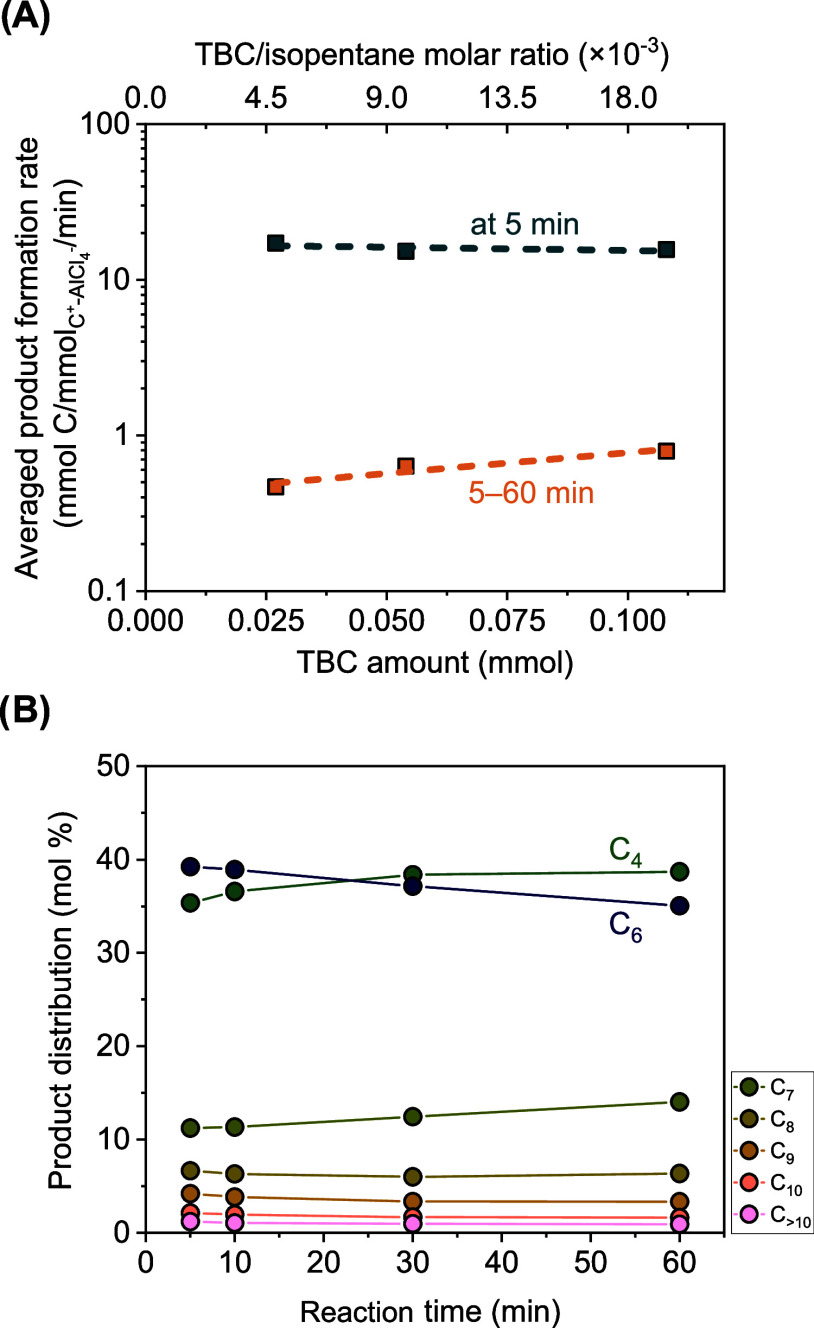
The rate difference and the selectivity consistency between the transient and steady-state regimes of isopentane disproportionation. (A) The averaged product formation rates for 0–5 min (teal) and 5–60 min (orange) plotted against the TBC amount. See Procedure for liquid phase product analysis of isopentane disproportionation in Supporting Information. The top axis displays the corresponding molar ratio of TBC to isopentane. Reaction conditions: *i*C_5_, 5.5 mmol (400 mg); ionic liquid, 1 mmol; TBC, 0.027 mmol (2.5 mg), 0.054 mmol (5 mg), or 0.11 mmol (10 mg); dichloromethane, 3 mL; and temperature, 25 °C. (B) Product distribution of isopentane disproportionation along the reaction time. No methane, ethane, or propane was detected. Reaction conditions: *i*C_5_, 5.5 mmol (400 mg); ionic liquid, 1 mmol; TBC, 0.11 mmol (10 mg); dichloromethane, 3 mL; and temperature, 25 °C.

To investigate the underlying disproportionation mechanism, we next assess the impact of different reaction conditions on each regime independently. In the transient regime (0–5 min), the isopentane conversion at 5 min exhibited a linear correlation with the TBC concentration (Figure S1B). Notably, in the absence of TBC, isopentane conversion was negligible, i.e., approximately 1% after 2 h, consistent with previous findings.^30^ Aside from the TBC concentration, other reaction parameters, including the amount of ionic liquid, isopentane, and temperature, had little to no impact on the isopentane conversion in the transient regime (see Figure S2).

We hypothesize that the empirical relationship between the added TBC and the system’s kinetic behavior in the transient regime does not reflect a direct role of TBC. Instead, the underlying determining factor of the isopentane conversion in this regime is the initial carbenium ion concentration, generated by the reaction between the ionic liquid and TBC (Figure S2A).^43^ This hypothesis is substantiated by the fact that the isopentane conversion achieved in the transient regime was always determined by the limiting stoichiometry of either the ionic liquid or TBC. Since TBC was in general the limiting reagent in all experiments, we hypothesize that most of the TBC is converted to the carbenium ions or the corresponding ion-pairs. Therefore, isopentane conversion at 5 min may be inferred to correlate directly with the amount of carbenium ions produced in the reactor. Extrapolating the isopentane conversion to 0.22 mmol of TBC predicts a 5 min conversion of 60% (240 mg) at room temperature (Figure S1B). Additionally, since temperature does not significantly affect isopentane conversion in the transient regime (Figure S2C), the prediction remains valid at 70 °C. This predicted value agrees well with the value we observed in Figure 1A.

In the following steady-state regime (>5 min), the product amount increased approximately linearly with reaction time. In contrast to the transient regime, the product formation rate increased with higher ionic liquid amount (Figure S3A). Changing the reaction temperature also affected the product formation rate during the steady-state regime (Figure S3B).

Despite the marked differences between the two reaction regimes, the product distribution remained largely constant (Figure 2B). The constant product distribution strongly indicates that both reaction regimes follow the same mechanism. We observed that C_4_ and C_6_ were almost equimolar and consistently the major products (∼40 mol % each). Notably, the alkane products were predominantly monobranched, i.e., isobutane was the only C_4_ alkane detected, while 2-methylpentane and 3-methylpentane contributed to 99% of the C_6_ alkanes, with a molar ratio of approximately 3:1. *n*-Hexane was also detected but in negligible concentrations (<1 mol % of total C_6_). It should be noted that the disproportionation products retained the same branching degree as the starting material, isopentane.

Besides C_4_ and C_6_ alkanes, minor concentrations of C_7_ to C_10_ alkanes were also observed, with their concentrations decreasing sequentially. Ethane or propane was not detected, and alkanes with carbon numbers higher than 10 were detected only in negligible concentrations (<1 mol % of total product). Hence, we will not address these products in the following discussion. It is hypothesized that these minor products originate from secondary disproportionation steps. For example, the disproportionation product C_6_ can undergo a second disproportionation cycle with *i*C_5_ to yield C_4_ and C_7_. That explains tentatively why a slight decrease in the ratio of C_6_ alkane products and a corresponding increase in the ratio of C_4_ and C_7_ alkane products was observed after 10 min of reaction (Figure 2B). The secondary disproportionation was suppressed when isopentane was in excess (Figure S4C).

In principle, it could be possible that C_8_–C_10_ alkanes are formed via direct alkylation between *i*C_4_^+^ or *i*C_5_^+^ (denoting the tertiary carbenium ion, e.g., *i*C_4_^+^ and *i*C_5_^+^ for the *tert*-butyl and pentyl carbenium ion, respectively) and an alkene (*i*C_4_^=^ and *i*C_5_^=^) followed by a hydride transfer before the alkylate cracks. This scenario, however, would predict a marked increase in the concentration of C_10_ products, reflecting the dominant presence of *i*C_5_. Thus, we conclude that direct alkylation, if it occurs, contributes only to a negligible extent. Thus, isopentane disproportionation remains the predominant reaction. The relatively unchanged product distribution across the reaction suggests that the two kinetic regimes follow the same disproportionation mechanism.

The conclusion leads to the question why the disproportionation rate decreases markedly after the initial regime. The most straightforward hypothesis would be catalyst deactivation, i.e., the loss of catalytically active chloroaluminate species. To analyze this, we address the formation and reactivity of carbenium ions. The initial carbenium ions are formed by chloride abstraction from TBC by the transient aluminum trichloride species (AlCl_3_*), formed from the dissociation of Al_2_Cl_7_^–^ (Al_2_Cl_7_^–^ + Cl-*i*C_4_ ⇌ AlCl_4_^–^ + AlCl_3_···Cl···*i*C_4_).^30^ It should be noted in passing that the equilibrium constant requires a molar ratio close to 1:2 of 1-ethyl-3-methylimidazolium chloride and AlCl_3_ to facilitate AlCl_3_* dissociation from Al_2_Cl_7_^–^ in the ionic liquid.^30,43^ From the resulting AlCl_3_-TBC adduct (AlCl_3_···Cl···*i*C_4_), the carbenium ion is concluded to be surrounded and stabilized by the corresponding AlCl_4_^–^ anion, thus, forming a reactive ion-pair (*i*C_4_^+^-AlCl_4_^–^).^43^ The initial carbenium ion-pair (*i*C_4_^+^-AlCl_4_^–^), activates reacting molecules via consecutive hydride transfer. The resulting *t-*Alk^+^-AlCl_4_^–^ ion-pairs are the most abundant reactive intermediates (MARI; see Note S1 for details).^43,44^ Formation of the carbenium ion and the disproportionation can also be directly initiated by anhydrous AlCl_3_ and TBC (Figure S5), exhibiting comparable kinetic behavior as described above. The chloride abstraction from TBC may alter the chloroaluminate species in the ionic liquid. We characterized the chloroaluminate species in the ionic liquid by Raman and ^27^Al NMR spectroscopy to estimate its abundance. Both methods showed that the chloroaluminate species did not change with reaction time, nor with the amount of TBC added (Figures S6 and S7), ruling out the possibility of the loss of Lewis acidc chloroaluminate species as the cause of the lower reaction rate. The concentration of the active chloroaluminate species remained constant throughout the entire reaction. Additionally, the fact that the chloroaluminate species is unaffected by the TBC amount in the reaction mixture suggests that the addition of TBC, at least in the amounts tested, does not significantly influence the dissociation equilibrium Al_2_Cl_7_^–^ ⇌ AlCl_4_^–^ + AlCl_3_*.

### 2.3 On the Reaction Mechanism for Isopentane Disproportionation

The identical product distribution and the significant rate differences between the two reaction regimes strongly point to an identical bimolecular mechanism but with different concentrations of the limiting species. Figure 3 illustrates the interconnected reaction steps.

**Figure 3:**
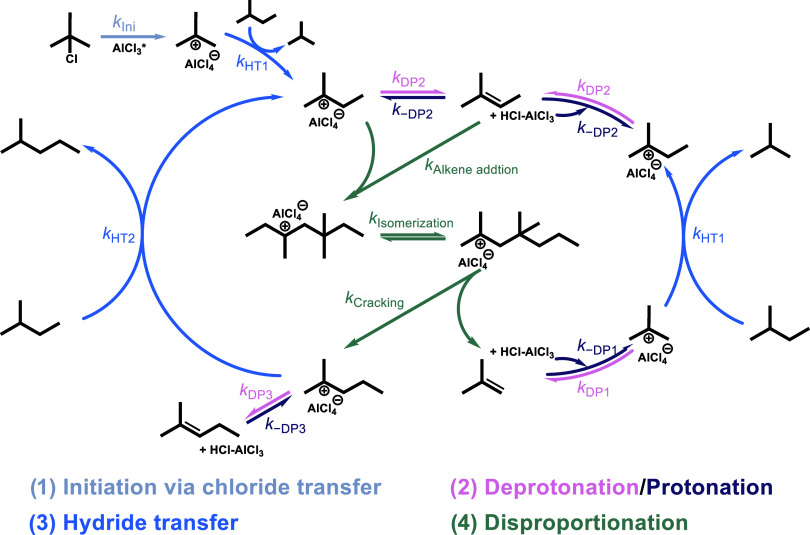
Reaction mechanism for isopentane disproportionation. The complete reaction network consists of reactions categorized into four subgroups, each indicated by a corresponding color: Initiation via chloride transfer (gray), deprotonation/protonation (pink/purple), hydride transfer (blue), and disproportionation (green). The formation of C_6_ carbenium ion and C_4_ alkene is depicted as one possibility of the β-scission.

Chloride and hydride transfer are critical to form the initial carbenium ions. The mechanism begins with the formation of the *i*C_4_^+^-AlCl_4_^–^ ion-pair through chloride transfer between TBC and AlCl_3_* (top left corner in Figure 3, gray arrow, *k*_Ini_). Subsequently, *i*C_4_^+^-AlCl_4_^–^ undergoes hydride transfer, abstracting a hydride from isopentane to yield isobutane and *i*C_5_^+^-AlCl_4_^–^ (blue arrow, *k*_HT1_). Theoretical calculations (Figure S19) strongly suggest a complete chloride transfer to AlCl_4_^–^, resulting in an ion-pair between *i*C_4_^+^ and AlCl_4_^–^, in contrast to earlier studies proposing a TBC-AlCl_3_ adduct.^43^ The reaction mechanism is, however, independent of this question. In accordance with theory, we use carbenium ion-AlCl_4_^–^ ion-pairs in Figure 3 and throughout the text.

A key step in the disproportionation mechanism is the deprotonation of the *i*C_5_^+^-AlCl_4_^–^ ion-pair (pink arrow, *k*_DP2_), which leads to the formation of the *i*C_5_ alkene and an HCl-chloroaluminate species.^45,46^ Theory indicates that H^+^(AlCl_4_^–^)_2_ is the deprotonation intermediate (Figure 7A), but the exact end products will be subject to further studies. For the sake of brevity, HCl-chloroaluminate species are depicted as HCl-AlCl_3_ in Figure 3.

Returning to the mechanism, the *i*C_5_ alkenes react readily with *i*C_5_^+^-AlCl_4_^–^ to yield C_10_^+^-AlCl_4_^–^ (green arrow, *k*_Alkene addition_). The primary product must undergo skeletal isomerization (green arrow, *k*_Isomerization_)^31,47,48^ and then crack via β-scission (green arrow, *k*_Cracking_), forming *i*C_6_^+^-AlCl_4_^–^ and isobutene. Next, in the left half-cycle of Figure 3, hydride transfer from isopentane to *i*C_6_^+^-AlCl_4_^–^ produces *i*C_6_ and regenerates *i*C_5_^+^-AlCl_4_^–^ to close the cycle (blue arrow, *k*_HT2_). In the right half-cycle, isobutene is protonated (purple arrow, *k*_–DP1_) to form *i*C_4_^+^-AlCl_4_^–^, which reacts with isopentane to yield *i*C_4_ as product and regenerate *i*C_5_^+^-AlCl_4_^–^ (blue arrow, *k*_HT1_). It is worth mentioning that *i*C_4_^+^ and *i*C_6_ alkene could also be formed in the cracking step without impacting the mechanism or product distribution.

Similar mechanisms have been reported for isobutane disproportionation on Brønsted acidic zeolites^49^ and *n*-butane disproportionation catalyzed by aluminum chloride/sulfonic acid resin.^50^ Both processes also yield equimolar amounts of C_3_ and C_5_ alkanes.

It is important to emphasize that the concentration of carbenium ions (ion-pairs) governs the rate of the hydride transfer for a given catalyst. In Figure 3, all deprotonation equilibria are incorporated in the catalytic cycle except for the one involving the *i*C_6_^+^-AlCl_4_^–^ ion-pair (bottom left corner, pink arrow *k*_DP3_ and purple arrow *k*_–DP3_). We propose that this off-cycle equilibrium is established in the steady-state regime, favoring the alkene formation, which diminishes the concentration of carbenium ions and reduces the overall reaction rate.

As alkenes are not detected among the products, all alkenes are only intermediates that are eventually converted into the corresponding alkanes. In the protonation step, the alkene is protonated by the HCl-chloroaluminate species, reforming the carbenium ion-AlCl_4_^–^ ion-pair (C^=^ + HCl-AlCl_3_ ⇌ C^+^-AlCl_4_^–^). This ion-pair subsequently undergoes hydride transfer with isopentane, resulting in the formation of the alkane product, i.e., C^+^-AlCl_4_^–^ + *i*C_5_ → C (alkane product) + *i*C_5_–AlCl_4_^–^. Additionally, the low concentration of products with a carbon number greater than 10 indicates that multiple alkene addition is very limited under our reaction conditions (Figure 2B).

The characterization of the alkene intermediates is discussed in the subsequent section.

### 2.4 Alkene Formation in the Isopentane Disproportionation

*In situ* ^1^H NMR spectroscopy was used to analyze alkene formation. The presence of alkenes was proportional to the TBC concentration. The middle panel in Figure 4 shows the key alkene regions of the collected *in situ* ^1^H NMR spectra. The peak at 5.14 ppm, formed during the reaction, is attributed to the vinylic proton of 2-methyl-2-pentene (5.16 ppm, Figure S11). The peak at 2.30 ppm corresponds to the allylic proton. The assignment of the allylic proton is further validated by the 2D ^1^H–^13^C HMBC (two-dimensional heteronuclear multiple bond correlation) spectroscopy (^13^C cross-peaks with chemical shift between 140 and 155 ppm, see Figure S13). The peak integral of the standard was used to normalize the two alkene-related peaks. Since the same standard was used for all measurements, the normalized peak integrals can be compared across different experiments (see the Supporting Information for Experimental Details). The left and right plots in Figure 4 show the linear correlation between the TBC amount and the integrals of the vinylic (5.14 ppm) and allylic (2.30 ppm) peaks, respectively. The ^1^H NMR results show alkene formation observed in the reaction, which is proportional to the TBC amount, agreeing with the above postulated reaction pathway.

**Figure 4:**
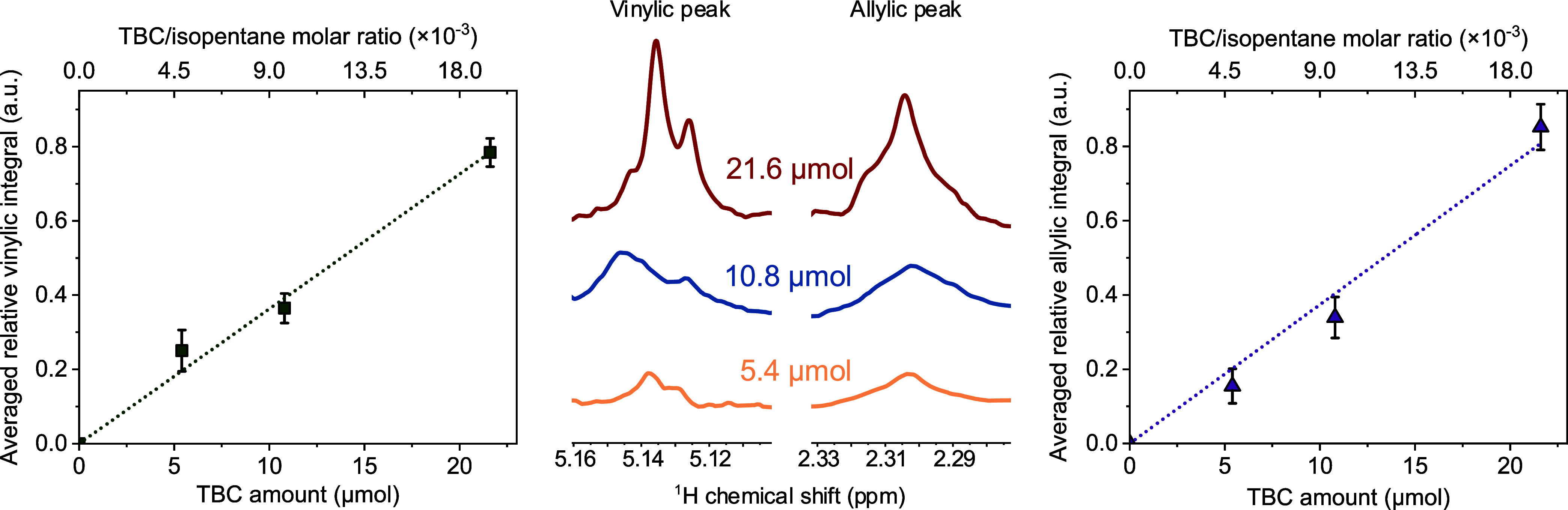
^1^H NMR spectroscopy of isopentane disproportionation reaction to probe alkene formation. Key regions of the ^1^H NMR spectra recorded in the presence of varying amounts of TBC. Key regions include peaks at 5.14 ppm (vinylic proton, peak integrals on the left) and 2.30 ppm (allylic proton, peak integrals on the right). Averaged relative peak integrals (Integral_alkene_/Integral_benzene_) from measurements with varying amounts of TBC are plotted against the TBC amounts. The top axis displays the corresponding molar ratio of TBC to isopentane. The averaged relative integrals are calculated using data points at a constant level. Reaction conditions: AlCl_3_-saturated dichloromethane-*d*_2_, 0.6 mL; *i*C_5_, 1.1 mmol (80 mg); TBC, 5.4 μmol (0.5 mg), 10.8 μmol (1 mg), or 21.6 μmol (2 mg); and temperature, 25 °C. Equivalent actual reaction conditions: ionic liquid, 1 mmol; dichloromethane, 3 mL; *i*C_5_, 5.5 mmol (400 mg); TBC, 0.027 mmol (2.5 mg), 0.054 mmol (5 mg), or 0.11 mmol (10 mg).

### 2.5 Kinetic Models for Isopentane Disproportionation

To reconcile the experimental time-resolved results with the proposed mechanism delineated in Figure 3, we constructed and fitted kinetic models using the COPASI software suite.^51^ While other more complex models, comprising up to 10 kinetic parameters, were initially considered (Figure S14), we successfully refined a core kinetic model containing only four rate constants. The validity of the core model is demonstrated by the close fit between the simulated kinetics and the time-resolved profiles at different amounts of the carbenium ion initiator TBC (Figures 5B and S15A). Accordingly, we analyzed the fitted core kinetic model to provide an interpretation for how this reaction system induces the observed kinetic behavior for isopentane disproportionation, consisting of the aforementioned transient and steady-state regimes.

**Figure 5:**
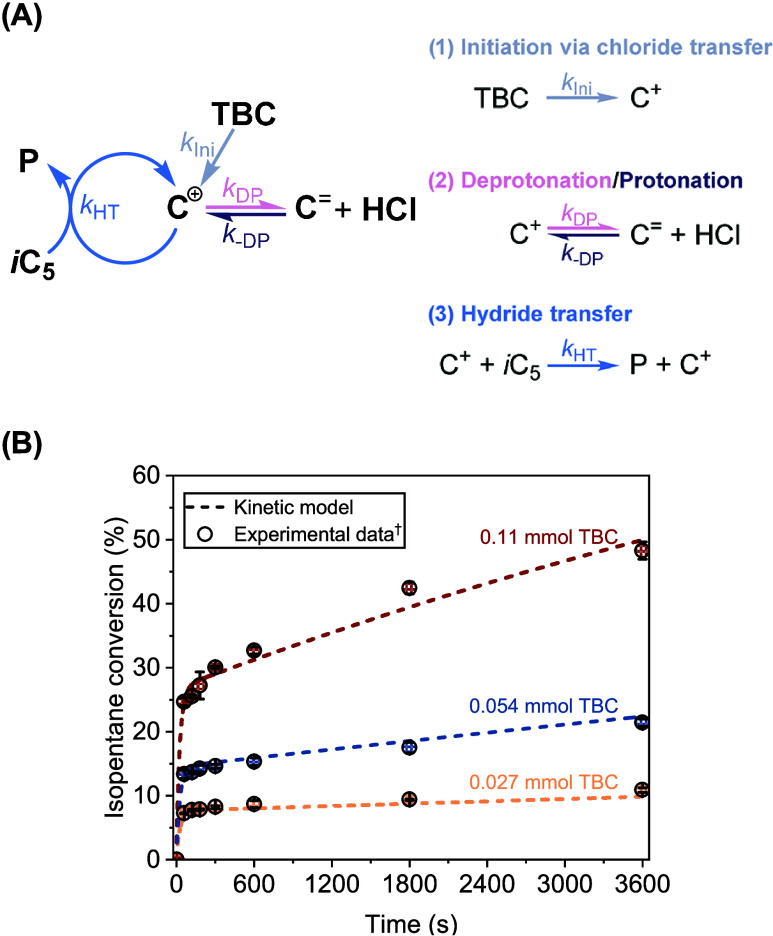
Core kinetic model of isopentane disproportionation reaction constructed in COPASI. (A) Reaction network and the corresponding elementary steps of the core kinetic model. The species are abbreviated as follows: C^+^ represents the C^+^-AlCl_4_^–^ ion-pair; HCl, the HCl-chloroaluminate species; and P, the alkane products. (B) Experimental data (points, ^†^from Figure S1A) and COPASI-simulated results (dashed lines) of isopentane conversion with varying TBC amounts. Reaction conditions of the experimental data: *i*C_5_, 5.5 mmol (400 mg); ionic liquid, 1 mmol; TBC, 0.11 mmol (10 mg, red), 0.054 mmol (5 mg, blue), or 0.027 mmol (2.5 mg, peach); dichloromethane, 3 mL; and temperature, 25 °C. Time expressed in seconds for more precise tracking of the reaction dynamics.

Owing to its simplicity, this core model offers the clearest and most direct insight into the mechanism, revealing that the kinetic behavior of this system is determined by three rate constants: (i) that of deprotonation (*k*_DP_), which formally converts carbenium ion equivalents to alkenes and HCl-chloroaluminate species, (ii) its microscopic reverse (*k*_–DP_), which regenerates carbenium ions from alkenes, and (iii) that of hydride transfer (*k*_HT_), which controls the overall rate at which the carbenium ion and alkene chain carriers mediate the conversion of isopentane to disproportionation products.

Further analysis of the mechanistic models leads to five key insights. (1) Initiation occurs via chloride abstraction from TBC to generate carbenium ion chain carrier species. It occurs rapidly and quantitatively, such that the corresponding kinetic parameter (*k*_Ini_) does not influence the observed time-course behavior. (2) The disproportionation reaction is mediated by both carbenium ion and alkene chain carrier species, which are generated *in situ* from the carbenium ion initiator TBC, and interconverted via a reversible deprotonation/protonation process. It follows that efficient disproportionation will occur when the proportions of these two chain carrier species are such that the system is not depleted of either one of them. Note that the presented model just invokes alkene formation as an off-cycle equilibrium, while the more complex mechanistic models properly reconcile these two species as active chain carriers (see the Supporting Information for additional discussion on the more complex mechanistic proposals). (3) The disproportionation rate during the steady-state regime is established once the reversible interconversion of chain carrier species reaches equilibrium. It is, therefore, determined by *K*_DP_, as the ratio of the constituent rate constants *k*_DP_ and *k*_–DP_. As corroborated by the observation of alkene species in concentrations proportional to [TBC]_0_ by NMR spectroscopy, this equilibrium heavily favors alkenes. Consequently, the slow steady-state conversion rate results from a scarcity of carbenium ion chain carriers in the reaction system (Figure S15B). (4) The transient regime corresponds to a nonequilibrium state of the reversible deprotonation/protonation process, that lies between the excess of carbenium ions initially generated from TBC, and the depletion of carbenium ions as the reversible interconversion process reaches equilibrium. The duration of the transient regime, and thus the total conversion to disproportionation products achieved during this regime, is inversely proportional to *k*_DP_, which determines how quickly carbenium ions are depleted en route to equilibrium (Figure S15C,D). (5) For a given proportion of the two chain carrier species, the rate at which the isopentane substrate is converted to disproportionation products is determined by a single kinetic parameter (*k*_HT_), attributed to rate-determining hydride transfer from *i*C_5_ to C*_n_* ^+^ (*n* ≠ 5). Therefore, an increase in *k*_HT_ will result in a proportional increase in the rate of *i*C_5_ disproportionation, in both the transient and steady-state regimes.

Taken together, the rate of disproportionation during the steady-state regime is determined by the position of the equilibrium that interconverts the carbenium ion and alkene chain carrier species, as well as the rate at which the hydride transfer step proceeds. Increasing the kinetic parameters *k*_HT_ and *k*_–DP_ serves to accelerate *i*C_5_ disproportionation, while increasing *k*_DP_ attenuates this reactivity. However, the rate of disproportionation during the steady-state regime is strongly suppressed relative to that of the transient regime, during which nonequilibrium proportions of carbenium ion and alkene chain carriers effectuate rapid conversion. During the transient regime, the amount of conversion to disproportionation products attained depends not only on the reaction rate but also on its duration, which is determined by how fast the deprotonation/protonation process reaches equilibrium. Accordingly, the conversion achieved during the transient regime is proportional to *k*_HT_, inversely proportional to *k*_DP_, and largely unaffected by *k*_–DP_, since *k*_–DP_ determines only the position of the equilibrium, and not how quickly it is reached.

The influence of *k*_HT_, *k*_DP_, and *k*_–DP_ on the kinetic behavior of the system in the transient and steady-state regimes was corroborated by performing sensitivity analyses on the mechanistic model (Figure S15E,F), and is captured by the following empirical kinetic equations (see detailed derivations in Note S2)­

1 $$r\left(t\right)\mathrm{transient regime}=k_{\mathrm{HT}}\cdot {\left[\mathrm{TBC}\right]}_{0}\cdot e^{-k_{\mathrm{DP}}\cdot t}$$

2 $${y\mathrm{ield}}_{\mathrm{transient regime}}=\frac{k_{\mathrm{HT}}{\cdot \left[\mathrm{TBC}\right]}_{0}}{k_{\mathrm{DP}}}\left(1-e^{-k_{\mathrm{DP}}\cdot t}\right)$$

3 $$r_{\mathrm{steady}-\mathrm{state regime}}=\frac{{{k_{\mathrm{HT}}\cdot k_{-\mathrm{DP}}\cdot \left[\mathrm{TBC}\right]}_{0}}^{2}}{k_{\mathrm{DP}}}$$

The elementary steps, their corresponding differential equations, and the best-fit rate constants defining the kinetic model are provided in Table S1. Notably, this demonstrates that the disproportionation reaction, when analyzed with the presented kinetic model, could serve as a robust method for evaluating the hydride transfer ability of a catalyst.

### 2.6 Activation Energy of Isopentane Disproportionation in the Steady-State Regime

The temperature dependencies of the transient and steady-state regimes differ due to their distinct influences from rate constants. In the transient regime, only the hydride transfer and deprotonation rate constants influence. A lack of temperature effect on conversion is observed during this regime (Figure S2C). Our hypothesis suggests that as temperature changes, the rate constants for both hydride transfer and deprotonation increase, which accelerates product formation but shortens this period, keeping the conversion constant, as suggested by the kinetic model (Figure S15C). In the steady-state regime, however, the product formation rate becomes temperature-dependent (Figure S3B) due to the additional influence of the rate constant *k*_–DP_. From the sensitivity analysis of the kinetic model (Figure S15F) and the derived rate eq (eq 3), we obtained the relation, *r*_steady‑state regime_ ∝ *k*_HT_/*K*_DP_, leads to the effective activation energy of the steady-state regime being expressed as *E*_a,steady‑state regime_ = *E*_a,HT_ – *E*_a,DP_ + *E*_a,–DP_. Notably, this is not a direct linear combination of the individual *E*_a_ values, but rather a result of their convolution through the exponential dependencies described by the Arrhenius equation. The determined effective activation energy for the steady-state regime is 54 kJ/mol (Figure 6).

**Figure 6:**
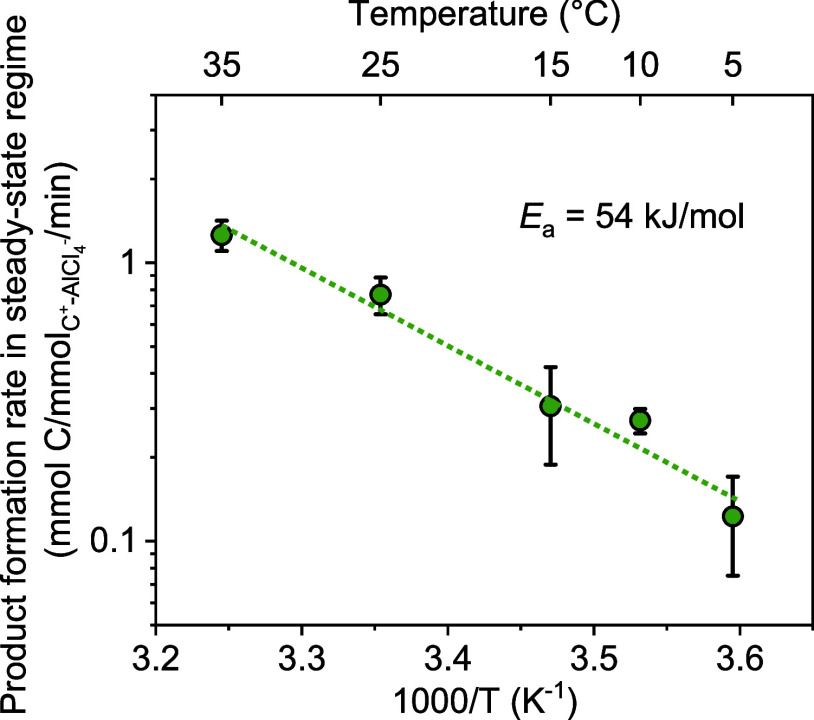
Arrhenius plot for isopentane disproportionation in the steady-state regime. Reaction conditions: *i*C_5_, 5.5 mmol (400 mg); ionic liquid, 1 mmol; TBC, 0.054 mmol (5 mg); dichloromethane, 3 mL; and temperature, 5–35 °C. Product formation rates normalized to the active species C^+^-AlCl_4_^–^ (see Procedure for liquid phase product analysis of isopentane disproportionation in Supporting Information).

### 2.7 Atomistic Modeling of the Deprotonation Mechanism

We utilized density functional theory (DFT)-based *ab initio* molecular dynamics (AIMD) simulations coupled with a Blue Moon ensemble method to gain insights into the deprotonation pathway. A periodic cell was constructed to mirror experimental conditions of the reaction solution (see Supporting Information for details) and equilibrated at 298 K. With DFT calculations, we aim to substantiate some of our mechanistic hypotheses, namely (i) the chloride abstraction, the initiation step that generates the active species, is kinetically facile and irreversible; (ii) the kinetic behavior of the disproportionation reaction, comprising a transient- and steady-state regime, stipulates that the deprotonation process must be associated with a significant free energy barrier; (iii) the different alkenes and carbenium ions in our system (namely, *i*C_4_^=^/*i*C_4_^+^, *i*C_5_^=^/*i*C_5_^+^, and *i*C_6_^=^/*i*C_6_^+^) might exhibit different reactivity with respect to protonation/deprotonation, leading to one being favored as the off-cycle species over the others.

We first probe the chloride abstraction. During the simulation, the initiator TBC readily transfers its chloride to an AlCl_3_*, forming an *i*C_4_^+^-AlCl_4_^–^ ion-pair. Based on the simulated trajectory, this interaction is purely ionic, with AlCl_4_^–^ weakly bound to the carbenium ion. For example, Figure S21 illustrates the rotational freedom of AlCl_4_^–^, showing the exchange of Cl atoms coordinating the *i*C_4_^+^ carbenium. From Figure S19, the energy barrier to separate the AlCl_4_^–^ and *i*C_4_^+^ ion-pair is ca. 8 kJ/mol. This validates our mechanistic hypothesis of a chloride abstraction which is facile and irreversible. Furthermore, the purely ionic interaction between AlCl_4_^–^ and the carbenium ion indicates that a decrease in carbenium ion concentration due to reformation of chloroalkane species (e.g., TBC) is highly unlikely. As such, the decrease in the carbenium ion concentration as the reaction enters the steady-state regime is more likely due to the deprotonation.

Subsequently, we investigated the deprotonation mechanism. Previous work indicates that deprotonation will occur through the formation of an HAlCl_4_ (or HCl-AlCl_3_) species,^45^ and that HAlCl_4_ can exist in Lewis acidic ionic liquid.^52^ As such, we calculated the energetics of the deprotonation of *i*C_4_^+^ by AlCl_4_^–^ using the Blue Moon ensemble method. As depicted in Figure S18, this process is energetically uphill, with no intermediate minimum corresponding to the HAlCl_4_ species. These results suggest that AlCl_4_^–^ does not independently mediate the deprotonation of *i*C_4_^+^. Instead, our results indicate that two AlCl_4_^–^ molecules facilitate deprotonation. Together, they deprotonate *i*C_4_^+^, forming isobutene while sharing the resulting proton to create an H^+^(AlCl_4_^–^)_2_ complex (Figure 7A). The free energy barrier for this deprotonation step is calculated to be 104 kJ/mol. The H^+^(AlCl_4_^–^)_2_ complex is most likely a transient species, as it has been previously shown to readily recombine to produce Al_2_Cl_7_^–^ and HCl.^53^ Our results suggest that the free energy profile of the deprotonation is dependent on the chloroaluminate species that participates in this process. The energy barrier of 104 kJ/mol suggests that the deprotonation step does not occur rapidly. This aligns with our conclusion from the kinetic models that *k*_DP_ cannot be large, which would otherwise render the transient regime insignificant (Figure S15D).

**Figure 7:**
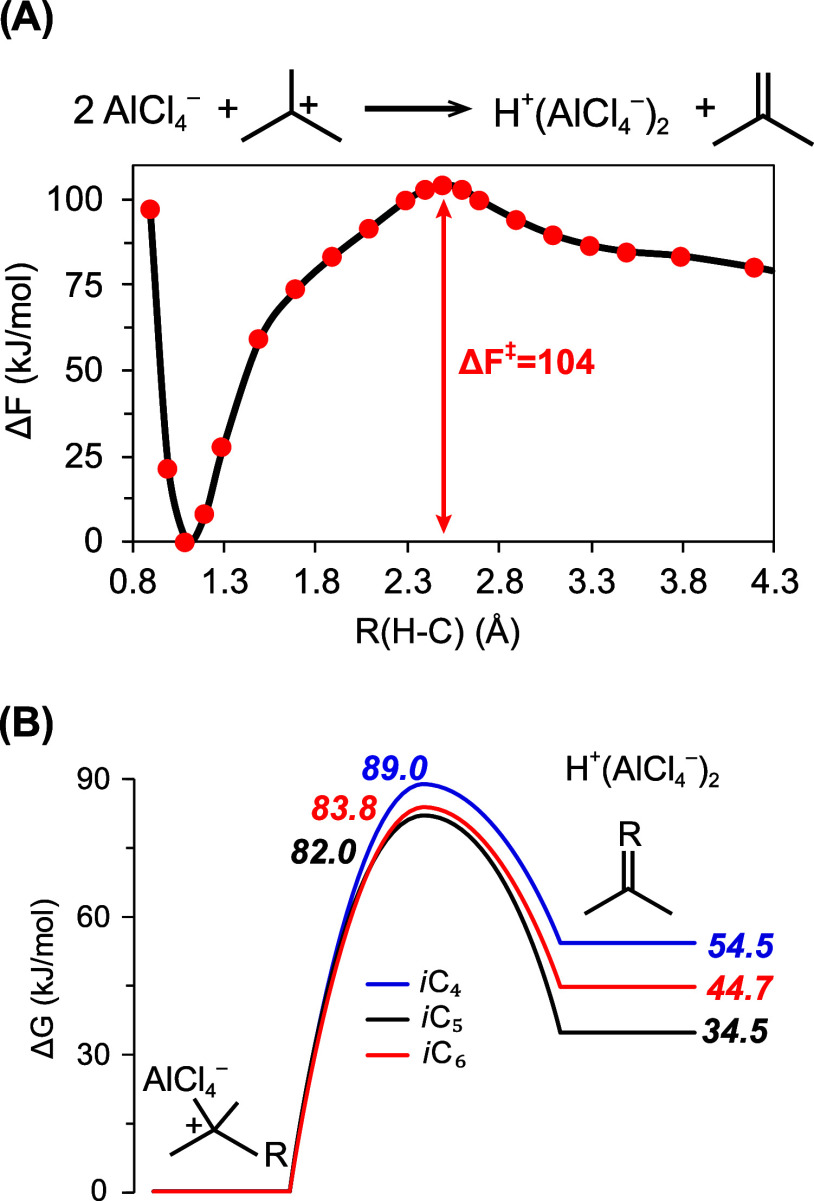
Computed reaction energetics of deprotonation. (A) Helmholtz free energy (Δ*F*) as a function of the internuclear distance of C–H for the reaction between AlCl_4_^–^ and *i*C_4_^+^ calculated under simulated reaction conditions using the AIMD-based Blue Moon ensemble approach. Here, C is a terminal −CH_3_ and H the resulting proton which is removed from the C resulting in the H^+^(AlCl_4_^–^)_2_ species. (B) Gibbs free energy (Δ*G*) for the isolated, gas-phase reaction of 2 AlCl_4_^–^ with *i*C_4_^+^, *i*C_5_^+^, or *i*C_6_^+^.

Lastly, we investigate the reactivities of different carbenium ion-AlCl_4_^–^ ion-pairs with respect to deprotonation. Based on the deprotonation mechanism observed for TBC in the reaction solution, we performed DFT calculations to evaluate the energetics of deprotonation for *i*C_4_^+^, *i*C_5_^+^, and *i*C_6_^+^ (as carbenium ion-AlCl_4_^–^ ion-pairs). These calculations were performed on isolated, gas-phase molecules and their relative energetics are shown in Figure 7B. For *i*C_4_^+^, *i*C_5_^+^, and *i*C_6_^+^, the gas-phase calculations show a free energy barrier of 89, 82, and 84 kJ/mol, respectively. Notably, this barrier decreases by 5–7 kJ/mol for *i*C_5_^+^ and *i*C_6_^+^, suggesting that deprotonation of both species is more favorable than that of TBC under experimental conditions. In addition, the Δ*G*° of the products indicates *i*C_5_^=^, and *i*C_6_^=^ are 20 and 9.8 kJ/mol more stable than *i*C_4_^=^, respectively. These results may explain why only *i*C_6_^=^ is observed as an accumulated alkene (off-cycle alkene pool), while *i*C_4_^=^ is not (Figure 4).

To summarize, our calculations indicate that (i) deprotonation should account for the decrease of carbenium ion concentration and the corresponding overall decrease in the rate of disproportionation, while the reformation of chloroalkanes is not responsible for the observed kinetic behavior; (ii) deprotonation is not a rapid process, and thus takes some time to establish an equilibrium between carbenium ions and alkenes, giving rise to the transition from the transient regime to the steady-state regime; and (iii) *i*C_6_^=^ may be favored as the alkene species over *i*C_4_^=^ to be formed through deprotonation, and accumulated as off-cycle alkene.

## 3 Conclusion

*Tert*-butyl chloride (TBC) as an initial carbenium ion generator starts isopentane disproportionation catalyzed by the Lewis acidic chloroaluminate ionic liquid in dichloromethane, predominantly producing equimolar amounts of isobutane and methylpentanes. This disproportionation occurs rapidly at the beginning of the reaction, leading to an initial burst of isopentane conversion. The extent of this initial isopentane conversion is directly proportional to the concentration of TBC added. After the initial burst, isopentane disproportionation continues but at a rate that is slower by an order of magnitude.

Thus, the isopentane disproportionation reaction is divided into two regimes, the transient regime (up to 5 min of contact with the catalyst) and the subsequent steady-state regime. The transient regime is characterized by a high rate of isopentane conversion. The conversion achieved in this regime correlates linearly with the initial carbenium ion concentration and remains unaffected by other experimental parameters. The initial carbenium ions, generated from the reaction between TBC and AlCl_3_, exist as carbenium ion-AlCl_4_^–^ ion-pairs and act as the active chain-carrying species in the reaction. In contrast, the steady-state regime proceeds at a lower rate, with the isopentane conversion rate influenced by the carbenium ion concentration, the ionic liquid amount, and the temperature. Despite the differing rates, both regimes follow the same disproportionation mechanism. Catalyst deactivation does not occur throughout the reaction; however, the carbenium ion concentration decreases. The significant decrease in conversion rate is hypothesized to be due to the deprotonation equilibrium, which transforms the carbenium ions into alkenes, thereby reducing the concentration of carbenium ions.

The presence of alkenes in the isopentane disproportionation reaction was probed via ^1^H NMR spectroscopy. A vinylic peak was observed at 5.14 ppm, corresponding to the vinylic proton from 2-methyl-2-pentene. An allylic peak at 2.3 ppm was also detected, confirmed by a corresponding ^13^C peak at 154 ppm. The intensities of these peaks were linearly correlated with the amount of TBC added, supporting the hypothesis that alkenes are formed from carbenium ion-AlCl_4_^–^ ion-pairs through deprotonation.

Kinetic modeling identified the parameters governing the two kinetic regimes. The transient regime is positively influenced by the hydride transfer rate constant *k*_HT_ and negatively by the deprotonation rate constant *k*_DP_. This relationship is expressed as Yield_transient regime_ ∝ *k*_HT_/*k*_DP_. The steady-state regime is influenced by these rate constants and is additionally positively affected by the protonation rate constant *k*_–DP_. Thus, the derived expression for the activation energy of the steady-state regime is *E*_a,steady‑state regime_ = *E*_a,HT_ – *E*_a,DP_ + *E*_a,–DP_, yielding a value of 54 kJ/mol.

In summary, this study elucidates the intricacies of isopentane disproportionation, providing fundamental insights into the carbenium ion mechanisms that are central to Lewis acid-catalyzed hydrocarbon conversions. It serves as an effective model system, showing how carbenium ions are initiated and stabilized in more complex processes like alkane isomerization, alkylation, and the LDPE tandem conversion. Moreover, the dominating role of the hydride transfer rate constant indicates that this reaction is a suitable probe reaction for hydride transfer in general.

## Supplementary Material



## Notes

Experimental procedures, computational methods and results, characterization of active chloroaluminate species (NMR and Raman spectroscopy), kinetics data, COPASI modeling results and description, derivation of rate equations (PDF)

## §

State Key Laboratory of Petroleum Molecular and Process Engineering, Shanghai Key Laboratory of Green Chemistry and Chemical Processes, School of Chemistry and Molecular Engineering, East China Normal University, Shanghai 200062, China

## ∥

Materials Science Division, Argonne National Laboratory, Lemont, Illinois 60439, United States
