# Supplementary material for: Isopentane Disproportionation in Lewis Acidic Chloroaluminate Ionic Liquid
Source: J Am Chem Soc. 2026 Jun 17;148(25):25887–97. doi: 10.1021/jacs.6c03060 (PMC13339624; doi:10.1021/jacs.6c03060)
Supplement: Supplementary file 1 [file ja6c03060_si_001.pdf]

Supporting Information

Isopentane disproportionation in Lewis acidic chloroaluminate ionic liquid

Jiande Mai[1,2], David E. Ryan[1,2], Wei Zhang[1,2,†], Benjamin A. Jackson[1,2], Kiyoung Jo[2,‡], Janos Szanyi[1,2], Oliver Y. Gutiérrez[1,2], Honghong Shi[1], Donald M. Camaioni[1,2], Rachit Khare[3], Mal-Soon Lee[1,2], Huamin Wang[1], Sungmin Kim[1,2*], and Johannes A. Lercher[1,3*]

[1] *Institute for Integrated Catalysis, Pacific Northwest National Laboratory, Richland, Washington 99354, United States*
[2] *Physical and Computational Sciences Directorate, Pacific Northwest National Laboratory, Richland, Washington 99354, United States*
[3] *Department of Chemistry and Catalysis Research Center, TU München, Garching 85748, Germany*

[†] *Present address: State Key Laboratory of Petroleum Molecular and Process Engineering, Shanghai Key Laboratory of Green Chemistry and Chemical Processes, School of Chemistry and Molecular Engineering, East China Normal University, Shanghai 200062, China*
[‡] *Present address: Materials Science Division, Argonne National Laboratory, Lemont, Illinois 60439, United States*

Materials and Methods

Chemical and materials

The following chemicals were used as received in this work: 1-ethyl-3-methylimidazolium chloride ([EMIM]Cl, >98.0%, TCI), anhydrous aluminum (III) chloride (99.99%, Sigma-Aldrich), low-density polyethylene (LDPE, average M_w ~4000, average M_n ~1700, Sigma-Aldrich), 2-methylbutane (isopentane, ≥99.5%, Sigma-Aldrich), *tert*-butyl chloride (TBC, ≥99.0%, Sigma-Aldrich), dichloromethane (DCM, anhydrous, ≥99.8%, Sigma-Aldrich), chloroform ($CHCl_3$, anhydrous, ≥99%, Sigma-Aldrich), *trans*-decahydronaphthalene (*trans*-Decalin, 99%, Sigma-Aldrich), sodium hydroxide (≥97.0%, Sigma-Aldrich), dichloromethane-d_2 (99.5 atom % D, Thermo Scientific), 2-methyl-2-butene (>95.0%, TCI), 2-methylpentane (>99.5%, Sigma-Aldrich), 2-methyl-2-pentene (>95.0%, TCI), benzene (>99.0%, Sigma-Aldrich).

Procedure for Lewis acidic 1-ethyl-3-methylimidazolium chloride-aluminum chloride ionic liquid ([EMIM]Cl-2$AlCl_3$) preparation

In a typical synthesis, the [EMIM]Cl-2$AlCl_3$ ionic liquid was prepared by slowly adding anhydrous aluminum chloride to 1-ethyl-3-methylimidazolium chloride at a 2:1 molar ratio under continuous stirring. The mixture was stirred overnight, yielding a light-grey liquid. Both reagents were handled and stored exclusively in a nitrogen-filled glove box to minimize exposure to moisture.

Procedure for the tandem cracking-alkylation reaction of LDPE with isopentane

The tandem cracking-alkylation reaction of LDPE with isopentane follows the same procedure as in our previous work.[1]

In a typical reaction, low-density polyethylene (LDPE, 200 mg), isopentane (iC_5, 11.1 mmol), and *tert*-butyl chloride (TBC, 0.054 or 0.22 mmol) were added into a 34 mL PYREX® borosilicate glass tube (equipped with an open-top screw cap with silicone liner) containing 3 mmol of ionic liquid diluted in dichloromethane (DCM, 3 mL). Then, the tube reactor was heated to 70 °C for the desired reaction time under magnetic stirring (1200 rpm). After the reaction, the tube reactor was first cooled down to room temperature, resulting in a biphasic system with the hydrocarbon products, unreacted swollen polymer, and excess isopentane phase on top and the inorganic ionic liquid and DCM phase on the bottom.

Procedure for the product analysis of the tandem cracking-alkylation reaction of LDPE with isopentane

The product analysis follows our previously reported procedure.[1]

After the reaction, the tube reactor was cooled to −20 °C for 30 min. Then, the gas phase reaction products in the headspace were collected and analyzed by the Inficon Micro GC. After that, an additional 2 mL of isopentane was added as an extracting agent, resulting in a thorough phase separation. Hydrocarbon products ($\geq C_4$) are mainly in the top organic phase, together with minor organic residues in the inorganic ionic liquid on the bottom. The inorganic ionic liquid phase was first transferred to a glass vial and quenched by saturated NaOH aqueous solution at low temperature (−20 °C), and organic residues in the inorganic phase were further extracted by chloroform. All of the collected hydrocarbon products in organic and inorganic phases were mixed with 20 mg of *trans*-decalin as an internal standard and analyzed by GC-FID (Shimadzu, GC-2010) equipped with an HP-5 Agilent column (30 m × 0.32 mm × 0.25 μm), where the relative response factor (RRF) for alkanes of each carbon number and the corresponding retention time were calibrated. The unreacted LDPE was separated from the top phase by a simple filtration to quantify the LDPE conversion:

$$\text{LDPE conversion} = \left(1 - \frac{\text{mass of residual LDPE}}{\text{initial mass of LDPE}}\right) \times 100\%$$

Quantification of the hydrocarbon C_i was determined from the GC-FID signal using internal standard reference:

$$\text{Yield } (C_i, \%) = \left(\frac{\text{mass of } C_i}{\text{initial mass of LDPE}}\right) \times 100\% = \left(\frac{\sum \text{Area } (C_i)}{\text{Area (internal standard)}} \times \frac{\text{mass of internal standard}}{\text{RRF } (C_i)} \times \frac{1}{\text{initial mass of LDPE}}\right) \times 100\%$$

where $\sum$ Area(C_i) was the sum of GC areas of C_i, in which all peaks between nC_{i-1} and nC_i were assumed as branched C_i alkanes. Area (internal standard) was the GC area of the internal standard. The mass balances in all LDPE tandem cracking-alkylation reactions were mostly 90–96%, where product amounts in the headspace and the lower inorganic ionic liquid phase were negligible (< 2 %). The mass loss, therefore, was mostly due to evaporation of light alkane, e.g., isobutane or isopentane (~ 6 % in the mass balance).

Procedure for the isopentane disproportionation

The isopentane disproportionation was conducted at room temperature (25 °C) unless otherwise specified. Our typical reaction mixture consists of isopentane (5.5 mmol, 400 mg), DCM (3 mL), and ionic liquid (1 mmol). The Lewis acidic chloroaluminate ionic liquid consisted of 1-ethyl-3-methylimidazolium chloride and anhydrous aluminum chloride in a 1:2 molar ratio. The amount of TBC varied from 0.025 to 0.1 mmol (2.5 to 10 mg), providing different amounts of initial carbenium ion to the system.

In a typical reaction, 1 mmol of [EMIM]Cl-2$AlCl_3$ (0.413 g) was weighed inside the glove box and transferred to a 34 mL PYREX® borosilicate glass tube (equipped with an open-top screw cap with silicone liner). Next, to the glass tube were added isopentane (5.5 mmol, 400 mg), *tert*-butyl chloride (0.11 mmol, 10 mg) dissolved in DCM (3 mL). The tube reactor was placed in a water bath set at 25 °C under magnetic stirring (1200 rpm) for 1 hour. For kinetic studies, identical reactions were prepared and ran parallel at different time intervals. After the reaction, the tube reactor was removed from the water bath, and the reaction mixture was further processed for product analysis. All experiments were conducted at least in duplicate.

Procedure for headspace product analysis of the isopentane disproportionation

Products in the headspace were quantified using a Micro GC-TCD instrument (Inficon Micro GC Fusion gas analyzer) equipped with four columns (10 m Rt®-Molsieve 5A, 8 m Rt®-U-Bond, 10 m Rt®-Alumina BOND/Na_2SO_4 and 10 m Rxi®-1ms). The instrument was calibrated with a calibrating gas mixture (Gas and Supply, Cat# GSPC26NR150AGC), including, but not limited to, nitrogen, hydrogen, methane, ethane, ethylene, propane, propylene, isobutane, *n*-butane, isobutylene, *n*-pentane, isopentane, 2-methyl-2-butene and *n*-hexane.

For a precise quantification of the headspace composition, desired amounts of ionic liquid, isopentane, *tert*-butyl chloride, and dichloromethane were introduced into a reactor tube inside the glove box (N_2 atmosphere). The disproportionation reaction was allowed to proceed for the prescribed duration inside the glove box. After the reaction, the reaction tube was taken out of the glove box and cooled to −20 °C for 15 min. The reaction tube remained tightly closed throughout the process.

Then, a 1 mL gas-tight analytical syringe (VICI Precision Sampling) was used to collect a portion of the headspace gas through the silicon septum. The gas sample was analyzed by the Inficon Micro GC. The micro thermal conductivity detector (μTCD) detected the eluted separated sample and provided the mole concentration of the component under ambient pressure.

N_2 was used as an internal standard, and its amount remained unchanged. After the reaction, isobutane and isopentane were detected in the headspace. The amount of each alkane in the headspace was calculated using the following equation:

$$\text{Amount of alkane in headspace} = \frac{V_{\text{Headspace}}}{V_m} \cdot \frac{\chi_{\text{alkane}}}{\chi_{N_2}}$$

where $V_{\text{Headspace}}$ was the volume of the headspace (determined to be 30.4 mL), V_m was the molar volume of the ideal gas at 25 °C under ambient pressure (24.5 mL/mmol), χ_{alkane} was the mole concentration of the alkane (isobutane or isopentane) in the headspace after the reaction, and χ_{N2} was the mole concentration of the internal standard N_2 in the headspace after the reaction.

The isobutane and isopentane amounts determined in this manner were found to be negligible across all experiments. This observation is further supported by the good carbon balance obtained in all experiments. Therefore, the headspace product amounts were considered negligible and were excluded from the product quantification in this work.

Procedure for liquid phase product analysis of isopentane disproportionation

After the reaction, the glass reactor was cooled to −20 °C for 15 min. Then, 20 mg *trans*-decalin was added as an internal standard, and 2 mL of chloroform was introduced to ensure thorough mixing and improve the solubility of volatile hydrocarbons (mainly C_4 and C_5). A small amount (ca. 1.2 mL) of the organic mixture was transferred to a 2 mL glass vial and mixed with 0.8 mL saturated NaOH aqueous solution to remove the dissolved ionic liquid. The resulting ionic liquid-free organic phase was further filtered (VWR, hydrophobic PTFE syringe filter) to remove excess water. Then, the sample was quantified by GC-FID (Shimadzu, GC-2010) equipped with an HP-5 Agilent column (30 m × 0.32 mm × 0.25 μm). The relative response factor (RRF) for alkanes of each carbon number and the corresponding retention time were calibrated.

It is important to note that the reaction medium should not be regarded as a neat ionic liquid but rather an ionic liquid/DCM environment. The typical amounts of ionic liquid and isopentane used (1 and 5.5 mmol, respectively) resulted in a homogenous phase. Note that increasing the amounts of either ionic liquid or isopentane can result in a biphasic system, with the hydrocarbon products and unreacted isopentane forming the top organic phase and the ionic liquid dissolved in dichloromethane forming the bottom phase. In such cases, the bottom phase should be removed before adding *trans*-decalin and chloroform.

Since isopentane was the only reactant in the disproportionation reaction, the isopentane conversion was calculated as:

$$\text{Isopentane conversion} = \left(\frac{\text{total mass of alkane products}}{\text{initial mass of isopentane}}\right) \times 100\%$$

The mass of alkane product C_i was calculated as:

$$\text{Mass of alkane product } C_i \text{ (mg)} = \frac{\sum \text{Area } (C_i)}{\text{Area (internal standard)}} \times \frac{\text{mass of internal standard}}{\text{RRF } (C_i)}$$

where $\sum$ Area(C_i) was the sum of GC areas of C_i, in which all peaks between nC_{i-1} and nC_i were assumed as branched C_i alkanes. Area (internal standard) is the GC area of the internal standard.

In the steady-state regime of the disproportionation reaction, the product formation rate was obtained from the linear fit of the product amount versus reaction time. The product amount had the unit of mmol C, defined as total millimoles of carbon atoms in the alkane product. It was calculated as:

$$\text{Product amount (mmol C)} = \sum \left(\frac{\text{mass of alkane product } C_i}{\text{molar mass of } C_i} \times i \right)$$

where i was the carbon number of the collected alkane product.

The product formation rates were further normalized to the catalytically active species amount (C^+-$AlCl_4^-$). Since C^+-$AlCl_4^-$ was formed through the 1:1 interaction between TBC and $Al_2Cl_7^-$, and TBC served as the limiting reagent unless otherwise specified, the amount of C^+-$AlCl_4^-$ was equated to the TBC amount (e.g., 0.027, 0.054, or 0.11 mmol), as determined by the specified reaction conditions. For the transient regime, the averaged product formation rate was determined by dividing the product amount (in mmol C) at 5 minutes by the reaction time of 5 minutes, as a means to quantitatively compare the significant rate disparity between the two kinetic regimes.

The carbon balance of the isopentane disproportionation experiments was calculated as:

$$\text{Carbon balance} = \left(\frac{\text{total mass of alkane products + mass of unreacted isopentane}}{\text{initial mass of isopentane}} \right) \times 100\%$$

where the mass of alkane products and unreacted isopentane were determined from the GC-FID signal, and the initial mass of isopentane was determined by weighing.

The calculated carbon balances for all experiments presented in this work range from 90% to 99%. No solid products were observed in any of the experiments. Light gases in the headspace, isobutane and isopentane, were detected in negligible amounts and were therefore excluded from the mass balance (see details in the headspace analysis). The missing carbon balance primarily results from isopentane evaporation during sample processing. This assertion is supported by comparing the results of two parallel reactions performed under identical reaction conditions and reaction times. In Reaction #1, 408.5 mg of initial isopentane was used, with total alkane products (excluding isopentane) of 38.6 mg detected, and 355.9 mg of unreacted isopentane detected, resulting in a carbon balance of 96.6%. In Reaction #2, 403.6 mg of initial isopentane was used, with 37.6 mg of total alkane products detected (excluding isopentane) and 331.3 mg

of unreacted isopentane detected, resulting in a carbon balance of 91.4%. The discrepancy in carbon balance between the two parallel reactions is attributable to the varying loss of unreacted isopentane during sample processing, which, despite our efforts to minimize it, can occur to a small extent. Importantly, because isopentane conversion was determined based on product formation, this variation in unreacted isopentane does not compromise the integrity of the experimental data. Consequently, we conclude that our experimental data remains accurate even in cases where the carbon balance drops to 90%.

Ex situ **Raman spectroscopy measurements**

Ex situ Raman spectroscopy was used to determine the Al species in the ionic liquid. To obtain a concentrated ionic liquid sample for improved signal detection, larger amounts of ionic liquid and isopentane were used to induce phase separation between the organics and the ionic liquid. A typical sample was prepared as follows: 11.1 mmol (800 mg) isopentane, 2 mmol ionic liquid, 0.054 mmol (5 mg) TBC, and 3 mL DCM were added into the borosilicate glass and allowed to react at room temperature. After the reaction, the glass tube was cooled to −20 °C, and the two phases separated. The bottom ionic liquid phase was transferred to a small glass vial and sealed for the Raman measurement. Raman spectra were measured on a Horiba LabRAM HR Evolution Raman microscope. 785 nm laser excites the sample, and the backscattered signal was collected with ×100 objective lens (Olympus MPlan N NA 0.9) and passed through the 1800 grooves/mm grating before reaching the spectrometer. The laser intensity on the sample was 8.3 mW. Each sample was measured three times (for every measurement, acquisition time: 2 s, accumulation: 30 times). Results were plotted as mean data points with error bars.

Ex situ **^{27}Al NMR spectroscopy measurements**

The *ex situ* ^{27}Al NMR spectroscopy samples were prepared by the same method used to prepare the Raman samples. The ^{27}Al NMR spectroscopy measurements were recorded on a 500 MHz Oxford magnet equipped with a Bruker PI HR-BBO500S2-BBF/H/D-5.0-Z SP probe using standard pulse sequences for ^{27}Al. All measurements were done at 25 °C.

In situ ^{1}H NMR spectroscopy measurements

To avoid interference from ionic liquid cation signals, $AlCl_3$-saturated DCM-d_2 was used in place of the ionic liquid as catalyst. An excess of $AlCl_3$ was added to DCM-d_2 to create a suspension, which was stirred overnight. The undissolved $AlCl_3$ settled, yielding a clear, light-yellow solution. The solution was filtered (VWR, 0.22 µm hydrophobic PTFE syringe filter) to remove all solid $AlCl_3$ and was transferred to a glass vial for temporary storage. All procedures were performed in a glove box.

As the capillary standard, benzene dissolved in DCM-d_2 (concentration: 4.6 µmol/mL) was sealed in a glass capillary. After each measurement, the capillary was removed from the NMR tube, cleaned, and vacuum-dried for reuse.

For a typical *in situ* ^{1}H nuclear magnetic resonance (NMR) spectroscopy measurement, the benzene standard capillary, 0.4 mL of $AlCl_3$-saturated DCM-d_2, and 1.1 mmol (80 mg) of *i*C_5 were loaded into a 5 mm Wilmad NMR tube in the glove box. To initiate the reaction, 21.6 µmol of TBC dissolved in 0.2 mL of DCM-d_2 was added to the sample immediately before measurement. The tube was sealed with a thin strip of parafilm around the seam between the NMR tube and its cap.

The *in situ* 1H NMR spectroscopy measurements were recorded on a 500 MHz Oxford magnet equipped with a Bruker PI HR-BBO500S2-BBF/H/D-5.0-Z SP probe, using standard pulse sequences. T_1 relaxation time constants were determined by inversion recovery (**Figure S8−12**). All acquisition parameters were conservatively optimized for quantitative ^{1}H NMR analysis (d1 ca. 10 max. T_1). The obtained spectra were referenced to the residual DCM solvent signal at 5.36 ppm. A sample spinning rate of 20 Hz was employed to enhance mass transfer within the NMR tube and to produce a high-resolution spectrum. All measurements were done at 25 °C.

2D ^{1}H−^{13}C HSQC and HMBC spectroscopy measurements

For a typical 2D ^{1}H−^{13}C heteronuclear single quantum coherence (HSQC) and heteronuclear multiple bond correlation (HMBC) spectroscopy measurement, 0.4 mL of $AlCl_3$-saturated DCM-d_2 and 1.1 mmol (80 mg) of *i*C_5 were loaded into a 5 mm Wilmad NMR tube in the glove box. 43.2 µmol of TBC dissolved in 0.2 mL of DCM-d_2 was added to the sample. The larger amount of TBC was used to increase the alkene concentration in the reaction system, enabling a more accurate identification.

The 2D ^{1}H−^{13}C HSQC and HMBC spectroscopy measurements were recorded on a 600 MHz Bruker Avance NEO spectrometer equipped with a CryoProbe CP2.1 TCI 600S3 H&F/C/N-D-05 Z XT, using standard pulse sequences. Obtained spectra were referenced to residual DCM solvent signal.

Computational Methods

Reaction free energy calculations in solution

Density functional theory (DFT)-based *ab initio* molecular dynamics (AIMD) simulations were performed on a model system containing 2 1-ethyl-3-methylimidazolium cation, 2 $Al_2Cl_7^-$, 1 *tert*-butyl chloride, 6 isopentane, and 105 dichloromethane molecules within a 23 Å cubic cell, following experimental composition. DFT calculations were performed under periodic boundary conditions and the generalized gradient approximation (GGA) as implemented in CP2K.[2] The Perdew-Burke-Ernzerhoff (PBE)[3] exchange correlation functional was employed. For dispersion corrections the empirical DFT-D3 method of Grimmes *et al.* was used.[4] Valence electrons are expressed in terms of double-ζ quality gaussian basis sets of Goedecker, Teter and Hetter (GTH). Norm-conserving GTH pseudopotentials were employed for the core electrons.[5] An auxiliary plane wave basis set with a cutoff energy of 400 Ry was used for the calculation of the electrostatic terms. The Γ-point approximation is employed for the Brillouin zone integration because of the significant size of the supercell. AIMD simulations were performed under the canonical NVT ensemble at 298 K with a 0.5 fs timestep and Nosé-Hoover chain thermostat of frequency 4000 cm^{-1}.[6] The modeled reaction solution was first equilibrated at 298 K for ~50 ps following which we performed AIMD-based Blue Moon ensemble[7] calculations to obtain the reaction energetics plotted in **Figures S17-S19** by constraining the internuclear distance of two reacting atoms. Helmholtz free energy ΔF was obtained by integration of the ensemble-averaged force due to this constraint along the reaction coordinate.

Reaction free energy calculations in gas-phase

Following the mechanism of $AlCl_4^-$ and iC_4^+, we also investigated the gas-phase reactions of $AlCl_4^-$ with iC_4^+, iC_5^+, or iC_6^+. This was performed first by performing a nudged elastic band calculation (NEB)[8] for the reaction iC_4^+-$AlCl_4^-$ + $AlCl_4^- \rightarrow iC_4^=$ + $H^+(AlCl_4^-)_2$ including 16 images optimized to a force threshold of 3.3E-3 eV/Å. Following this, the NEB transition state was optimized following the Dimer method of Heyden *et al.*[9] The optimized iC_4^+ transition state was used to construct initial guess for the iC_5^+ and iC_6^+ transition state which were similarly optimized. Gas-phase calculations utilized identical settings to the simulated solution with reactants present in an isolated 23 Å cubic cell. Transition states were confirmed by vibrational analysis. Gibbs free energy ΔG was calculated based on the harmonic oscillator approximation.

Thermodynamics of the carbenium–chloroaluminate anion interaction

To determine the thermodynamics for the interaction of iC_4^+ with $AlCl_4^-$ or $Al_2Cl_7^-$, geometry optimizations were performed with the NWChem[10] program at density functional level of theory using the B3LYP functional.[11, 12] The basis set is aug-cc-pVDZ for Al, Cl, and C and cc-pVDZ for H.[13] Harmonic vibrational frequencies were calculated to ensure that the located stationary structures are minima and to obtain zero-point energies. Implicit solvation effects were included through the COSMO model,[14, 15] as implemented in NWChem, and a dielectric constant, ε, of 9.08 was used.

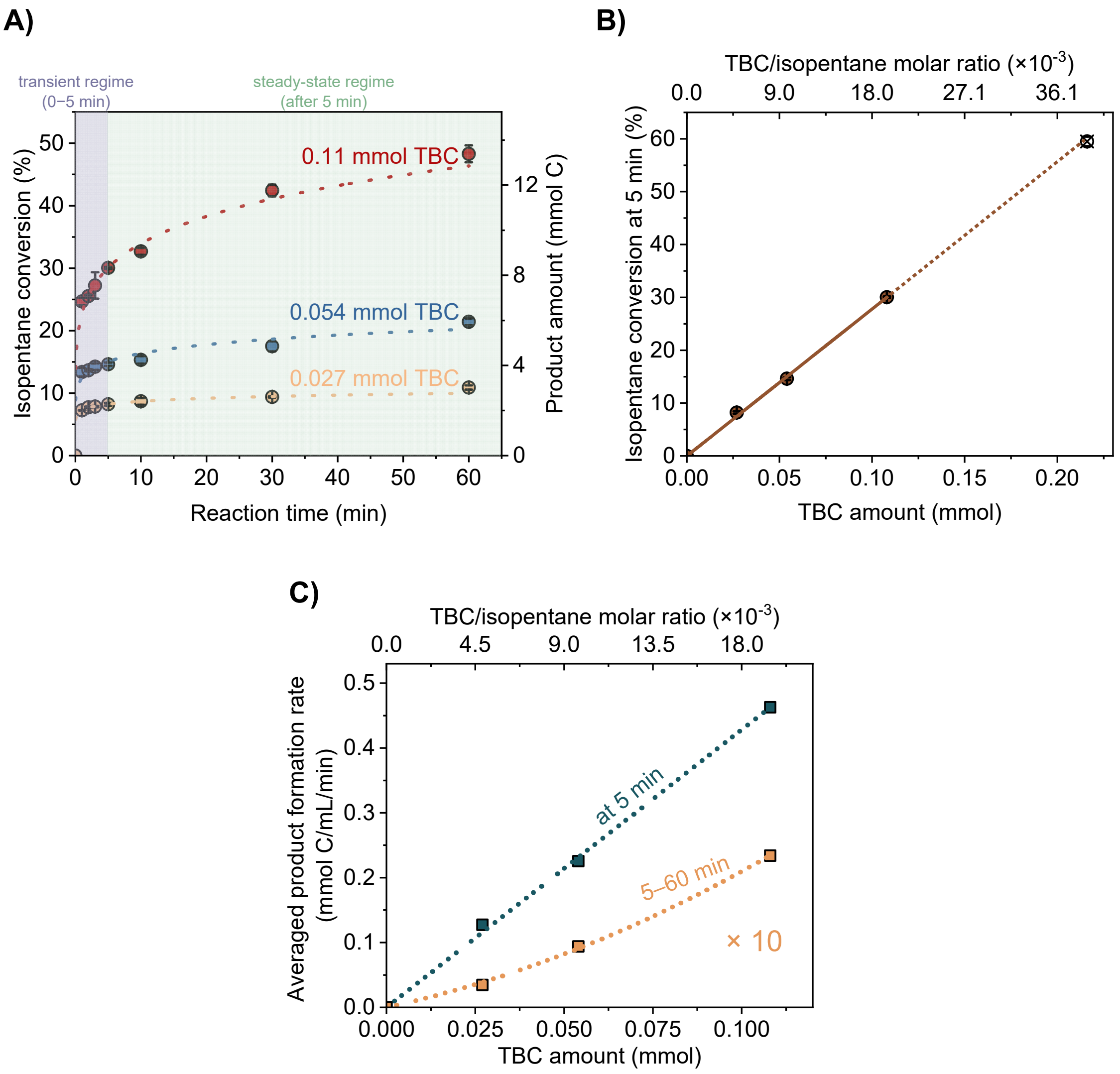

Figure S1. Detailed reaction kinetics of isopentane disproportionation reaction and its strong dependence on TBC amount. (**A**) Isopentane conversion (left axis) and the corresponding product amount in mmol C (total millimoles of carbon atoms in products, right axis, see Procedure for liquid phase product analysis of isopentane disproportionation for details) versus reaction time in the presence of different amounts of TBC. Reaction conditions were as follows: *i*C_5, 5.5 mmol (400 mg); ionic liquid, 1 mmol; TBC, 0.11 mmol (10 mg, red), 0.054 mmol (5 mg, blue), or 0.027 mmol (2.5 mg peach); DCM, 3 mL; and temperature, 25 °C. Curves are used to guide the eye. (**B**) Isopentane conversion at 5 min plotted against TBC amount. Same reaction conditions as in **Figure S1A**. The top axis displays the corresponding molar ratio of TBC to isopentane. The brown line is fitted to the data and extrapolated to 0.22 mmol (20 mg) TBC addition. (**C**) The averaged product formation rates for 0−5 min (teal) and 5−60 min (orange) plotted against the TBC amount. Same reaction conditions as in **Figure S1A**. Data for 5−60 min is scaled by a factor of ×10 for better visualization. The rates are not normalized to the

active species C^+-$AlCl_4^-$ to visually emphasize the correlation between the averaged product formation rates of the two regimes and the TBC amount.

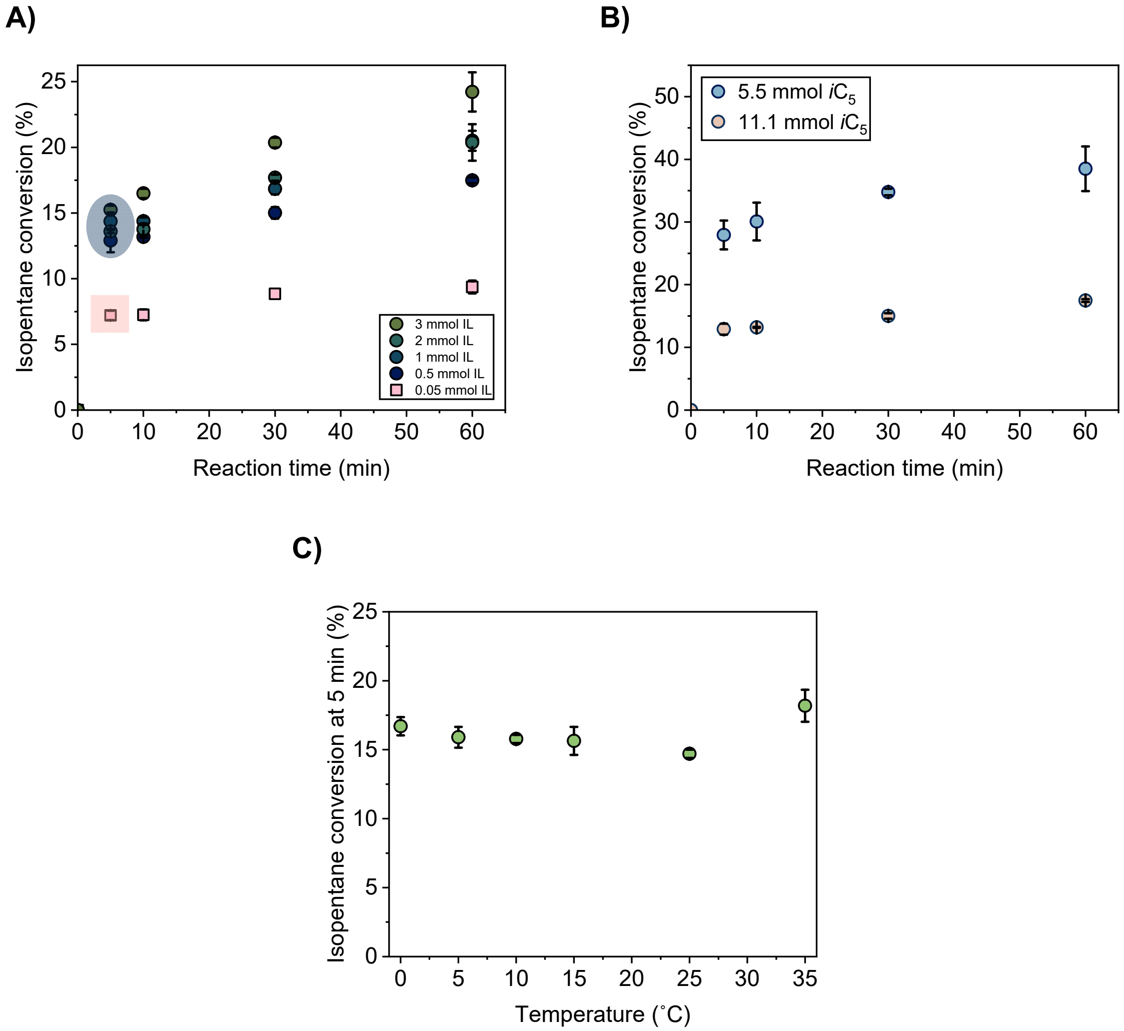

Figure S2. The influence of different reaction parameters on the transient regime (0−5 min) of isopentane disproportionation. (**A**) Isopentane conversion over time for isopentane disproportionation in the presence of different amounts of ionic liquid. Reaction conditions: *i*C_5, 11.1 mmol (800 mg); ionic liquid, 0.05−3 mmol; TBC, 0.11 mmol (10 mg); DCM, 3 mL; and temperature, 25 °C. (**B**) Isopentane conversion over time for isopentane disproportionation with different initial amounts of isopentane as reactant. Reaction conditions: *i*C_5, 5.5 or 11.1 mmol (400 or 800 mg); ionic liquid, 0.5 mmol; TBC, 0.11 mmol (10 mg); DCM, 3 mL; and temperature, 25 °C. (**C**) Isopentane conversion at 5 min plotted against temperatures varying from 0 °C to 35 °C. Reaction conditions: *i*C_5, 5.5 mmol (400 mg); ionic liquid, 1 mmol; TBC, 0.054 mmol (5 mg); DCM, 3 mL; and temperature, 0−35 °C.

The ionic liquid does not affect isopentane conversion in the transient regime unless its amount is insufficient to convert all added TBC into the initial carbenium ions. Our group has recently reported that carbenium ions are initiated by the interaction of a 1:1 molar ratio of ionic liquid and TBC.[16] For instance, with 0.11 mmol TBC, as shown in **Figure S2A**, when the ionic liquid amount is higher than the TBC amount, the transient regime results in the expected 15% isopentane conversion (blue-shaded area). If the ionic liquid amount is lower than the TBC amount (ionic liquid, 0.05 mmol; TBC, 0.11 mmol; pink-shaded area), the ionic liquid becomes the limiting factor for transient regime conversion, yielding 7.5% isopentane conversion. This observation indicates that the actual determining factor for the transient regime is the initial amount of carbenium ion. Since in our experiments, the amount of ionic liquid is always higher than the amount of TBC unless otherwise stated, the initial carbenium ion amount in the system can be considered equivalent to the amount of added TBC.

Figure S2B shows that the isopentane amount has minimal impact on its conversion in the transient regime. The absolute amount of converted isopentane remains nearly the same, and the halved conversion observed for 11.1 mmol compared to 5.5 mmol simply reflects the doubled reactant quantity. Doubling the reactant does not increase the conversion rate; instead, it slightly lowers it. This is because an excess of *i*C5 dilutes the carbenium ion concentration in the system, thereby reducing the reaction rate. The same observation was reported in our LDPE tandem cracking-alkylation experiments.[1] This finding aligns well with our hypothesis that carbenium ion concentration determines the reaction rate.

Figure S2C shows the temperature invariance for the isopentane conversion during the transient regime. In this series of experiments with 0.054 mmol TBC additive, the disproportionation product amount remained constant at 60 mg across reaction temperatures from 0 to 35 °C (plotted as isopentane conversion, which remained constant at 15%).

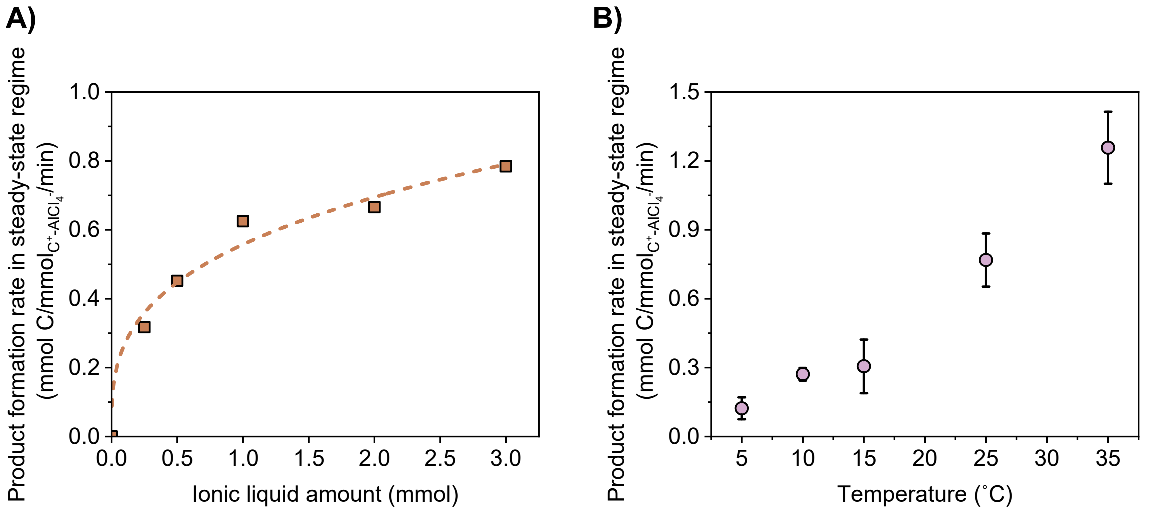

Figure S3. The influence of different reaction parameters on the steady-state regime (5−60 min) of isopentane disproportionation. (**A**) Product formation rates in the steady-state regime plotted against ionic liquid amounts varying from 0.25 mmol to 3 mmol. Reaction conditions: iC_5, 11.1 mmol (800 mg); ionic liquid, 0.05−3 mmol; TBC, 0.11 mmol (10 mg); DCM, 3 mL; and temperature, 25 °C. Curve is used to guide the eye. (**B**) Product formation rates in the steady-state regime plotted against temperatures varying from 5 °C to 35 °C, as a qualitative illustration of the effect of temperature on the steady-state regime. Increasing the temperature clearly affects this reaction regime, in contrast to the transient regime, for which temperature has no significant impact (**Figure S2C**). Reaction conditions: iC_5, 5.5 mmol (400 mg); ionic liquid, 1 mmol; TBC, 0.054 mmol (5 mg); DCM, 3 mL; and temperature, 5−35 °C.

Figure S3A shows that the product formation rate in the steady-state regime increases with increasing ionic liquid amount. However, when the ionic liquid amount exceeds 1 mmol, its effectiveness in increasing the reaction rate becomes less significant. Due to its high viscosity, excessive amount of the ionic liquid leads to phase separation between the ionic liquid and isopentane, thereby impairing mass transfer. The same observation was reported in our LDPE tandem cracking-alkylation experiments.[1] Therefore, all experiments were conducted with ionic liquid amounts no greater than 1 mmol to avoid diffusion limitations.

Figure S3B shows that the product formation rate in the steady-state regime increases with increasing temperature. An activation energy for the steady-state regime can be obtained (**Figure 6** in the main text).

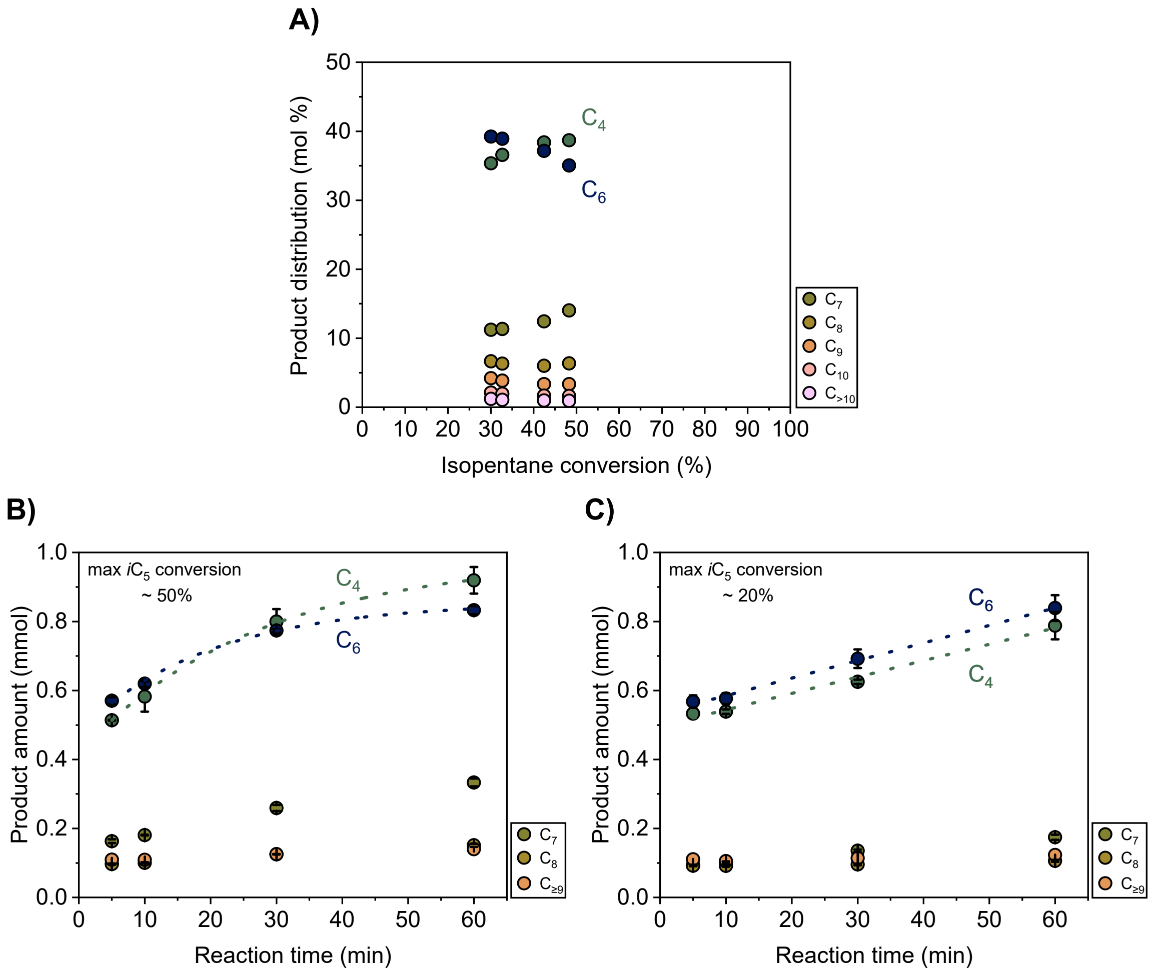

Figure S4. Slight shift in product distribution of isopentane disproportionation at higher isopentane conversion (ca. 40%) due to secondary disproportionation reaction. (**A**) Product distribution of isopentane disproportionation plotted against isopentane conversion. Reaction conditions: iC_5, 5.5 mmol (400 mg); ionic liquid, 1 mmol; TBC, 0.11 mmol (10 mg); DCM, 3 mL; and temperature, 25 °C. (**B**) Product amounts of different alkanes plotted against reaction time. Same reaction condition as **Figure S4A**. Maximum isopentane conversion ca. 50%. Alkane products with carbon number ≥ 9 are summed due to their small amounts. Curves are used to guide the eye. (**C**) Product amounts of different alkanes plotted against reaction time. Reaction conditions: iC_5, 11.1 mmol (800 mg); ionic liquid, 1 mmol; TBC, 0.11 mmol (10 mg); DCM, 3 mL; and temperature, 25 °C. Maximum isopentane conversion ca. 20%. Alkane products with carbon number ≥ 9 are summed due to their small amounts. Curves are used to guide the eye.

A decrease in the percentage of C_6, accompanied by a corresponding increase in the percentages of C_4 and C_7, is observed as the isopentane conversion approaches 40% (**Figure S4A**). This trend indicates a secondary disproportionation reaction between C_5 and C_6, leading to the formation of C_4 and C_7. As the reaction progresses, the reduction in C_5 concentration and the increase in C_6 concentration increases the likelihood of the disproportionation reaction between C_5 and C_6

molecules. Note that although C_4 is produced as a main product, the secondary disproportionation between C_4 and C_5 molecules yielding C_3 and C_6 does not occur. This is because the β-scissions that yield C_3 fragments involve secondary carbenium ions, and these types of β-scissions are known to be unfavorable.[17] The C_9 carbenium ion formed from the alkylation of C_4 and C_5 is more prone to crack back into C_4 and C_5 fragments rather than forming C_3 and C_6 fragments.

A comparison of **Figure S4B** with **Figure S4C** illustrates the effect of secondary disproportionation between C_5 and C_6 on product distribution. In both experiments, the reaction conditions were identical except that in **Figure S4C**, the amount of isopentane was increased from 5.5 mmol to 11.1 mmol, ensuring an excess of isopentane. In this scenario, the amounts of C_4 and C_6 increase almost linearly, indicating that the reaction remains within the kinetic regime. Additionally, the formation of C_7 is suppressed.

It is important to note that approximately 50% isopentane conversion represents the upper limit in our experiments. Unless specified otherwise, all other results were obtained with isopentane conversions below 20%. At these lower conversions, isopentane disproportionation is the predominant reaction. Side reactions are relatively minor.

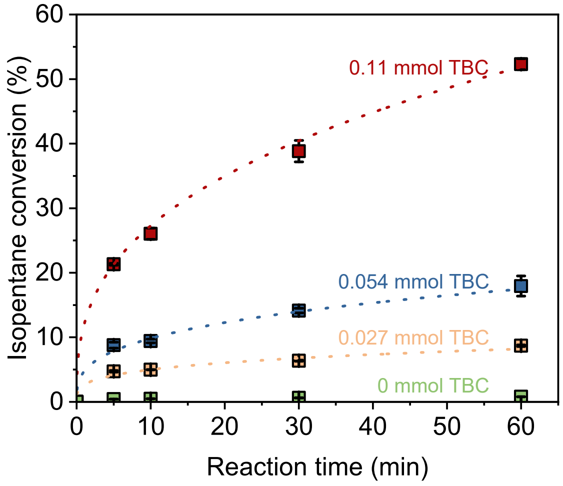

Figure S5. Isopentane disproportionation catalyzed by anhydrous $AlCl_3$. Isopentane conversion over time in the presence of different amounts of TBC additive. Reaction conditions: *i*C_5, 11.1 mmol (800 mg); anhydrous $AlCl_3$, 0.375 mmol (50 mg); TBC, 0−0.11 mmol (0−10 mg); DCM, 3 mL; and temperature, 25 °C.

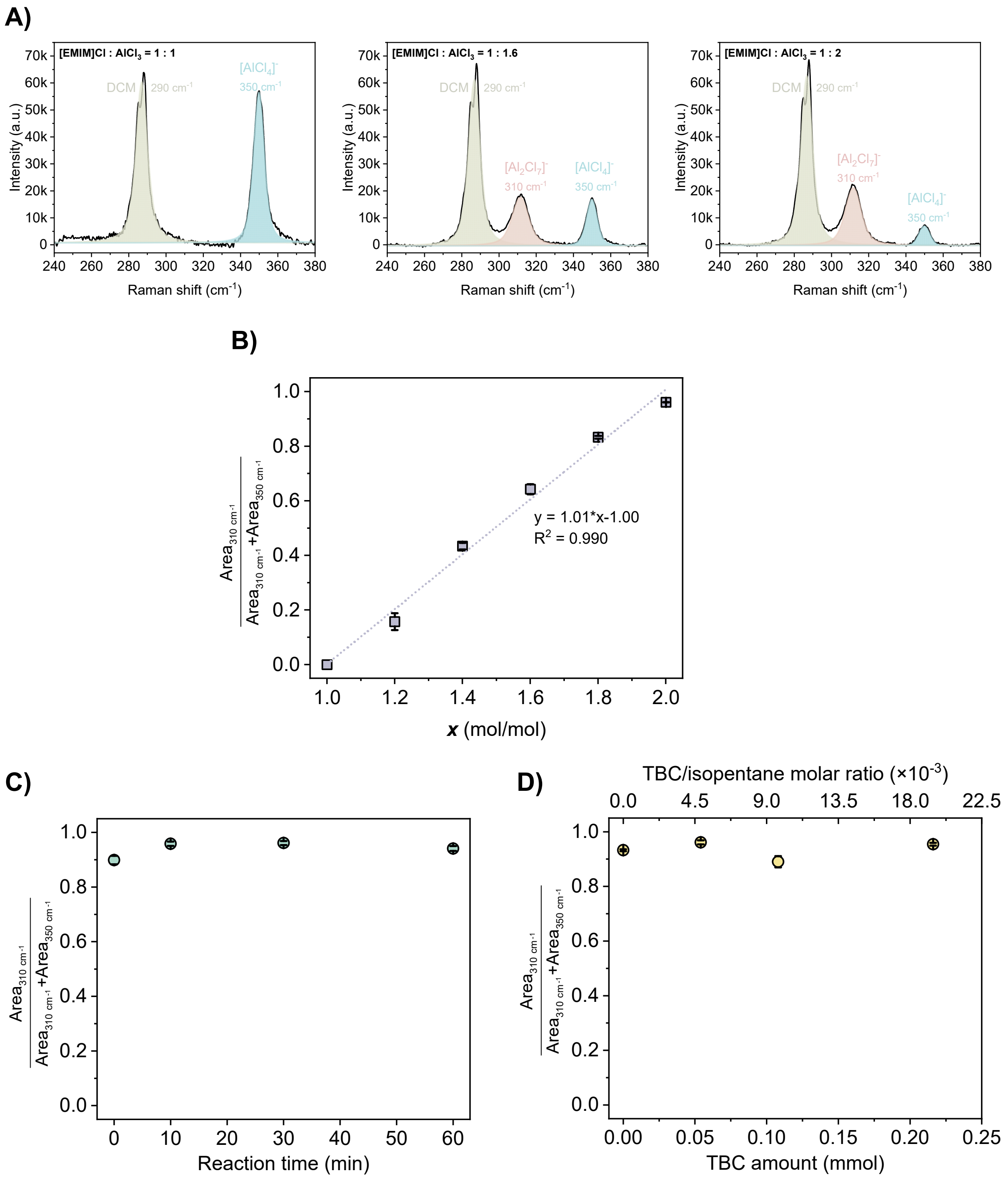

Figure S6. Characterizing chloroaluminate species in the ionic liquid by Raman spectroscopy. (**A**) Examples of measured Raman spectra, showing Raman signals at 290 cm^{-1} (DCM), 310 cm^{-1} ($Al_2Cl_7^-$), and 350 cm^{-1} ($AlCl_4^-$). Experimental data drawn in black, fitted using the Voigt function. Deconvoluted peaks in green (290 cm^{-1}), red (310 cm^{-1}), and blue (350 cm^{-1}). Shown samples were prepared using ionic liquid [EMIM]Cl-$x$$AlCl_3$ with different molar ratios of $AlCl_3$: x = 1 (left), 1.6 (middle), and 2 (right). (**B**) Plot of area ratios of $Al_2Cl_7^-$ anion ($Al_2Cl_7^-$/[$Al_2Cl_7^-$+$AlCl_4^-$]) versus the molar ratios of $AlCl_3$/[EMIM]Cl (x). (**C**) Raman area

ratios of $Al_2Cl_7^-$ at different reaction times, TBC amount fixed at 0.054 mmol. Reaction conditions: [EMIM]Cl-$x$$AlCl_3$ (x=2.0) ionic liquid, 2 mmol; iC_5, 11.1 mmol (800 mg); DCM, 3 mL; reaction time, 0−60 minutes; and temperature, 25 °C. (**D**) Raman area ratios of $Al_2Cl_7^-$ with different TBC additive amounts. The top axis displays the corresponding molar ratio of TBC to isopentane. Ionic liquid sampled after 30 minutes of reaction. Reaction conditions: [EMIM]Cl-$x$$AlCl_3$ (x=2) ionic liquid, 2 mmol; iC_5, 11.1 mmol (800 mg); DCM, 3 mL; TBC amount, 0−0.22 mmol; and temperature, 25 °C.

Raman spectroscopy has been widely applied to characterize and quantify chloroaluminate anions in ionic liquids.[18-21] To establish an external calibration curve, we measured a series of [EMIM]Cl-$x$$AlCl_3$ ionic liquids (x from 1.0 to 2.0), with the gradually increasing $Al_2Cl_7^-$ content and the correspondingly decreasing $AlCl_4^-$ content. **Figure S6A** shows the Raman spectra of the samples prepared using ionic liquid [EMIM]Cl-$x$$AlCl_3$ with different molar ratios of $AlCl_3$. Note that the ionic liquids with x<2 were used only for reference purposes and were not used to catalyze the isopentane disproportionation reaction. To accurately examine the chloroaluminate species under our reaction conditions, all samples were prepared by mixing 2 mmol [EMIM]Cl-$x$$AlCl_3$ (x=1.0−2.0) ionic liquid, 11.1 mmol iC_5, and 3 mL DCM at 25 °C, with the ionic liquids sampled after phase separation. Note that no TBC was added to the mixture; thus, the isopentane remained unconverted. Isopentane was included solely to replicate the actual reaction conditions. The obtained Raman spectra (**Figure S6A**) display three peaks. The peak at 290 cm^{-1} corresponds to the Cl−C−Cl scissoring mode from DCM.[22,23] The peak at 310 cm^{-1} is attributed to the symmetric Al−Cl−Al stretching mode of the dimeric $Al_2Cl_7^-$ anion,[24,25] while the peak at 350 cm^{-1} is assigned to the symmetric Cl−Al−Cl stretch of the monomeric $AlCl_4^-$ anion.[26] The deconvoluted peak area ratios of the $Al_2Cl_7^-$ anion plotted against the molar ratios of $AlCl_3$/[EMIM]Cl (x) shows a linear correlation (**Figure S6B**), which aligns closely with our previously reported data. This indicates that Raman spectroscopy accurately quantifies chloroaluminate anions and is not influenced by our reaction conditions.

Figure S6C and **S6D** show that the area ratio of the $Al_2Cl_7^-$ anion ($Al_2Cl_7^-$/[$Al_2Cl_7^-$+$AlCl_4^-$]) remains constant across different reaction times and amounts of TBC. This observation implies that the molar ratio of $Al_2Cl_7^-$ to $AlCl_4^-$ is stable. According to our previously reported dissociation equilibrium between the dimeric $Al_2Cl_7^-$ and the monomeric $AlCl_4^-$ and $AlCl_3$* ($Al_2Cl_7^- \rightleftharpoons AlCl_4^- + AlCl_3$*), the amounts of these three Al species are interrelated. Consequently, the result further indicates that the amount of the active chloroaluminate species $AlCl_3$* remains unchanged throughout the reaction and is not influenced by the amount of TBC added.

Although it is anticipated that the $AlCl_3$* amount remains constant throughout the reaction, as the catalyst should not be consumed, it is surprising that the TBC addition does not affect the chloroaluminate species. We propose that TBC interacts readily with $AlCl_3$*, forming active carbenium ion-$AlCl_4^-$ ion-pairs. The formation of ion-pairs consumes $AlCl_3$*, leading to the

production of $AlCl_4^-$ and a change in the molar ratio of $Al_2Cl_7^-$ to $AlCl_4^-$. The observed constant ratio suggests that the addition of TBC either does not affect the dissociation equilibrium $Al_2Cl_7^- \rightleftharpoons AlCl_4^- + AlCl_3$*, or the amount added is too small to induce a significant difference.

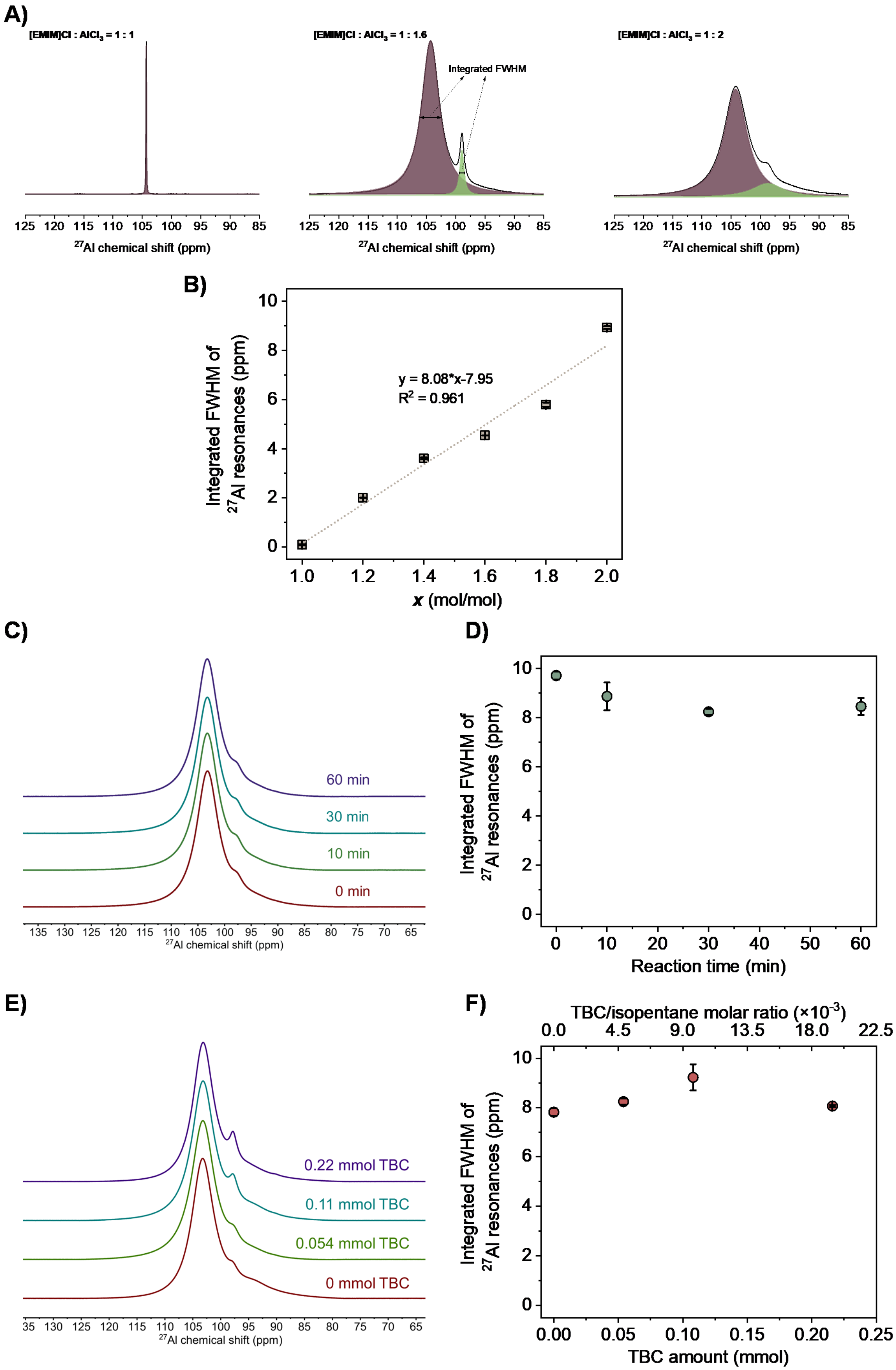

A)
[EMIM]Cl : AlCl$_3$ = 1 : 1
[EMIM]Cl : AlCl$_3$ = 1 : 1.6
[EMIM]Cl : AlCl$_3$ = 1 : 2
Integrated FWHM
^{27}Al chemical shift (ppm)
B)
y = 8.08*x-7.95
R^2 = 0.961
Integrated FWHM of ^{27}Al resonances (ppm)
x (mol/mol)
C)
60 min
30 min
10 min
0 min
^{27}Al chemical shift (ppm)
D)
Integrated FWHM of ^{27}Al resonances (ppm)
Reaction time (min)
E)
0.22 mmol TBC
0.11 mmol TBC
0.054 mmol TBC
0 mmol TBC
^{27}Al chemical shift (ppm)
F)
TBC/isopentane molar ratio (×10^{-3})
Integrated FWHM of ^{27}Al resonances (ppm)
TBC amount (mmol)

Figure S7. Characterizing chloroaluminate species in the ionic liquid by ^{27}Al NMR spectroscopy. (**A**) Examples of recorded ^{27}Al NMR spectra, showing ^{27}Al resonances at 103 ppm and 98 ppm. Experimental data drawn in black, fitted using the Lorentzian function. Deconvoluted peaks in purple (103 ppm) and green (98 ppm). Shown samples were prepared using ionic liquid [EMIM]Cl-xAlCl$_3$ with different molar ratios of AlCl$_3$: x = 1 (left), 1.6 (middle), and 2 (right). (**B**) Plot of integrated FWHM of ^{27}Al resonances versus the molar ratios of AlCl$_3$/[EMIM]Cl (x). (**C**) ^{27}Al NMR spectra of [EMIM]Cl-xAlCl$_3$ (x=2) ionic liquids sampled at different reaction times. (**D**) Integrated FWHM of ^{27}Al resonances at different reaction times. Reaction conditions: [EMIM]Cl-xAlCl$_3$ (x=2.0) ionic liquid, 2 mmol; iC$_5$, 11.1 mmol (800 mg); DCM, 3 mL; reaction time, 0−60 minutes; and temperature, 25 °C. TBC amount fixed at 0.054 mmol. (**E**) ^{27}Al NMR spectra of [EMIM]Cl-xAlCl$_3$ (x=2) ionic liquids collected from reactions with different TBC amounts. (**F**) Integrated FWHM of ^{27}Al resonances at different TBC amounts. Reaction conditions: [EMIM]Cl-xAlCl$_3$ (x=2) ionic liquid, 2 mmol; iC$_5$, 11.1 mmol (800 mg); DCM, 3 mL; TBC amount, 0−0.22 mmol; and temperature, 25 °C. The top axis displays the corresponding molar ratio of TBC to isopentane. Ionic liquid sampled after 30 minutes of reaction.

We performed ^{27}Al NMR spectroscopy to supplement the Raman spectroscopy for characterizing chloroaluminate species. The samples were prepared using the same method described in the Raman spectroscopy section to replicate the actual reaction conditions. In a typical ^{27}Al NMR spectrum, two peaks are observed at 103 and 98 ppm, which are attributed to $AlCl_4^-$ and $Al_2Cl_7^-$, respectively.[27,28] **Figure S7A** shows the ^{27}Al spectra of the samples prepared using ionic liquid [EMIM]Cl-xAlCl$_3$ with different molar ratios of AlCl$_3$. It illustrates how the ^{27}Al peaks evolve as the molar ratio of AlCl$_3$ in the ionic liquid varies. However, the intensities of these signals could not be used directly for quantification, as they were not directly correlated to the concentrations of the two chloroaluminate anions. Instead, we employed a new method to characterize chloroaluminate species by analyzing the linewidths of these resonances. We introduced the integrated full width at half maximum (FWHM), defined as the sum of the FWHMs of the deconvoluted peaks at 103 ppm and 98 ppm (**Figure S7A**). We hypothesize that the linewidths of the two resonances at 103 ppm and 98 ppm broaden due to a dynamic interconversion between $Al_2Cl_7^-$ and $AlCl_4^-$ ($Al_2Cl_7^- + AlCl_4^- \rightarrow AlCl_4^- + Al_2Cl_7^-$).[29] As the concentration of $Al_2Cl_7^-$ in the system increases, the frequency of this interconversion also increases, leading to broader signals. **Figure S7B** shows the linear correlation between the integrated FWHM and the molar ratio of AlCl$_3$/[EMIM]Cl (x). It is important to note that FWHM comparisons are valid only at the same temperature, as increasing temperature accelerates molecular motions, intensifies interconversion, and narrows the FWHM.[29] All spectra were collected at 25 °C.

Figure S7C presents the ^{27}Al NMR spectra of the ionic liquids sampled at different reaction times, showing no significant changes across the spectra. Consequently, the integrated FWHM

of the two peaks (103 and 98 ppm) remains nearly constant with reaction time, indicating that the concentration of $Al_2Cl_7^-$ is stable throughout the reaction. This result is consistent with the findings from the Raman spectroscopy. **Figure S7E** displays the ^{27}Al NMR spectra of ionic liquids sampled from reactions with varying amounts of TBC additive. As the amount of TBC increases, the 98 ppm peak becomes slightly more distinct. However, this change has a minimal impact on the integrated FWHM, as the predominant peak at 103 ppm remains unchanged. Therefore, the integrated FWHM remains largely stable, indicating that the $Al_2Cl_7^-$ anion concentration is not affected by the TBC concentration either, aligning with our Raman spectroscopy results.

The ^{27}Al NMR spectroscopy results corroborate the Raman spectroscopy findings, indicating that the concentration of the active chloroaluminate species remains stable throughout the reaction and is unaffected by the addition of TBC.

These results suggest that the Lewis acidity of our ionic liquid, which is mainly determined by the $Al_2Cl_7^-$ concentration,[30] remains unaffected by the reaction time and the TBC amounts tested. While the reaction with the added TBC may cause a slight reduction in the Lewis acidity of the ionic liquid, this cannot explain the regime transition. Our results (**Figure S1C**) show that the highest concentration of TBC corresponds to the highest steady-state reaction rate, contrary to expectations based on ionic liquid neutralization and reduced Lewis acidity. This observation cannot be reconciled by arguing for the formation of more active species, as that scenario would also imply the most significant rate impairment during the regime transition, which we do not observe. Therefore, we conclude that a change in Lewis acidity is not responsible for the occurrence of the two kinetic regimes.

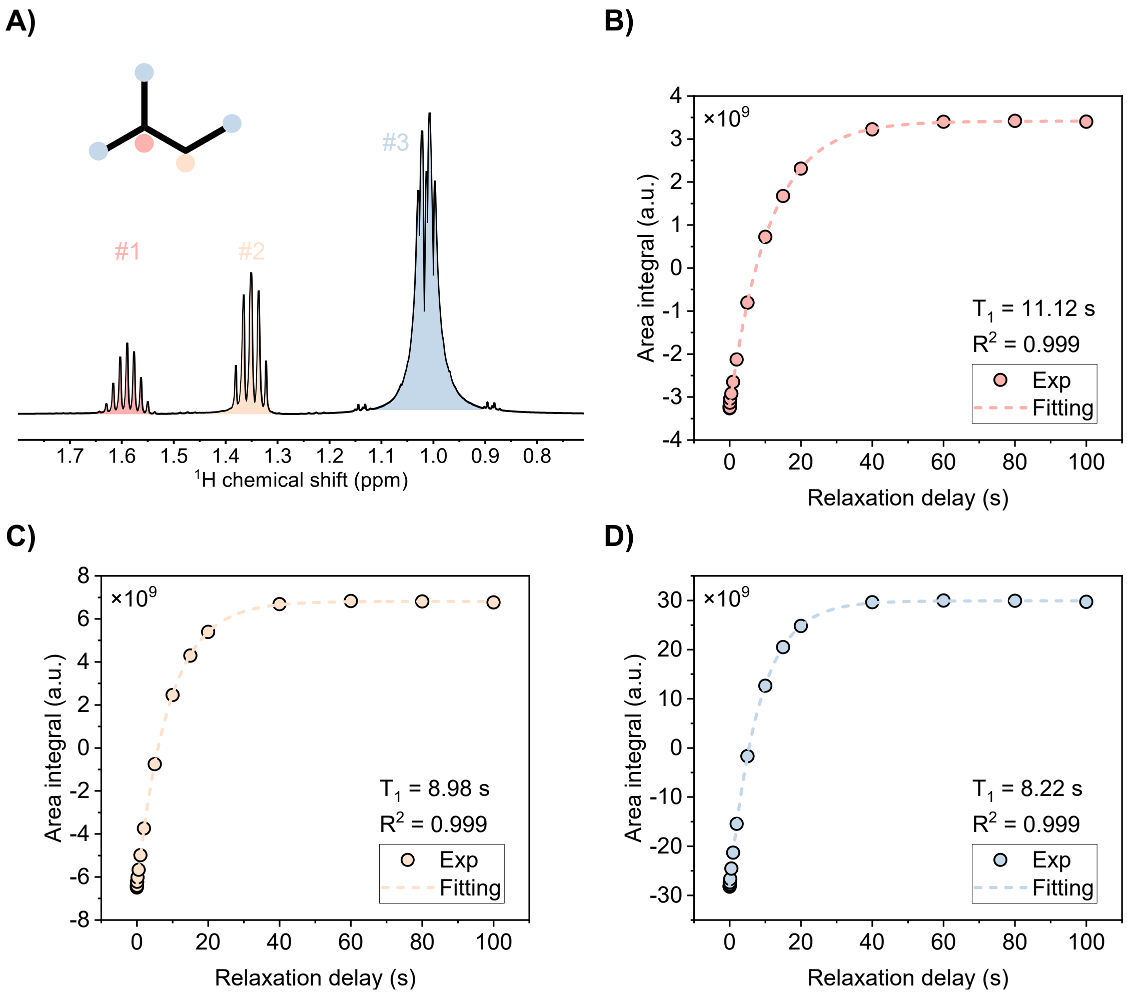

Figure S8. ^{1}H NMR T_1 relaxation time constants determination for isopentane at 25 °C. T_1 was determined separately for each peak. The assignment of each peak (numbered) and its T_1 data analysis plot is indicated with a designated color.

^{1}H NMR T_1 values of the selected components in our reaction system (isopentane, reactant; 2-methylpentane, product; 2-methyl-2-butene and 2-methyl-2-pentene, alkene; benzene, internal standard) range from 5.92 s to 12.09 s. The interpulse delay list (unit: second) was set to 0.01, 0.02, 0.05, 0.1, 0.2, 0.5, 1, 2, 5, 10, 15, 20, 40, 60, 80, 100; to ensure sufficient data points for capturing the entire relaxation curves for the T_1 range. The area integrals were directly measured using TopSpin and the fittings were done using the equation $M=M_0*(1-2*A*\exp(-\tau/T_1))$. All T_1 relaxation time constant determinations presented in this work followed the same method described above.

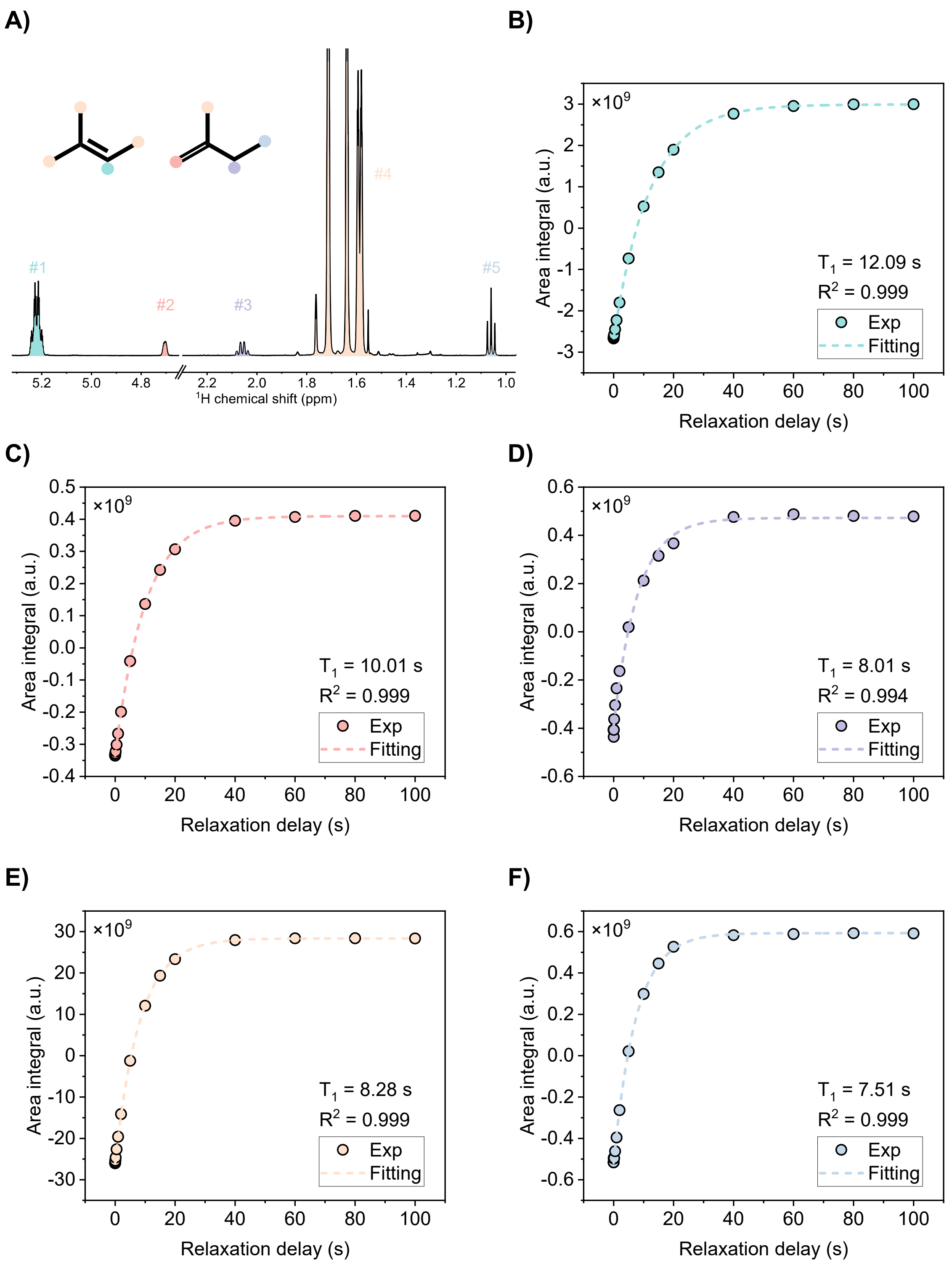

Figure S9. ^{1}H NMR T_1 relaxation time constants determination for 2-methyl-2-butene (containing 2-methyl-1-butene as impurity) at 25 °C. T_1 was determined separately for each peak. The assignment of each peak (numbered) and its T_1 data analysis plot is indicated with a designated color.

2-Methyl-2-butene was selected to represent the alkenes formed during the reaction. 2-Methyl-1-butene, which was present as an impurity in the chemical, was also included in the T_1 determination. Peaks in the region of 1.5–1.8 ppm (allylic protons) could not be well separated and, therefore, were integrated together for the T_1 determination.

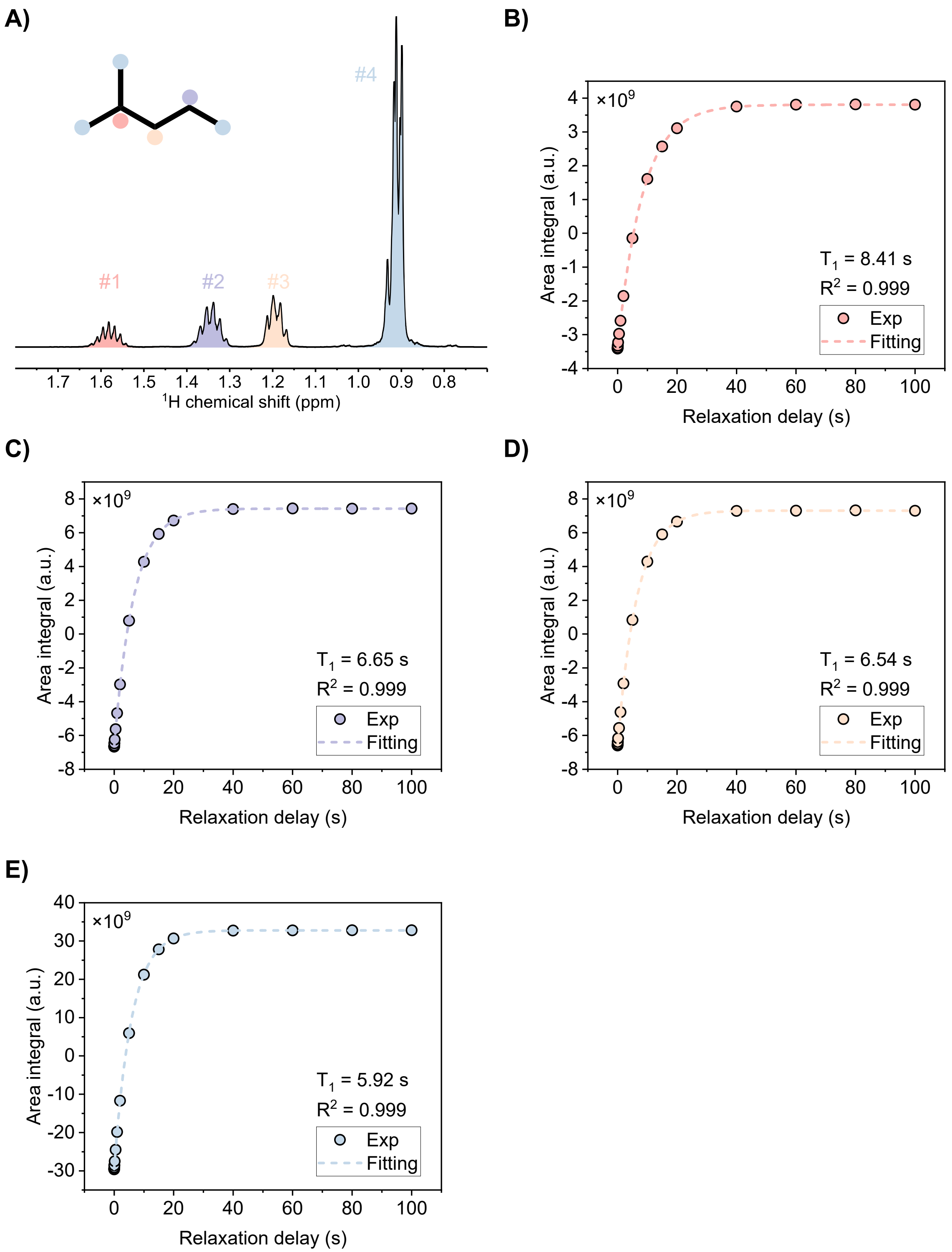

Figure S10. ^{1}H NMR T_1 relaxation time constants determination for 2-methylpentane at 25 °C. T_1 was determined separately for each peak. The assignment of each peak (numbered) and its T_1 data analysis plot is indicated with a designated color. 2-Methylpentane was chosen as the representative of the products.

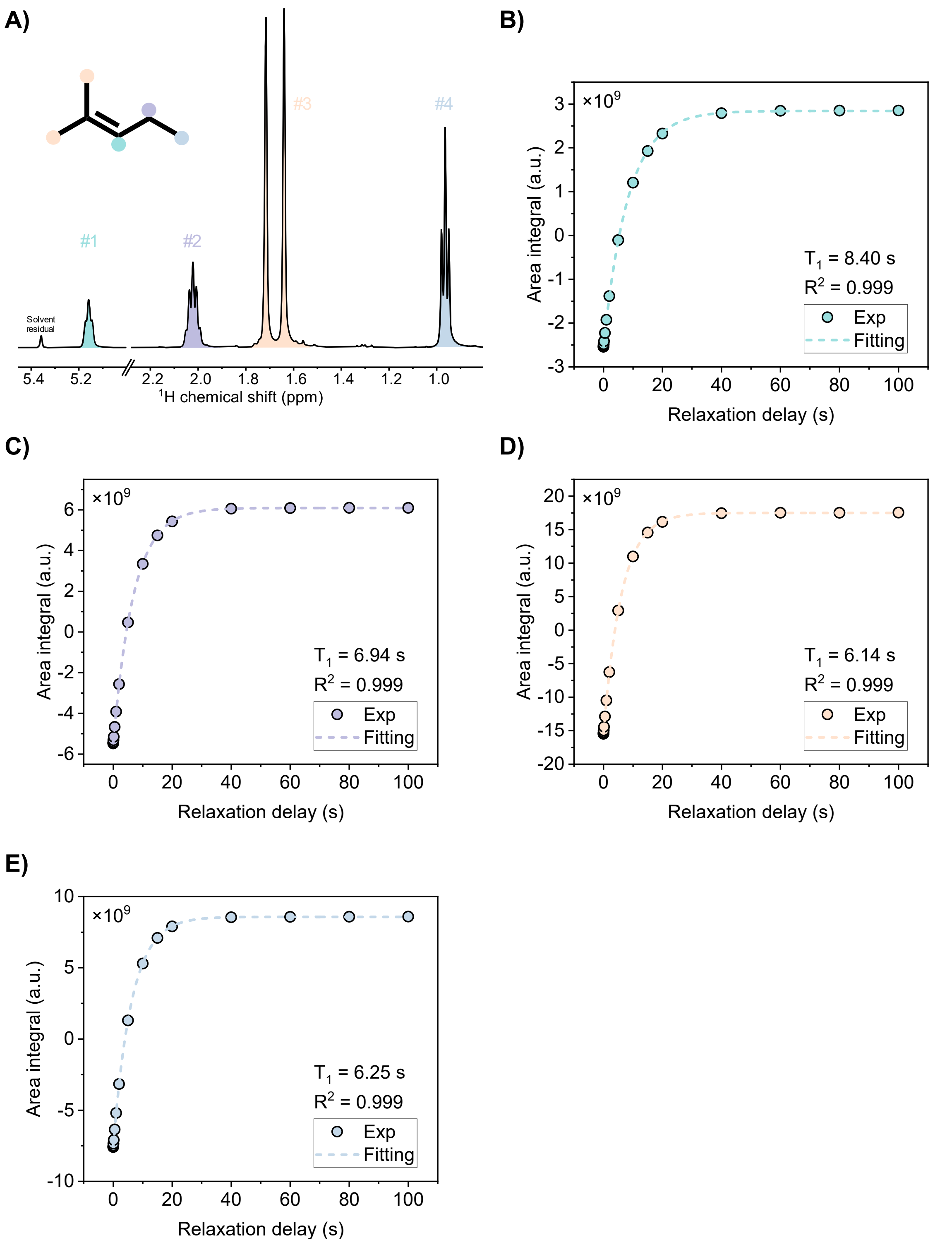

Figure S11. ^{1}H NMR T_1 relaxation time constants determination for 2-methyl-2-pentene at 25 °C. T_1 was determined separately for each peak. The assignment of each peak (numbered) and its T_1 data analysis plot is indicated with a designated color. 2-Methyl-2-pentene was chosen as the representative of the alkene intermediates.

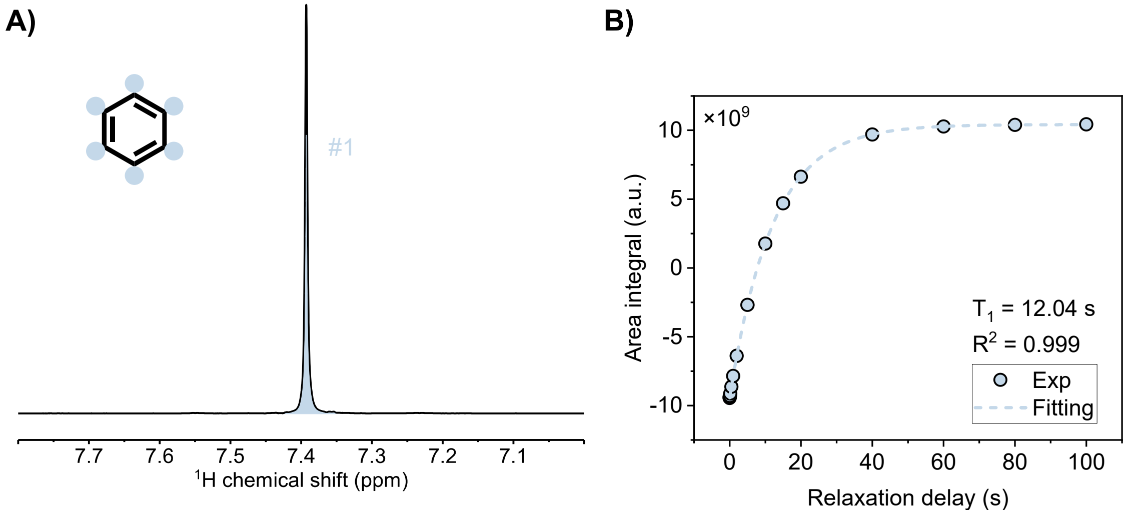

Figure S12. ^{1}H NMR T_1 relaxation time constant determination for benzene at 25 °C.

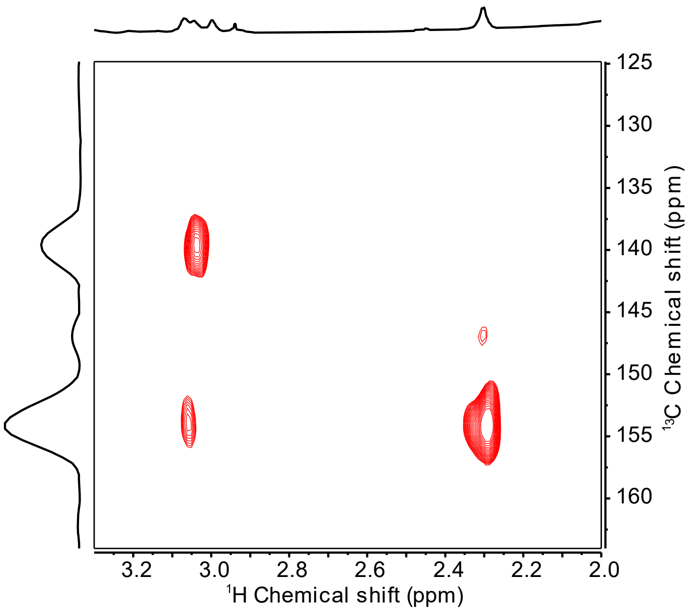

Figure S13. ^{1}H–^{13}C 2D HMBC spectrum of isopentane disproportionation in the presence of TBC additive at 25 °C. Reaction conditions: $AlCl_3$-saturated dichloromethane-d_2, 0.6 mL; $i$$C_5$, 1.1 mmol (80 mg); TBC, 43.2 μmol (4 mg); and temperature, 25 °C.

2D ^{1}H–^{13}C HSQC experiment was also conducted. However, due to the very low concentration of the alkene species, no cross peak in the alkene region was observed.

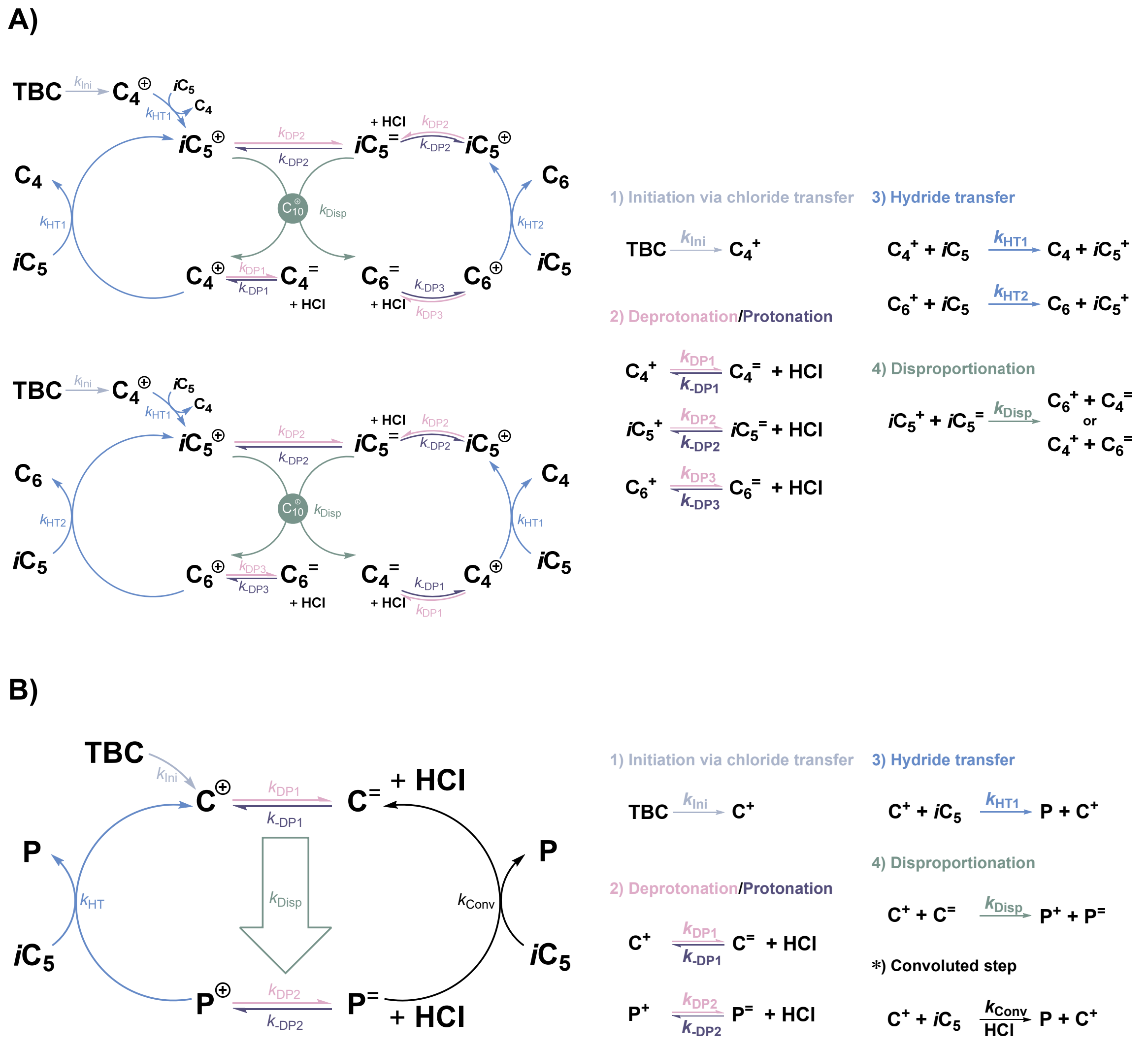

Figure S14. Complex and intermediate kinetic models constructed in COPASI. (**A**) The complex model, showing the reaction networks for the C_6-focused (top left, forming off-cycle C_6 alkene reservoir) and C_4-focused (bottom left, forming off-cycle C_4 alkene reservoir) variants, alongside their shared elementary steps. C_n^+ in the reaction network always represents the corresponding C_n^+-$AlCl_4^-$ ion-pair, and HCl the HCl-chloroaluminate species. (**B**) Reaction network of the intermediate kinetic model (left), and the corresponding elementary steps (right). C^+ and P^+ in the reaction network represent C^+-$AlCl_4^-$ and P^+-$AlCl_4^-$ ion-pairs, respectively. HCl represents the HCl-chloroaluminate species.

To provide a robust mechanistic interpretation of the experimental data, a series of kinetic models with varying complexity were developed and analyzed using COPASI.

The most detailed representation of the proposed reaction mechanism (**Figure 3**) is the complex model (**Figure S14A**). It incorporates three deprotonation/protonation equilibria for C_4, C_5, and C_6, and includes two distinct hydride transfer steps for C_4 and C_6. The major simplification is combining the alkene addition, isomerization, and cracking into a single step, disproportionation. As the disproportionation step can produce either C_4^+ and $C_6^=$ or $C_4^=$ and C_6^+, we differentiate two model variants: a C_4-focused one, which accumulates $C_4^=$ as the off-cycle alkene reservoir, and a C_6-focused one, which accumulates $C_6^=$. It should be noted in passing that although each cracking event can only yield one set of products, either C_4^+ and $C_6^=$ or $C_4^=$ and C_6^+, there is no indication that one product set is favored over the other. Consequently, the actual mechanism is likely a combination of the two variants.

Analysis of the complex model reveals a key insight: only three rate constants govern the overall kinetics, analogously to those of the core model discussed in the main text. More importantly, these constants are all related to a single carbenium ion species that participates in the off-cycle equilibrium. For the C_4-focused variant, the influential rate constants—k_{HT1}, k_{DP1}, and k_{-DP1}—are all associated with C_4^+, which establishes the off-cycle equilibrium with $C_4^=$. Similarly, for the C_6-focused variant, the influential rate constants—k_{HT2}, k_{DP3}, and k_{-DP3}—are all related to C_6^+. The carbenium becomes kinetically decisive when its deprotonation equilibrium is not integrated into the reaction network. The carbenium ion exits the reaction in the form of an alkene, and the only way for the alkene to rejoin the reaction network is through protonation. This is the essence of the off-cycle concept.

The similarity between the two complex model variants prompts further simplifications. In the intermediate model (**Figure S14B**), we no longer distinguish between C_4 and C_6 species but instead lump them into a generic “product” category (P). The iC_5 carbenium ions, originally formed via chloride abstraction from TBC and hydride transfer from isopentane, are now provided to the system through a single initiation step. In the disproportionation step, iC_5 carbenium ion (C^+) reacts with iC_5 alkene ($C^=$) to yield product carbenium ion (P^+) and product alkene ($P^=$). The final products are then formed from P^+ and $P^=$ in two distinct catalytic cycles, as depicted in **Figure S14B**. The left cycle is the hydride transfer, which converts P^+ into a product while regenerating C^+ for the subsequent cycle. The right cycle is the regeneration of $C^=$, accompanied by product formation. This should be a multi-step process involving protonation, hydride transfer, and deprotonation. For simplification, these steps are combined into a single convoluted step in this model. Note that because C_4 and C_6 are not distinguished, an artificial equilibrium between P^+ and $P^=$ exists, which does not reflect the actual mechanism.

Kinetic analysis of the intermediate model yields a conclusion similar to that of the complex model: hydride transfer and deprotonation/protonation remain the critical, rate-governing steps, while the disproportionation and initiation have a negligible effect on the reaction rate or kinetic behavior. Despite correctly identifying these influential elementary steps, the intermediate model

fails to accurately represent the off-cycle equilibrium concept, as the $P^+ \rightleftharpoons P^=$ equilibrium is integrated into the network.

To provide a more straightforward and general intuition for the underlying kinetic behavior, and to establish a robust and mathematically identifiable framework for using this reaction to evaluate the hydride transfer ability of catalysts, we distill the mechanism into a core model based on the off-cycle equilibrium concept, as discussed in the following section.

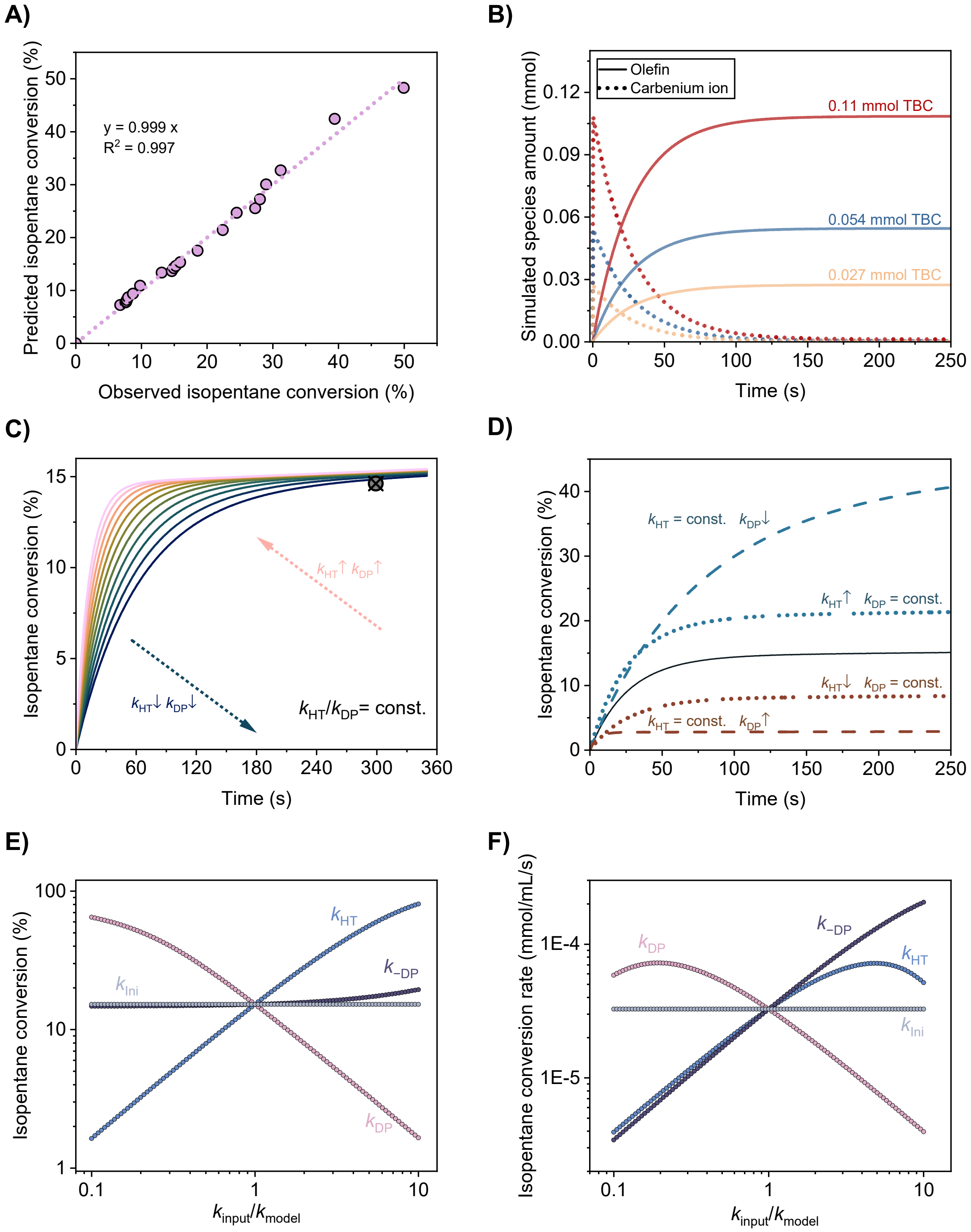

Figure S15. Detailed kinetic analysis of the core model in COPASI. (**A**) Parity plot of experimental vs. kinetic model simulated isopentane conversion. (**B**) COPASI-simulated alkene and carbenium ion concentrations during the reaction with varying amounts of TBC. (**C**) Fixed ratio between k_{HT} and k_{DP} required to fit the experimental data point at 300 s. (**D**) Effects of k_{HT} and k_{DP} on the simulated isopentane conversion curve in the transient regime. (**E**) Sensitivity

analysis results for the transient regime (<300 s) based on the core kinetic model. (**F**) Sensitivity analysis results for the steady-state regime (>300 s) based on the core kinetic model. k_{model} is the rate constant optimized to fit the experimental data, set as the default value in the core model (see **Table S1**). k_{input} is the manually adjusted value of the corresponding rate constants (from 0.1× to 10× k_{model}) used for the sensitivity analysis.

Figure 5A depicts the most distilled model we developed in COPASI, incorporating several simplifications: (1) The carbenium ion-$AlCl_4^-$ ion-pairs are symbolized as carbenium ions. (2) Carbenium ions function solely as catalytic chain carriers. (3) Alkenes act solely as off-cycle species that decrease the system's carbenium ion concentration rather than participating in the reaction. (4) C_4 and C_6 alkanes are not distinguished and are collectively referred to as "product." (5) Isopentane is directly converted into "product" in a single step. Although the simplicity of the model cannot fully account for the chemical complexity of the system, by leveraging the off-cycle equilibrium concept, it effectively fits the experimental data with only four kinetic parameters, as shown in **Figure 5B** and **Figure S15A**. The model accurately reflects the sharp increase in carbenium ion concentration, proportional to the amount of added TBC, due to the rapid interaction between TBC and $AlCl_3$*. Subsequently, the off-cycle equilibrium causes carbenium ions to continuously transform into alkenes, the quantity of which is also proportional to the TBC amount (**Figure S15B**). In the steady-state regime, alkenes and carbenium ions reach equilibrium, and their concentrations remain constant. Because the carbenium ions serve as chain carriers in this model, their concentration directly influences the overall conversion rate. Therefore, the low conversion rate after the transient regime is due to the reduced carbenium ion concentration.

During our parameter optimization, we found that k_{HT} and k_{DP} together govern the transient regime (**Figure S15C**). k_{HT} is positively correlated with the reaction rate, determining how fast isopentane is converted during the transient regime. k_{DP} describes the rate at which carbenium ions transform into alkenes, leading to a carbenium ion-deficient system in the steady-state regime. Essentially, k_{DP} determines the duration of the transient regime. For the simulated curve to fit the experimental data, the k_{HT} to k_{DP} ratio must remain constant. If the k_{HT} to k_{DP} ratio is higher, it implies that either k_{HT} is higher or k_{DP} is lower. The former results in faster product formation, while the latter extends the transient regime, both of which can cause the simulated conversion curve to misalign with the experimental data. Conversely, if the k_{HT} to k_{DP} ratio is lower, it means either k_{HT} is lower or k_{DP} is higher. The former leads to reduced product formation during the transient regime, while the latter shortens the transient regime, preventing accurate fitting (**Figure S15D**). This observation from the kinetic model provides useful insight into our experimental results.

Based on our fitted model, we adjust individual reaction rate constants and observe their impact on reaction behavior. During the transient regime, where the conversion rate varies continuously,

it is more informative to compare the isopentane conversion at 5 minutes rather than the conversion rate itself. In the steady-state regime, since the conversion rate remains approximately constant with time, we compare the reaction rate at 30 minutes. For the sensitivity analysis, we incrementally varied each rate constant by an order of magnitude, recording the simulated isopentane conversion at 5 minutes for the transient regime and the conversion rate at 30 minutes for the steady-state regime. The results for both regimes are illustrated in **Figure S15E** and **S15F**. Firstly, the initiation reaction rate constant (k_{Ini}) does not influence either regime, which is expected due to the rapid formation of active species (the C^+-$AlCl_4^-$ ion-pair, denoted as C^+) through the interaction between TBC and $AlCl_3$*. This reaction occurs significantly faster than all other reactions and, therefore, does not influence the overall kinetic behavior of the system. Secondly, the hydride transfer rate constant (k_{HT}) is positively correlated, while the deprotonation rate constant (k_{DP}) is negatively correlated with the isopentane conversion rate in both regimes (the slight decreases observed at the end of the curves are due to the system approaching 100% isopentane conversion). These correlations are attributable to hydride transfer, the key elementary step in product formation. On the one hand, increasing k_{HT} leads to a higher hydride transfer rate. On the other hand, increasing k_{DP} speeds up the conversion of carbenium ions to alkenes, which reduces the concentration of carbenium ions and thereby slows down isopentane conversion.

The distinction between the transient and steady-state regimes is evident in their sensitivities to the protonation rate constant $k_{-\text{DP}}$. The protonation reaction is the reverse of the deprotonation reaction, converting alkenes back into carbenium ions. In the transient regime, changing $k_{-\text{DP}}$ does not impact the conversion rate since the system remains carbenium ion-rich with a low alkene concentration. An increase in the protonation rate constant cannot substantially raise the carbenium ion concentration or affect the overall reaction rate. Conversely, in the steady-state regime, $k_{-\text{DP}}$ becomes a significant factor, positively influencing the conversion rate. During this regime, the system has a low carbenium ion concentration and a slow hydride transfer rate. Accelerating the protonation reaction effectively converts the accumulated alkenes back into carbenium ions, thereby enhancing the hydride transfer reaction.

The sensitivity analyses of the two regimes can be summarized as follows:

$$\text{Yield}_{\text{transient regime}} \propto k_{\text{HT}}/k_{\text{DP}} \quad (1)$$

$$r_{\text{steady-state regime}} \propto k_{\text{-DP}} \cdot k_{\text{HT}}/k_{\text{DP}} \quad (2)$$

or

$$r_{\text{steady-state regime}} \propto k_{\text{HT}}/K_{\text{DP}} \quad (3)$$

The elementary steps, their corresponding differential equations, and the best-fit rate constants defining the kinetic model are provided in **Table S1**. Since the likelihood functions of these

parameters are not well estimated with normal distributions, we do not report their standard deviations. Instead, the robustness and uncertainty of these parameter estimates were evaluated using an identifiability analysis,[31, 32] with the corresponding likelihood profiles shown in **Figure S16**.

Table S1. Elementary steps and their corresponding best-fit model parameters of the core model presented in **Figure 5A**.

Reaction	Parameter	Value
$TBC \rightarrow C^+$	k_{Ini}	2.87×10^2 s^{-1}
$iC_5 + C^+ \rightarrow P + C^+$	k_{HT}	0.37 M^{-1} s^{-1}
$C^+ \rightleftharpoons C^= + HCl$	k_{DP}	3.54×10^{-2} s^{-1}
	k_{-DP}	1.13×10^{-2} M^{-1} s^{-1}

Differential equations

$$\frac{d[TBC]}{dt} = -k_{Int}[TBC]$$

$$\frac{d[C^+]}{dt} = k_{Int}[TBC] - k_{DP}[C^+] + k_{-DP}[C^=][HCl]$$

$$\frac{d[C^=]}{dt} = k_{DP}[C^+] - k_{-DP}[C^=][HCl]$$

$$\frac{d[P]}{dt} = k_{HT}[iC_5][C^+]$$

$$\frac{d[iC_5]}{dt} = -k_{HT}[iC_5][C^+]$$

$$\frac{d[HCl]}{dt} = k_{DP}[C^+] - k_{-DP}[C^=][HCl]$$

Species	Initial concentration [a]
$[iC_5]_0$	1.50 mmol/mL
$[TBC]_0$	0.0075 mmol/mL, 0.015 mmol/mL, 0.030 mmol/mL

[a] The initial concentration is calculated based on 3.65 mL reaction volume, which approximately equals to the combined volume of DCM (3 mL) and isopentane (5.5 mmol ≙ 0.65 mL). The change in mixture volume due to ionic liquid and TBC is negligible.

A)

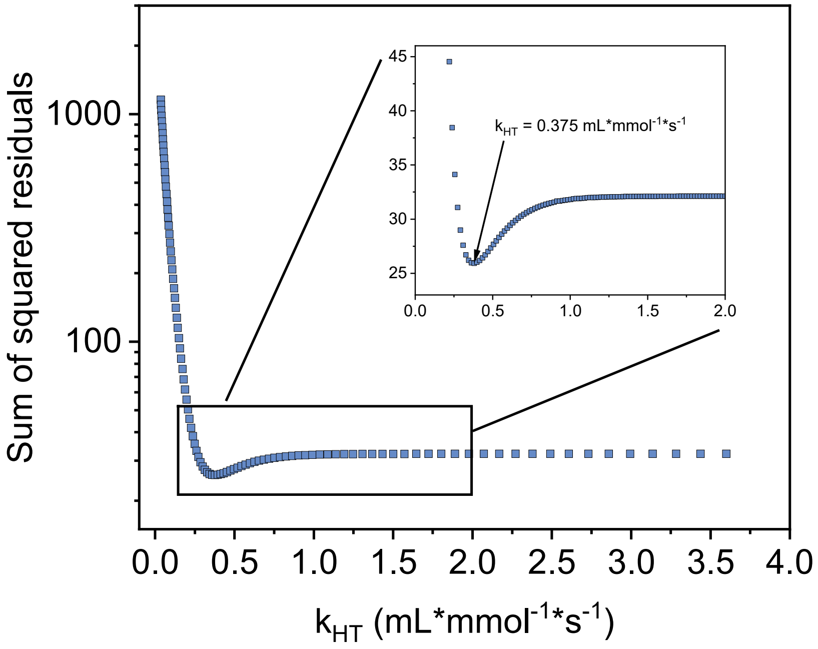

Sum of squared residuals
1000
100
0.0
0.5
1.0
1.5
2.0
2.5
3.0
3.5
4.0
k_HT (mL*mmol^-1*s^-1)
45
40
35
30
25
k_HT = 0.375 mL*mmol^-1*s^-1

B)

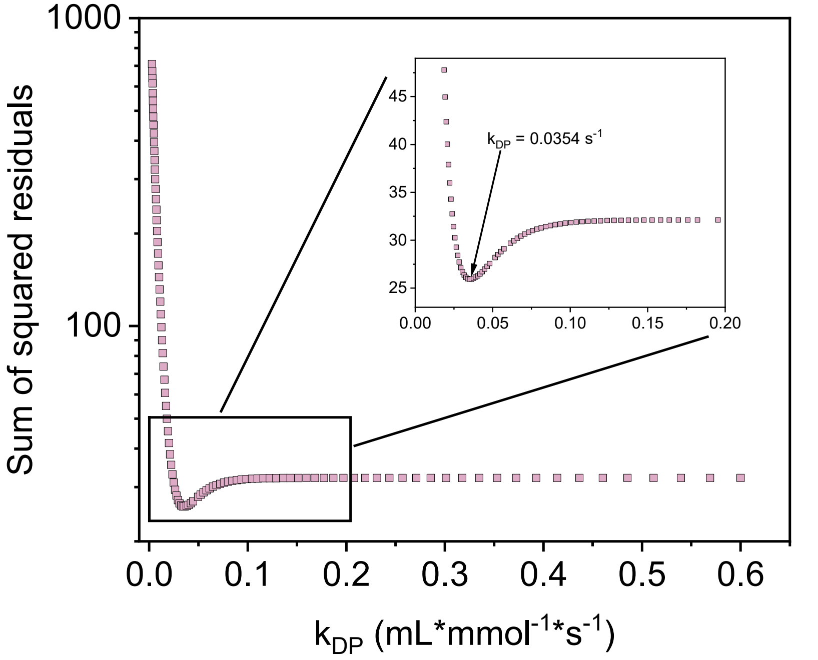

Sum of squared residuals
1000
100
0.0
0.1
0.2
0.3
0.4
0.5
0.6
k_DP (mL*mmol^-1*s^-1)
45
40
35
30
25
0.00
0.05
0.10
0.15
0.20
k_DP = 0.0354 s^-1

C)

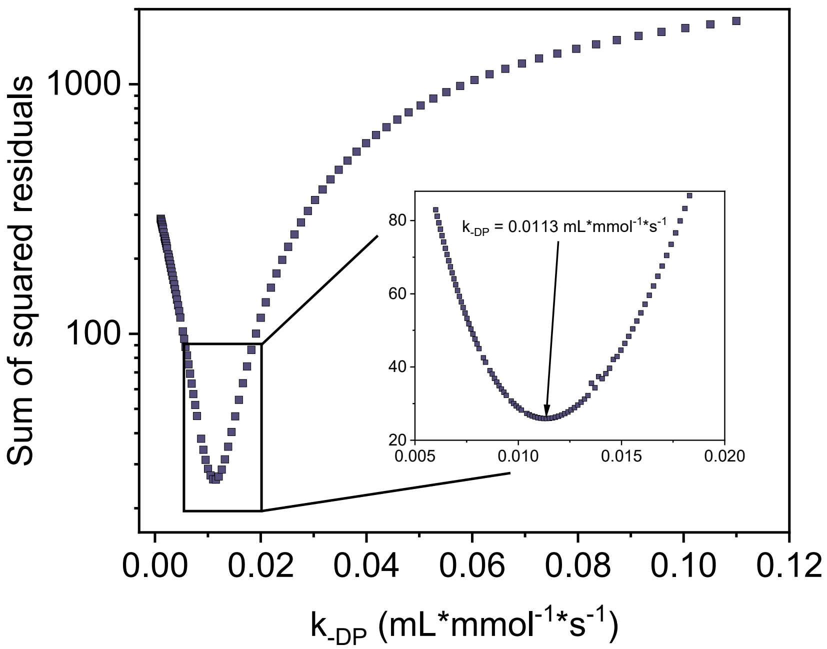

D)

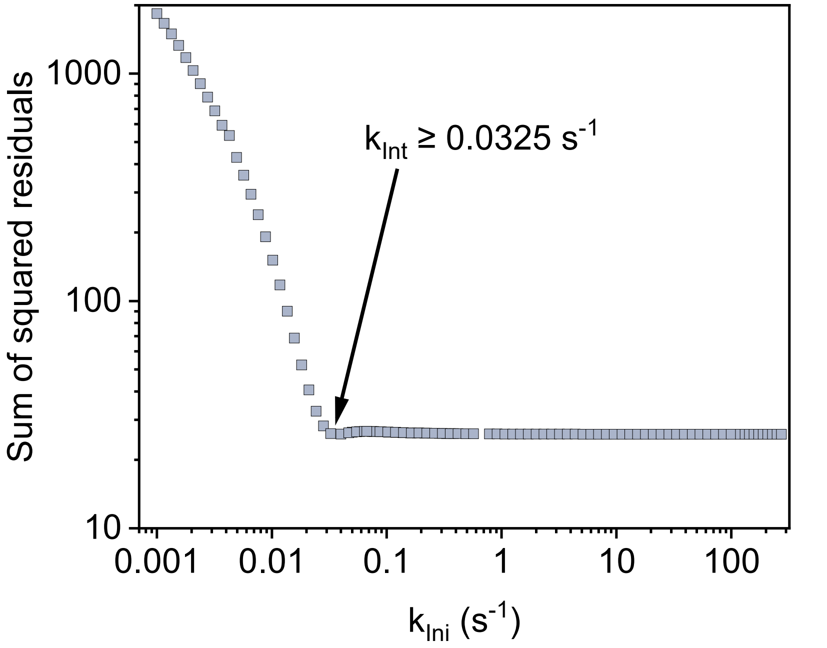

Figure S16. Identifiability analysis of the core model presented in Figure 5A. Identifiability analysis result for the hydride transfer rate constant k_{HT} (**A**), deprotonation rate constant k_{DP} (**B**), protonation rate constant k_{-DP} (**C**), and initiation rate constant k_{Int} (**D**).

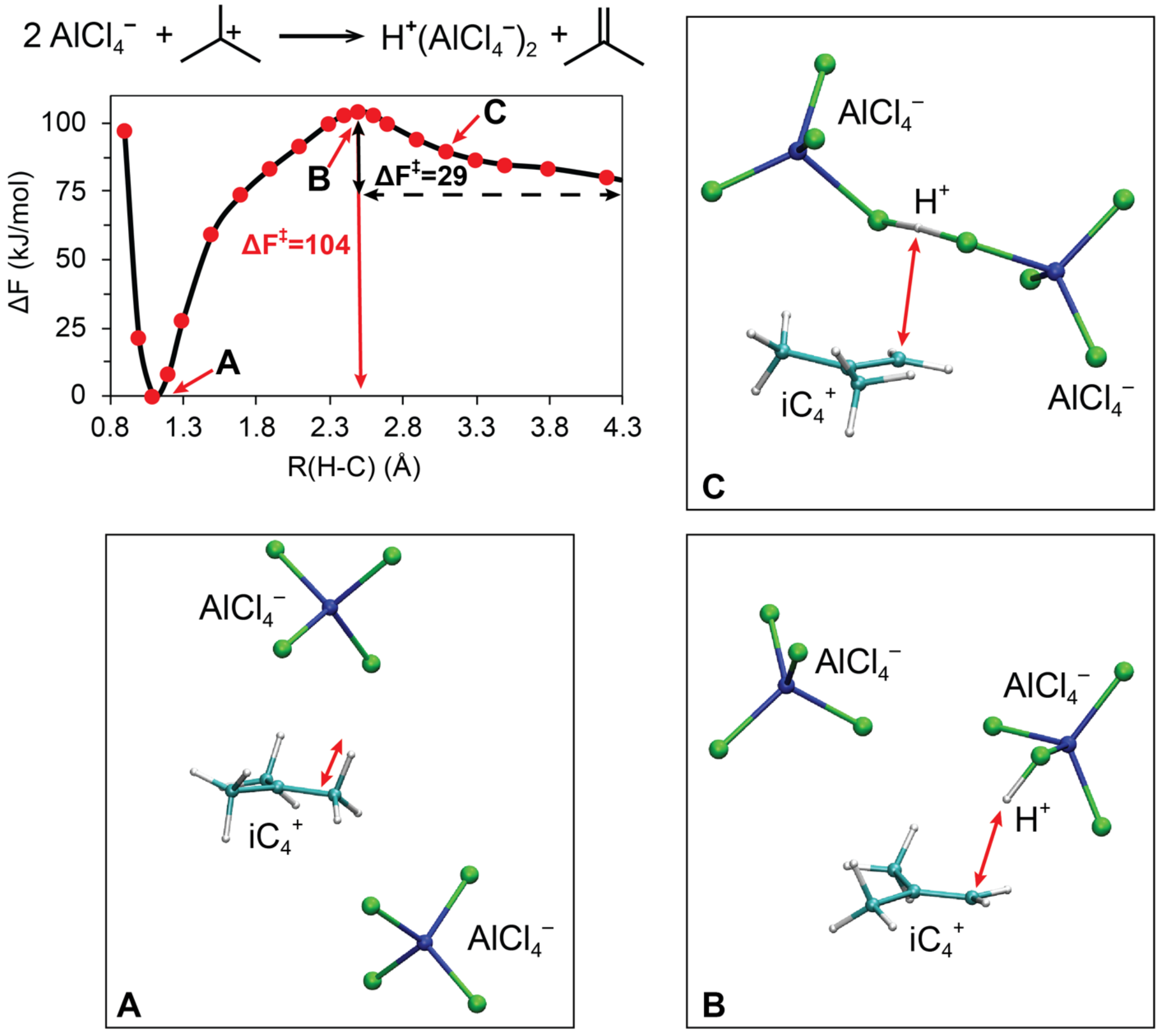

Figure S17. The Helmholtz free energy (ΔF) as a function of the internuclear distance of H-C for the reaction between the 2 $AlCl_4^-$ and iC_4^+. Note that ΔF is calculated using the Blue Moon ensemble approach for *ab initio* molecular dynamics. Included are representative structures along the reaction pathway; a red arrow is added to the structures to indicate the internuclear distance constrained in the Blue Moon calculations. See **Table S2** for structure of the simulation cell.

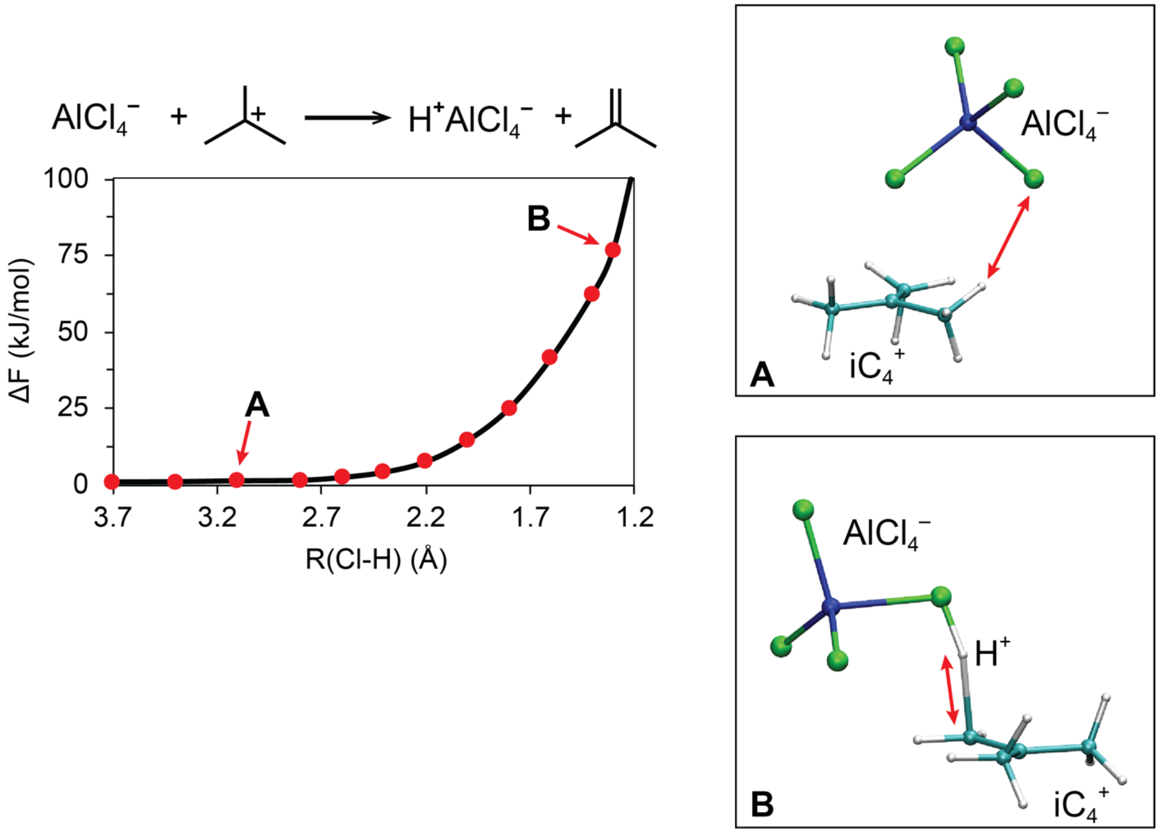

Figure S18. The Helmholtz free energy (ΔF) as a function of the internuclear distance of Cl-H for the reaction between $AlCl_4^-$ and iC_4^+. Note that ΔF is calculated using the Blue Moon ensemble approach for *ab initio* molecular dynamics. Included are representative structures along the reaction pathway; a red arrow is added to the structures to indicate the internuclear distance constrained in the Blue Moon calculations. See **Table S2** for structure of the simulation cell.

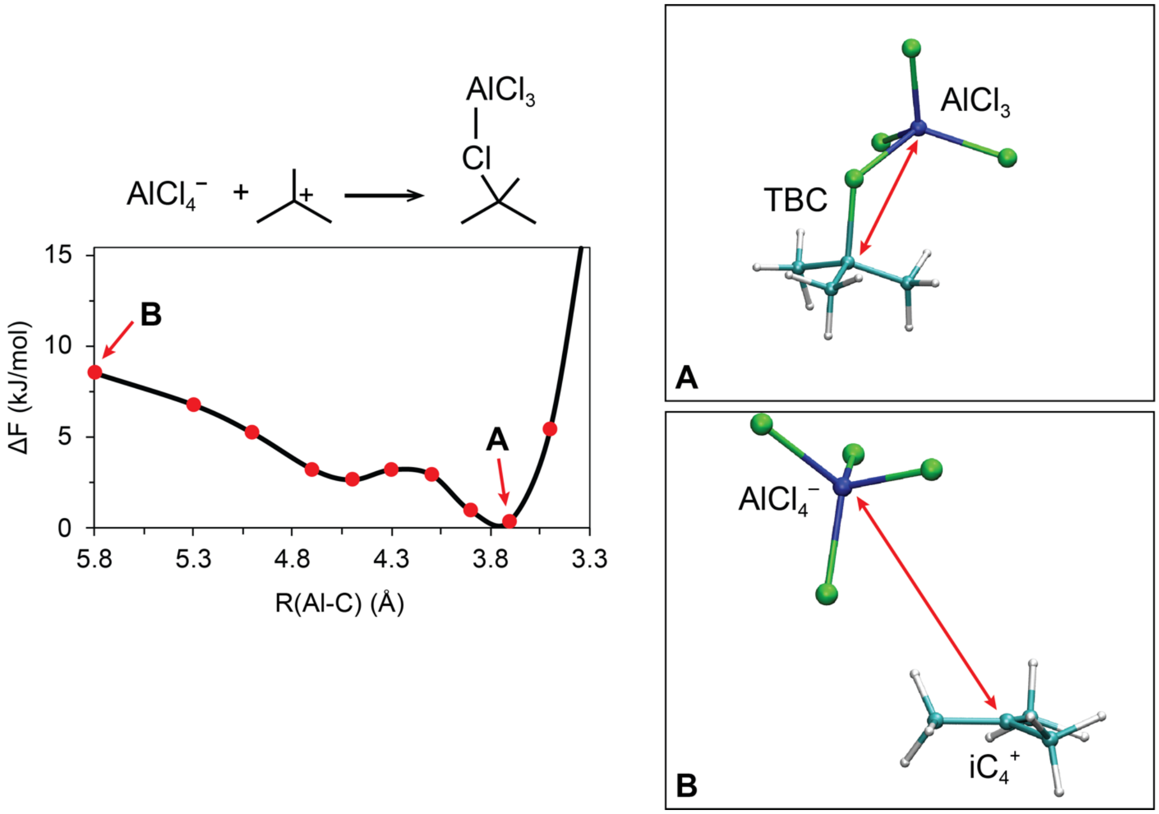

Figure S19. The Helmholtz free energy (ΔF) as a function of the internuclear distance of Al-C for the deprotonation of TBC by $AlCl_3$. Note that ΔF is calculated using the Blue Moon ensemble approach for *ab initio* molecular dynamics. Included are representative structures along the reaction pathway; a red arrow is added to the structures to indicate the internuclear distance constrained in the Blue Moon calculations. See **Table S2** for structure of the simulation cell.

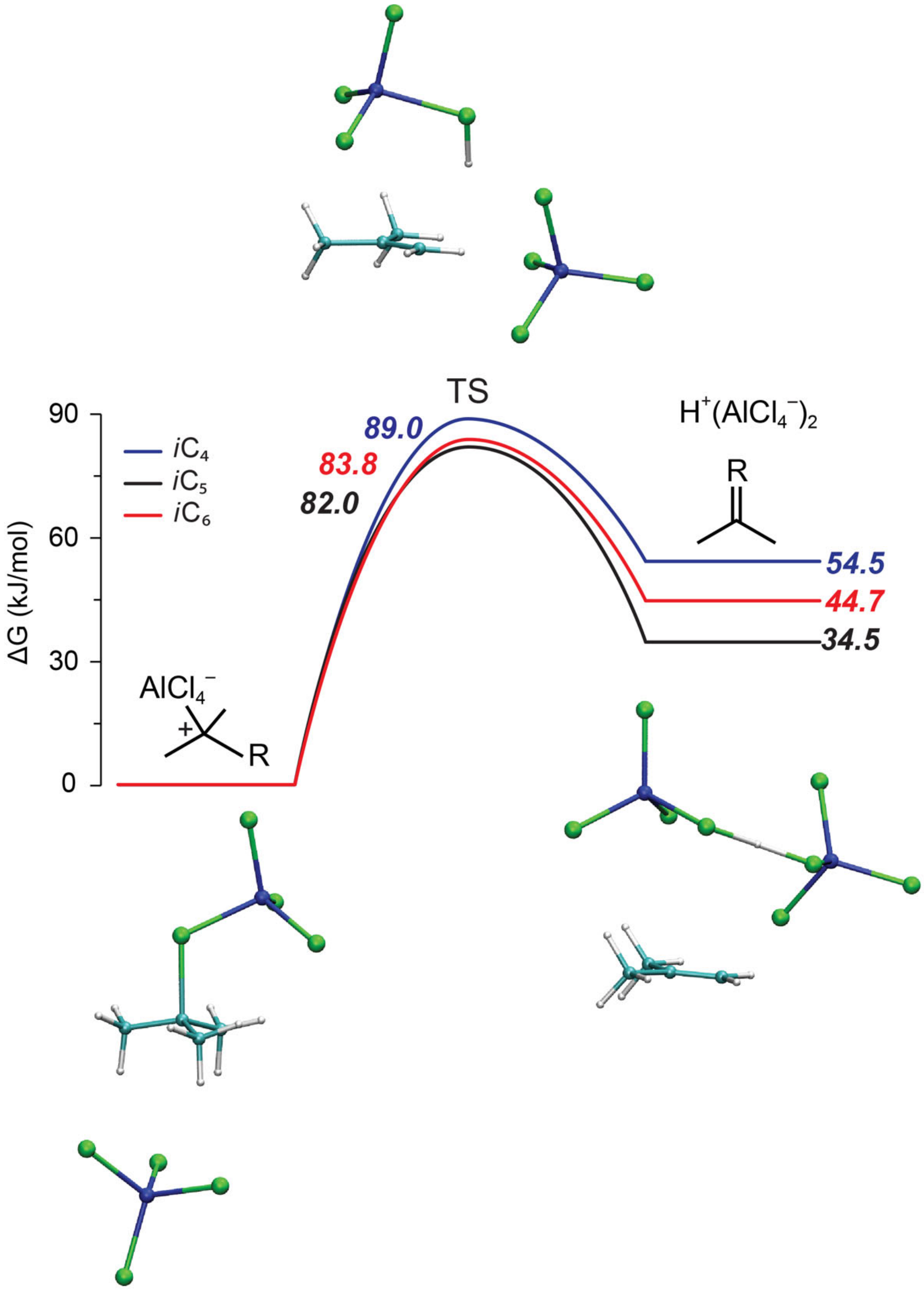

Figure S20. The Gibbs free energy (ΔG) for the reaction of 2 $AlCl_4^-$ and iC_4^+, iC_5^+, or iC_6^+. Transition state structures obtained first from a nudged elastic band calculation followed by a transition state optimization using the DIMER method. Structures of the iC_4 reactant, transition state, and products are included as insets. See **Tables S3-S5** for structures.

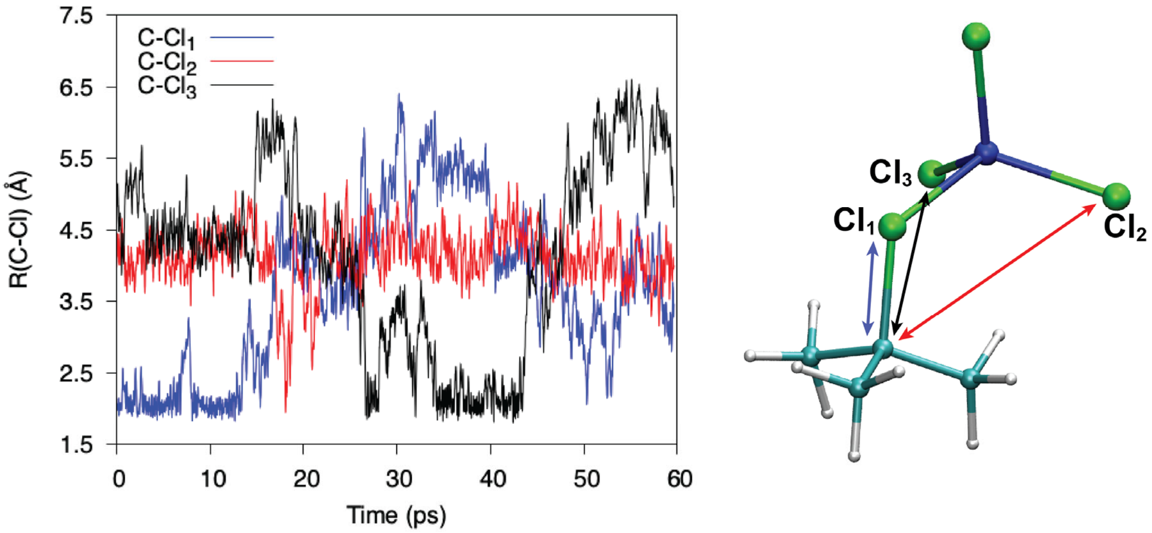

Figure S21. Plot of the C-Cl distances within the TBC-$AlCl_3$ complex during the simulated NVT trajectory. Illustrates the complex to be an iC_4^+-$AlCl_4^-$ ion-pair as the weak interaction enables $AlCl_4^-$ to freely rotate, exchanging which Cl coordinates to the carbocation.

Table S2. Cartesian coordinates (Å) of the simulation cell modeling the conditions of the experimental solution. The cell was equilibrated at 298 K for 60 ps. The structure provided is the last structure of the NVT trajectory as an example structure.

Cartesian coordinates (Å) of simulated solution																			
N	20.5128	13.5657	8.7976	H	12.0739	5.0477	4.1581	H	10.2491	27.2601	-7.8796	Cl	4.6097	15.0986	22.5395	C	-0.4328	11.9235	-10.0563
C	21.3290	14.5918	9.1481	C	9.0486	25.9829	10.8731	H	9.8778	28.8620	-7.1501	H	6.1679	16.0630	21.1263	Cl	-1.5200	10.6330	-10.6404
N	20.6034	15.7271	9.1658	C	7.9377	25.2116	10.0314	Cl	0.4200	12.6776	3.9915	H	6.8215	15.8332	22.8664	H	-0.1257	11.7192	-8.9753
C	19.2634	15.4268	9.0186	C	8.0035	23.6390	10.0045	C	0.6274	14.1241	5.1060	Cl	15.5826	23.0497	3.0290	H	0.4945	12.0496	-10.6856
C	19.2316	14.0298	8.7500	C	7.0314	23.2475	8.9392	Cl	-0.9129	15.0164	5.3205	C	16.3252	24.5350	3.8269	Cl	14.0831	6.6143	8.8672
C	20.7355	12.1110	8.7029	C	9.4436	23.1474	9.7440	H	1.3670	14.8607	4.6876	Cl	16.7538	25.9852	2.9023	C	14.2078	5.3687	10.2180
H	22.4403	14.5576	9.1810	H	8.6943	27.0275	10.8072	H	0.8879	13.7924	6.1668	H	17.2218	24.1761	4.2245	Cl	12.6029	4.7984	10.5764
C	21.1774	17.0709	9.4161	H	10.0119	25.8955	10.3758	Cl	1.8277	33.0892	-7.4708	H	15.7379	24.7876	4.7024	H	14.6915	5.8160	11.1065
H	18.4165	16.1633	9.0854	H	8.9861	25.7543	11.9306	C	0.6738	31.8194	-7.7076	Cl	15.3642	3.7503	-4.2206	H	14.8654	4.5759	9.8759
H	18.3803	13.4234	8.5034	H	8.0685	25.5401	8.9790	Cl	0.6092	30.7570	-6.2122	C	16.9318	4.5881	-3.5104	Cl	28.4261	-1.5444	17.5271
H	20.0898	11.6913	7.9291	H	6.9353	25.5099	10.3918	H	0.9334	31.2638	-8.6217	Cl	16.6520	6.2681	-3.2212	C	28.9512	-1.7882	15.8686
H	21.8050	11.9465	8.4742	H	7.5657	23.3430	10.9600	H	-0.3375	32.2378	-7.9697	H	17.7263	4.4132	-4.2388	Cl	30.2370	-0.5915	15.5104
H	20.5560	11.5954	9.6045	H	6.8798	22.1550	8.9665	Cl	-0.8708	25.1338	6.9665	H	17.1122	4.0170	-2.5759	H	28.0932	-1.6369	15.2199
C	21.5809	17.6365	7.9667	H	7.4901	23.4855	7.9403	C	-2.6156	25.2162	6.8814	Cl	-2.0781	17.4663	21.7636	H	29.4417	-2.7749	15.8662
H	20.4626	17.7520	9.9558	H	6.0878	23.8338	9.0786	Cl	-3.1422	25.3106	5.2188	C	-1.5859	15.7356	21.6668	Cl	5.6526	20.0642	-2.3245
H	22.0441	16.7923	10.0888	H	9.7648	23.4375	8.7332	H	-3.0323	26.1065	7.4102	Cl	-2.4159	15.0345	20.2507	C	5.9840	21.1516	-0.9570
H	22.2525	16.9256	7.4395	H	9.5542	21.9981	9.8337	H	-2.9660	24.2619	7.2206	H	-1.8623	15.2767	22.6494	Cl	4.6227	21.4721	0.0773
H	20.6999	17.9477	7.3315	H	10.3103	23.4857	10.3821	Cl	17.8570	13.5670	16.1199	H	-0.5252	15.8564	21.5587	H	6.3093	22.1541	-1.4049
H	22.0502	18.5729	8.0963	C	17.8945	-1.2045	16.9215	C	19.0303	12.4365	15.3595	Cl	28.9821	16.2149	4.1091	H	6.7858	20.7444	-0.2953
N	11.6829	14.2886	9.3576	C	18.6656	-0.2922	17.7015	Cl	18.6803	10.7099	15.9330	C	28.3840	15.6309	5.7396	Cl	20.9638	18.6485	17.4441
C	12.1636	15.5318	9.7330	C	18.1265	0.9783	18.2150	H	19.9844	12.6845	15.6839	Cl	28.9206	16.4435	7.2238	C	21.5251	16.9545	17.4445
N	11.1090	16.3033	10.1006	C	20.1193	-0.5568	17.8671	H	18.9605	12.4736	14.2714	H	28.6682	14.5766	5.8502	Cl	20.4610	15.8755	16.4748
C	9.9975	15.5049	10.0903	Cl	17.1649	1.7288	15.3134	Cl	23.6412	23.6485	3.7703	H	27.3375	15.7534	5.6476	H	21.5092	16.5993	18.4801
C	10.2985	14.2629	9.6111	H	16.8674	-0.8306	16.8607	C	25.3202	23.4525	2.9795	Cl	16.1396	18.3330	17.4347	H	22.4210	16.8644	16.9508
C	12.4859	13.2252	8.6487	H	18.1271	-2.2629	17.1162	Cl	26.0596	21.9639	3.5522	C	16.4185	16.8138	16.6166	Cl	8.5178	11.0884	12.8330
H	13.2089	15.8132	9.6553	H	18.3655	-1.1357	15.9055	H	25.9775	24.2236	3.3625	Cl	16.0627	16.7641	14.7674	C	9.2957	10.0962	14.1337
C	11.1658	17.6634	10.6688	H	18.7628	1.7873	17.7205	H	25.1123	23.3691	1.8934	H	15.6980	16.0909	17.0140	Cl	9.9227	11.1666	15.3842
H	8.9886	15.7695	10.3595	H	18.3806	0.9928	19.2784	Cl	11.3719	13.4777	-0.5827	H	17.5130	16.5781	16.6434	H	10.1059	9.6187	13.5937
H	9.5867	13.5133	9.2728	H	17.0472	1.2313	18.1244	C	12.2370	14.4994	0.4994	Cl	13.6911	24.2520	23.1204	H	8.5894	9.3977	14.5701
H	11.8699	12.7434	7.8892	H	20.4847	-0.5863	18.9214	Cl	13.5221	15.5235	-0.1985	C	14.7176	25.4722	22.3157	Cl	13.1751	6.9373	-2.3146
H	13.2502	13.7894	8.0821	H	20.6918	0.3151	17.4757	H	12.7908	13.9283	1.2155	Cl	16.2323	24.7404	21.5508	C	11.5028	6.5676	-2.5052
H	12.9267	12.4877	9.3852	H	20.4949	-1.5130	17.3010	H	11.4651	15.0761	0.9737	H	15.0058	26.2158	23.0985	Cl	11.0349	6.9935	-4.2321
C	10.7234	18.8566	9.7984	Cl	9.0835	16.2462	19.5788	Cl	22.7102	24.8904	15.3640	H	14.0908	25.8327	21.5457	H	10.9148	7.2250	-1.8823
H	10.5563	17.6451	11.5835	C	9.6539	17.1402	20.9669	C	24.4680	25.3924	15.6662	Cl	25.6038	3.1493	10.6135	H	11.3138	5.4598	-2.3488
H	12.2558	17.7662	10.9490	Cl	8.8570	18.6858	21.3048	Cl	25.1463	24.5039	17.0272	C	24.5473	1.8805	11.2579	Cl	14.2694	20.9880	17.5331
H	9.6476	18.7109	9.4789	H	10.7421	17.2952	20.7819	H	24.5100	26.4877	15.9836	Cl	22.7704	2.2752	11.2915	C	13.2871	22.3430	18.3883
H	10.9081	19.8019	10.3089	H	9.5012	16.4919	21.8687	H	25.1949	25.3998	14.8454	H	24.8173	1.6760	12.3021	Cl	14.1479	23.8989	18.3018
H	11.2750	18.8284	8.8325	Cl	12.9346	21.0161	22.5779	Cl	9.0449	22.1627	21.4205	H	24.7156	0.9816	10.6577	H	13.0702	21.9486	19.4063
Al	2.9314	15.7350	9.7137	C	12.9148	19.2473	22.3360	C	10.0993	21.8995	20.0137	Cl	9.1728	10.8901	-0.0467	H	12.4004	22.4356	17.7353
Cl	2.7379	14.9505	7.7838	Cl	13.8838	18.7625	20.8882	Cl	9.2951	22.4487	18.5151	C	8.5072	11.3942	1.5685	Cl	17.6516	9.8586	12.6027
Cl	4.8418	15.5786	10.5373	H	11.8310	18.9850	22.1582	H	10.2519	20.8260	19.9931	Cl	8.9960	13.1363	2.0294	C	17.6136	8.1474	12.8638
Cl	1.6696	14.5936	10.9897	H	13.3952	18.7459	23.1424	H	10.9834	22.4685	20.2613	H	8.8828	10.7095	2.3893	Cl	15.9401	7.5041	12.9108
Cl	1.9997	17.7606	9.7867	Cl	20.6571	15.8890	25.0239	Cl	13.1299	21.3640	10.5447	H	7.3918	11.4139	1.4602	H	17.9873	7.9070	13.8310
Al	15.4669	18.2479	8.0088	C	21.4599	17.3783	25.5604	C	13.1537	22.1951	8.9586	Cl	9.0393	13.8533	5.1002	H	18.1938	7.6396	12.0431
Cl	14.3758	16.4267	7.1694	Cl	20.3618	18.8069	25.8357	Cl	13.1304	23.9876	9.1020	C	9.6729	15.4674	5.5616	Cl	27.0471	6.8774	-3.2516
Cl	14.4354	19.7508	6.8320	H	22.2447	17.6127	24.8315	H	12.3765	21.9798	8.3015	Cl	11.3222	15.4487	6.3750	C	26.2460	5.3316	-3.4141
Cl	17.6829	18.1041	7.4562	H	21.8349	17.1481	26.5583	H	14.1354	22.0104	8.5217	H	8.9770	15.9609	6.2785	Cl	26.2078	4.7333	-5.1344
Cl	15.0550	18.2518	10.1042	Cl	18.9912	4.4229	-0.4893	Cl	5.4048	21.0374	11.3266	H	9.8271	16.0585	4.6960	H	25.1968	5.4167	-3.1872
Al	18.9107	2.8731	14.8942	C	19.5721	5.8744	0.3716	C	4.5865	19.4377	11.3234	Cl	10.9262	19.2020	17.8287	H	26.8714	4.6313	-2.8632
Cl	19.7655	3.4637	16.9095	Cl	20.9221	6.6387	-0.5072	Cl	4.7811	18.5113	12.8478	C	9.5992	19.4289	16.6607	Cl	9.4967	1.5336	15.2111
Cl	18.1346	4.6859	14.0723	H	20.0655	5.5899	1.3294	H	5.0532	18.7809	10.5280	Cl	8.0436	18.6991	17.2161	C	10.8748	0.7052	14.4579
Cl	20.0507	1.8296	13.4259	H	18.8207	6.6295	0.5367	H	3.5121	19.5356	11.0900	H	9.8768	18.9087	15.7152	Cl	11.5190	-0.7301	15.3656
Al	7.3410	10.1027	6.7021	Cl	-2.0888	-2.3870	10.1374	Cl	16.6311	15.9511	20.2657	H	9.4550	20.4852	16.4342	H	11.7162	1.3604	14.5017
Cl	7.1713	10.4470	4.6482	C	-2.0469	-0.9120	9.1343	C	16.9371	14.5251	21.2989	Cl	26.5450	8.4275	12.2821	H	10.6721	0.3352	13.3713
Cl	5.8835	9.2137	7.9098	Cl	-1.5911	-1.2712	7.4431	Cl	17.7740	14.9567	22.8173	C	25.1676	8.1568	11.2296	Cl	10.0814	21.1277	7.2757
Cl	9.3418	10.2355	7.3362	H	-1.2885	-0.2813	9.5538	H	17.5873	13.8226	20.7833	Cl	25.4490	6.6214	10.2854	Cl	9.3273	18.5942	5.9515
C	21.7255	-1.4849	-0.2293	H	-3.0324	-0.5015	9.0730	H	15.9849	14.0063	21.5521	H	25.0033	9.0290	10.5274	C	10.5390	19.9113	6.0703
C	20.9533	-0.4516	0.7202	Cl	9.7362	12.1727	-3.6772	Cl	18.3511	1.1805	10.3340	H	24.2759	8.0692	11.8468	H	10.5464	20.4944	5.1183
C	19.5548	-0.0978	0.0904	C	10.8876	10.7831	-4.1152	C	17.5031	-0.1865	11.1898	Cl	24.6309	37.8144	-2.2602	H	11.5277	19.5552	6.2742
C	18.5825	0.3874	1.2443	Cl	11.9974	10.3593	-2.8491	Cl	16.7435	-1.2801	9.9850	C	24.4731	36.3248	-3.1958	Cl	6.4303	9.7519	-2.6520
C	19.7203	0.8858	-1.1349	H	11.5063	11.0656	-4.9652	H	16.8080	0.2541	11.8665	Cl	25.1610	34.9637	-2.2758	Cl	4.6220	8.9671	-0.4627
H	22.7984	-1.6294	0.0587	H	10.3483	9.7849	-4.3348	H	18.3019	-0.6820	11.7466	H	25.0755	36.4807	-4.0964	C	5.8550	10.1581	-1.0611
H	21.7831	-0.9248	-1.1732	Cl	28.2852	17.1958	15.9441	Cl	19.1102	-2.5325	13.4765	H	23.4038	36.1465	-3.4244	H	6.7884	10.1701	-0.4181
H	21.1487	-2.4043	-0.3771	C	27.8767	16.6015	17.6248	C	20.8017	-2.5036	13.9849	Cl	-2.0318	10.4501	23.4341	H	5.4250	11.1833	-1.0029
H	21.5515	0.4203	0.9900	Cl	28.3348	17.6287	18.9787	Cl	21.3690	-1.0522	14.7184	C	-3.4237	10.6430	22.3972	Cl	-0.1317	9.4751	6.4097
H	20.8037	-0.9438	1.6691	H	28.2876	15.6393	17.7180	H	21.4812	-2.6129	13.0705	Cl	-5.0144	11.0604	23.3167	Cl	1.4572	10.8335	8.4667
H	19.0438	-1.0690	-0.3172	H	26.7793	16.6469	17.5794	H	20.9201	-3.4006	14.6418	H	-3.6247	9.7511	21.8515	C	1.0106	9.3085	7.6998
H	18.9081	1.2779	1.8187	Cl	6.4292	12.5152	7.4467	Cl	6.8807	31.6231	-5.9994	H	-3.3936	11.5196	21.7873	H	0.5923	8.7488	8.5420
H	18.4991	-0.4585	2.0035	C	5.5569	12.5724	9.0675	C	5.5659	31.3888	-7.0566	Cl	13.7075	12.0753	5.3662	H	1.9132	8.8053	7.3667
H	17.4696	0.4210	1.0149	Cl	6.6172	12.4729	10.4624	Cl	6.1108	30.8324	-8.6633	C	15.1982	12.9492	5.6781	Cl	12.6832	16.4601	-4.6620
H	18.7352	1.1177	-1.5663	H	4.7801	11.7774	9.0471	H	5.0503	32.3884	-7.2025	Cl	16.2774	12.3299	6.9064	Cl	13.7637	13.7135	-5.2062
H	20.3291	0.5376	-1.9720	H	5.1481	13.5635	9.0818	H	4.8369	30.7431	-6.5988	H	14.9525	13.9656	6.0259	C	13.2789	14.9282	-3.9513
H	20.0371	1.9153	-0.7703	Cl	3.5339	9.3090	4.7476	Cl	24.6259	15.4768	16.2243	H	15.7839	13.0137	4.7765	H	14.1519	15.1520	-3.3254
C	4.9925	5.9055	5.9737	C	2.7454	9.1044	3.1158	C	24.4196	16.0830	14.4857	Cl	19.9407	30.3453	18.9009	H	12.4440	14.5080	-3.4573
C	6.4109	5.4242	6.5514	Cl	1.0788	8.6519	3.1690	Cl	23.9550	17.8007	14.4877	C	19.2228	29.7367	17.3332	Cl	19.9780	4.5101	10.1326
C	6.3439	4.7774	7.9161	H	3.2438	8.3189	2.5976	H	23.5958	15.4957	14.0853	Cl	17.7949	30.7788	16.8774	Cl	17.3628	5.3228	8.9824
C	5.9034	5.7071	9.0585	H	2.9254	10.0284	2.5376	H	25.4153	15.9671	14.0061	H	18.8996	28.7758	17.6669	C	18.2378	4.6578	10.4307
C	5.5229	3.4097	7.8269	Cl	8.0300	24.4311	5.0522	Cl	12.0072	4.3631	1.2566	H	20.0482	29.6240	16.6360	H	17.8569	3.7118	10.7764
H	5.1837	6.4624	5.0290	C	6.4293	23.8387	4.5862	C	12.9833	5.7694	1.6948	Cl	-0.3546	13.3048	0.7064	H	18.0856	5.4338	11.1750
H	4.5071	6.5560	6.7071	Cl	5.3687	25.1115	3.7798	Cl	14.7965	5.8088	1.1525	C	1.3184	13.3880	1.0760	Cl	20.9455	16.4265	12.7174
H	4.3617	5.0421	5.7639	H	5.8706	23.4183	5.4277	H	12.9230	5.8288	2.8280	Cl	1.6283	15.0316	1.7896	Cl	18.4360	17.3751	11.5215
H	7.0368	6.3189	6.5303	H	6.5438	23.0725	3.8378	H	12.6025	6.6241	1.1258	H	2.0086	13.3248	0.2270	C	19.1891	16.7520	13.0032
H	6.7517	4.6922	5.8379	Cl	21.5279	2.6209	19.5450	Cl	11.6805	26.6817	-5.2401	H	1.6146	12.6493	1.8392	H	18.6951	15.7842	13.2318
H	7.3257	4.5009	8.2727	C	22.6974	3.6777	18.7278	C	10.2640	26.5763	-4.1927	Cl	9.1065	16.3572	1.6655	H	18.9932	17.4319	13.8233
H	6.4156	5.4185	9.9417	Cl	22.9060	5.2189	19.6108	Cl	10.5649	25.9093	-2.5728	C	9.2968	18.0175	2.4471	Cl	21.3976	8.3066	9.7616
H	4.8306	5.7353	9.3361	H	22.3142	3.7600	17.6964	H	9.5198	25.9415	-4.6621	Cl	9.7672	19.2676	1.2451	Cl	21.6700	6.4577	7.5298
H	6.1002	6.7316	8.9055	H	23.7109	3.3245	18.7224	H	9.8660	27.5612	-4.0327	H	10.0928	17.9777	3.1573	C	20.6440	7.6766	8.2231
H	5.8962	2.8929	6.9685	Cl	14.4718	8.2078	17.0142	Cl	12.2081	8.4395	6.9065	H	8.3385	18.2788	2.8490	H	19.6259	7.2495	8.5051
H	4.4513	3.6415	7.7293	C	13.7571	6.6178	16.5378	C	13.3453	8.5898	5.3953	Cl	10.7643	8.3274	-13.0421	H	20.5966	8.5161	7.5705
H	5.7912	2.7169	8.6410	Cl	12.7948	6.6535	14.9913	Cl	14.8973	7.8158	5.6645	C	8.9740	7.9257	-13.2458	Cl	14.3081	1.9789	12.7152
C	18.5447	5.9505	5.1888	H	13.0357	6.3883	17.2876	H	13.4669	9.6066	5.1057	Cl	8.6433	6.5329	-12.1289	C	14.4569	3.3924	13.8091
C	18.4106	7.4428	5.6524	H	14.6617	5.9998	16.3474	H	12.7946	8.0751	4.5853	H	8.8222	7.5978	-14.2294	H	13.9100	4.1936	13.2566
C	18.7851	8.3478	4.5145	Cl	23.3664	20.4043	19.0342	Cl	15.7912	21.7330	14.2928	H	8.3391	8.7982	-13.0060	H	15.5460	3.6493	13.9930
C	18.2279	9.7816	4.4395	C	24.9674	20.8394	18.2090	C	15.6295	20.2834	13.2895	Cl	22.1376	5.7090	2.6001	Cl	13.5875	3.0444	15.3017
C	20.3286	8.2892	4.2114	Cl	25.3728	19.5400	16.9651	Cl	14.0738	19.3625	13.4916	C	22.9050	4.3173	1.7358	Cl	14.7173	10.2264	10.3445
H	17.8213	5.3428	5.7734	H	25.7100	20.8123	19.0153	H	16.4022	19.5832	13.5563	Cl	21.6481	3.0790	1.7229	C	15.9429	9.7709	9.0396
H	19.4762	5.4248	5.3472	H	24.7787	21.7350	17.5987	H	15.7514	20.5881	12.2476	H	23.7566	3.9260	2.2493	H	15.5064	9.9884	8.0837
H	18.2598	5.8081	4.1379	Cl	17.8497	22.7488	6.6713	Cl	1.7782	6.7586	0.3793	H	23.0049	4.5325	0.6803	H	16.1297	8.7006	9.0450

H	19.1023	7.6154	6.5231	C	18.6236	21.2454	5.8322	C	1.0068	8.1957	-0.2426	Cl	6.6638	26.1675	-4.0878	Cl	17.4980	10.5529	9.0984
H	17.3418	7.5725	5.9664	Cl	19.9507	21.9136	4.7605	Cl	0.4290	8.1794	-1.9308	C	5.8940	24.5284	-4.3787	Cl	8.8135	14.2935	16.2040
H	18.3529	7.8195	3.6466	H	17.9254	20.7585	5.1161	H	1.7020	9.0091	-0.2022	Cl	4.6724	24.1179	-3.0646	C	8.0181	15.4014	15.1374
H	18.5597	10.3198	3.5861	H	19.0788	20.6493	6.6189	H	0.2256	8.4985	0.4390	H	6.6634	23.7569	-4.3210	H	8.7449	16.1167	14.7856
H	18.5562	10.4719	5.2098	Cl	16.0372	15.8597	26.7969	Cl	25.5537	22.3882	13.3392	H	5.3455	24.4370	-5.3004	H	7.2091	15.8832	15.6076
H	17.1391	9.7024	4.4628	C	17.6395	15.1398	26.7899	C	24.7949	20.9297	12.9316	Cl	12.3359	22.8380	5.5978	Cl	7.3201	14.6525	13.6700
H	20.7105	7.2734	4.1019	Cl	18.5779	15.4145	28.2942	Cl	24.2581	21.0037	11.2000	C	11.9358	23.0894	3.8625	C	2.5140	23.0509	6.9777
H	20.9696	8.7945	4.9998	H	18.3432	15.5872	26.1007	H	23.8325	20.8024	13.5115	Cl	10.5293	22.0457	3.2907	H	1.5635	23.5943	7.0779
H	20.5269	8.7269	3.2550	H	17.5225	14.0685	26.5410	H	25.5526	20.1850	12.9646	H	12.7675	22.8205	3.1234	Cl	2.0920	21.4841	7.7982
C	15.4439	12.8368	25.0906	Cl	6.1673	7.1441	2.0432	Cl	6.7615	19.3874	8.4368	H	11.7278	24.1518	3.7013	Cl	3.8601	23.8966	7.7515
C	14.7682	11.8188	24.1344	C	5.8707	5.4680	2.4311	C	5.1792	19.8864	7.7698	Cl	8.1957	18.3859	12.7934	H	2.8474	22.7819	5.9536
C	14.0939	10.6351	24.8792	Cl	5.2704	4.5702	0.9301	Cl	5.4127	20.4484	6.1559	C	8.6916	20.1240	12.8882	C	21.8950	28.3718	-9.5325
C	13.3811	9.8430	23.7328	H	4.9999	5.3627	3.0972	H	4.7744	20.7500	8.2575	Cl	10.4461	20.2710	12.9107	H	21.7498	28.0578	-8.4866
C	14.9988	9.6561	25.5971	H	6.7830	4.9859	2.7572	H	4.4640	19.0735	7.7672	H	8.3817	20.5681	13.8696	Cl	23.6529	28.4307	-9.7060
H	16.1476	13.5136	24.6347	Cl	29.7724	20.4627	2.9791	Cl	8.9252	5.5311	-0.1405	H	8.3983	20.6820	12.0245	Cl	21.0893	29.9601	-9.7890
H	16.0964	12.2870	25.8413	C	28.6381	19.1304	2.5634	C	7.5400	5.8608	-1.2340	Cl	12.9118	16.5107	26.3373	H	21.4712	27.6077	-10.2328
H	14.7243	13.3667	25.6828	Cl	28.8742	18.1900	1.0730	Cl	7.8347	6.4202	-2.9359	C	13.4632	18.0551	27.0024	C	17.6278	19.7832	1.0201
H	15.6189	11.4433	23.4860	H	27.6326	19.5619	2.5601	H	6.9448	6.6755	-0.8396	Cl	13.5893	19.4528	25.8668	H	18.6982	19.6750	1.0689
H	14.0227	12.3292	23.4008	H	28.7224	18.3778	3.3721	H	6.9996	4.9275	-1.3411	H	14.5184	17.9600	27.3540	Cl	17.1322	19.8241	2.7907
H	13.3782	11.0221	25.6002	Cl	4.2438	11.9657	-5.1512	Cl	2.6890	17.5468	4.8367	H	12.9780	18.3341	27.9146	Cl	17.1778	18.2134	0.2747
H	14.0825	9.5073	22.9657	C	5.6257	12.5817	-6.0631	C	1.8563	18.8797	5.8367	Cl	12.0802	14.1716	13.4147	H	17.1238	20.6502	0.5420
H	12.5082	10.4119	23.3787	Cl	5.9953	11.6843	-7.6657	Cl	0.2797	19.4687	5.3089	C	12.2641	15.5031	14.5895	C	25.7134	18.2623	22.9378
H	12.7830	8.9024	24.0338	H	6.5862	12.4160	-5.5231	H	2.5805	19.7440	5.8344	Cl	11.2912	16.9638	14.0946	H	26.2642	17.2765	23.0045
H	14.5219	8.7206	26.0097	H	5.4278	13.6250	-6.2993	H	1.8385	18.4102	6.8670	H	13.3667	15.6806	14.6492	Cl	25.0139	18.4761	24.5975
H	15.4072	10.0744	26.5458	Cl	4.7387	27.1437	14.5989	Cl	25.0315	26.9717	6.7898	H	11.8641	15.1524	15.6277	Cl	24.4609	18.1734	21.7015
H	15.8106	9.2908	24.9511	C	5.5564	26.7507	13.0097	C	24.2652	27.1291	5.1719	Cl	28.1923	12.4120	1.7993	H	26.3242	19.1737	22.7257
C	10.8792	4.7780	7.1704	Cl	5.8371	24.9827	12.8426	Cl	25.4064	26.9041	3.7834	C	28.0090	13.4053	3.2193	C	16.4634	13.2860	11.7063
C	12.2130	4.0052	7.1068	H	6.5248	27.2433	13.1263	H	23.8772	28.1234	5.1560	Cl	27.1860	12.5380	4.5682	H	16.1097	12.6299	10.8805
C	13.2172	4.3372	5.9230	H	4.8770	27.1719	12.2603	H	23.5355	26.3281	5.2170	H	27.3915	14.2786	3.0613	Cl	16.0704	14.9502	11.1113
C	14.4982	3.4986	6.1459	Cl	2.9317	13.1048	14.0810	Cl	7.0382	0.5350	0.9501	H	29.0210	13.6624	3.4828	Cl	18.1988	13.1605	12.0486
C	12.5612	4.1789	4.5270	C	4.1552	12.7888	12.7727	C	7.8590	2.1301	0.6204	Cl	-1.3044	33.5996	-3.2524	H	16.0143	13.0463	12.6709
H	10.0974	4.4344	7.8749	Cl	3.5373	11.5841	11.6027	Cl	9.5613	2.1189	0.9354	C	-1.2499	33.7110	-5.0032	C	17.4643	20.1034	-2.4477
H	10.3263	4.7804	6.1876	H	5.0617	12.4783	13.2644	H	7.4487	2.8395	1.3320	Cl	-1.2385	35.3467	-5.7471	H	17.3744	19.5736	-1.5591
H	11.0990	5.8242	7.5359	H	4.3034	13.7252	12.1784	H	7.5705	2.3448	-0.4560	H	-2.1146	33.2373	-5.4604	Cl	19.2170	20.3421	-2.8154
H	11.9660	2.8902	7.0973	Cl	15.2291	11.1023	20.4919	Cl	10.1025	31.4124	3.8651	H	-0.2906	33.3254	-5.3056	Cl	16.4601	21.5817	-2.3694
H	12.7837	4.1485	7.9797	C	16.3786	10.9340	19.0768	C	9.1009	30.0458	4.4396	Cl	25.4835	25.2501	-0.0840	H	17.0706	19.4618	-3.2423
H	13.4426	5.3709	5.8789	Cl	17.9585	11.6417	19.4799	Cl	9.3647	28.5926	3.3863	C	24.5326	25.0600	-1.5488	C	13.5834	10.0120	14.1797
H	15.3108	3.9171	5.5587	H	16.0220	11.5484	18.2631	H	8.0568	30.3203	4.2916	Cl	24.3533	23.3207	-2.0384	H	14.5671	9.8688	13.7072
H	14.4199	2.3828	5.8636	H	16.5341	9.9089	18.8538	H	9.3623	29.8589	5.4993	H	25.0214	25.6188	-2.3562	Cl	12.4358	10.5485	12.9295
H	14.6952	3.5477	7.2006	Cl	7.9900	27.5301	-7.2216	Cl	7.1535	13.9059	21.4944	H	23.5318	25.5258	-1.2869	Cl	13.6464	11.2787	15.4781
H	11.7711	3.3964	4.5024	C	9.5260	28.0574	-7.8071	C	6.2743	15.4561	22.0383	Cl	-1.1610	13.5579	-9.9337	H	13.1497	9.1009	14.6557
H	13.2621	3.7531	3.7804	Cl	9.3357	28.8223	-9.3848												

Table S3. Cartesian coordinates (Å) of the structures in **Figure S20** corresponding to the reaction between iC$_4^+$ and 2 AlCl$_4^-$.

iC$_4^+$-AlCl$_4^-$ Reactant				iC$_4^+$ Transition State				iC$_4^=$ H$^+$(AlCl$_4^-$)$_2$ Product			
Al	9.3999	19.8783	4.2597	Al	11.6730	19.6603	4.1458	Al	12.4533	18.9942	4.7366
Cl	7.5526	19.9127	5.3983	Cl	9.5453	19.7162	3.8200	Cl	11.1252	20.0553	6.0162
Cl	11.0527	19.9804	5.6776	Cl	12.2324	21.0119	5.7251	Cl	14.3071	18.4268	5.5964
Cl	9.4843	21.5228	2.8832	Cl	12.7507	20.0605	2.3297	Cl	12.6341	19.8970	2.8069
Cl	9.5321	17.9768	3.2288	Cl	12.2113	17.6171	4.8006	Cl	11.3794	17.0306	4.1440
Al	10.2775	13.6322	9.3469	Al	10.6150	13.8815	8.3800	Al	11.5379	15.0810	8.3705
Cl	9.1969	12.4627	10.7551	Cl	11.9321	12.2269	8.5466	Cl	13.2076	13.7707	8.5695
Cl	10.3102	12.8600	7.3598	Cl	11.6915	14.7907	6.4278	Cl	10.8066	14.7865	6.2102
Cl	8.9587	15.5122	9.2397	Cl	8.6493	13.3922	7.7876	Cl	9.8547	14.4374	9.5282
C	8.7128	16.2501	6.4790	C	8.7699	16.8055	6.0188	C	8.0118	17.4767	5.5947
C	9.4970	16.8159	7.6244	C	8.7166	17.3517	7.2471	C	7.7536	17.2143	6.8848
C	9.0046	18.1136	8.1941	C	7.6803	16.9360	8.2594	C	6.9614	16.0081	7.3186
H	8.9469	16.8781	5.5958	H	9.4617	17.1729	5.2625	H	8.6090	18.3366	5.2992
H	7.6322	16.3042	6.6500	H	8.0827	16.0138	5.7289	H	7.6538	16.8234	4.8022
C	10.9876	16.6906	7.5124	C	9.6720	18.4227	7.6986	C	8.2555	18.0946	7.9987
Cl	12.1815	14.2947	10.0333	Cl	10.8363	15.3770	9.8552	Cl	11.9819	17.1426	8.5614
H	9.0144	15.2208	6.2514	H	11.1628	15.9914	6.1525	H	11.1749	16.0467	5.3250
H	11.2882	17.4464	6.7610	H	10.3516	18.7457	6.9006	H	8.8676	18.9227	7.6198
H	11.2969	15.7007	7.1573	H	10.2757	18.0543	8.5434	H	8.8701	17.5139	8.7047
H	11.4972	16.9235	8.4538	H	9.1279	19.3068	8.0640	H	7.4140	18.5078	8.5767
H	9.2101	18.8858	7.4283	H	7.0404	16.1281	7.8849	H	6.6185	15.4131	6.4628
H	9.5406	18.3868	9.1097	H	7.0436	17.7965	8.5185	H	6.0805	16.3073	7.9084
H	7.9235	18.1077	8.3715	H	8.1528	16.5975	9.1940	H	7.5727	15.3627	7.9705

Table S4. Cartesian coordinates (Å) of the structures in **Figure S20** corresponding to the reaction between iC$_5^+$ and 2 AlCl$_4^-$.

iC$_5^+$-AlCl$_4^-$ Reactant				iC$_5^+$ Transition State				iC$_5^=$ H$^+$(AlCl$_4^-$)$_2$ Product			
Al	8.6823	16.7453	5.9087	Al	11.6258	19.4773	4.0765	Al	11.0542	13.3840	7.2573
Cl	9.5930	15.2297	7.1631	Cl	9.5573	20.0794	4.0524	Cl	13.1318	13.0527	7.5838
Cl	8.8368	16.0788	3.8372	Cl	12.5140	19.9307	5.9889	Cl	10.6017	14.1484	5.3211
Cl	9.6568	18.6410	6.1556	Cl	12.7163	20.3735	2.4560	Cl	10.0891	14.4191	8.8554
Cl	6.5725	16.8805	6.3889	Cl	11.6987	17.2860	3.8281	Cl	10.0709	11.3100	7.3327
Al	3.5406	11.2342	2.9801	Al	10.7774	14.0521	8.2222	Al	9.8716	8.1924	4.4360
Cl	4.2002	12.0562	1.1209	Cl	12.0310	12.3397	8.2604	Cl	8.4953	7.9594	2.8173
Cl	5.4059	11.1425	4.3103	Cl	11.4851	14.7522	6.0237	Cl	10.4377	10.4059	4.3644
Cl	2.1525	12.4797	4.0041	Cl	8.7174	13.6095	8.0695	Cl	8.9817	7.9075	6.3533
C	6.5528	12.9926	4.2657	C	8.8268	17.0563	6.0805	C	6.7429	11.7349	4.6668
C	7.2892	12.8730	5.5710	C	8.5386	17.4596	7.3329	C	7.0113	12.7925	5.4552
C	6.4859	13.0526	6.8591	C	7.3412	16.9801	8.1102	C	6.7328	12.9487	6.9232
C	7.3954	12.7787	3.0411	H	9.6577	17.5587	5.5846	C	7.0923	11.7567	3.1979
C	5.5181	14.0652	4.1552	C	8.0629	16.0711	5.2348	C	6.0922	10.4591	5.1332
H	8.0528	13.6772	5.5063	C	9.3822	18.4958	8.0305	H	7.5035	13.6437	4.9853
Cl	2.9882	9.1821	2.8837	Cl	11.3338	15.6454	9.4828	Cl	11.6874	7.1152	4.1523
H	7.8634	11.9368	5.5746	H	11.0016	15.9752	5.7883	H	10.2392	10.8557	5.8690
H	5.6758	12.3164	6.9336	H	10.2499	18.8027	7.4301	H	6.3994	12.0186	7.3983
H	6.0602	14.0618	6.9247	H	9.7459	18.1125	8.9960	H	5.9559	13.7100	7.1015
H	7.1506	12.9312	7.7244	H	8.7879	19.3964	8.2492	H	7.6310	13.2927	7.4599
H	6.7823	12.6662	2.1395	H	6.7914	16.1819	7.5989	H	7.7381	10.9049	2.9332
H	8.0692	11.9212	3.1480	H	6.6461	17.8186	8.2779	H	7.6101	12.6829	2.9165
H	8.0081	13.6946	2.9398	H	7.6383	16.6096	9.1031	H	6.1877	11.6631	2.5760
H	5.0186	14.0560	3.1799	H	7.6373	15.2485	5.8239	H	5.1416	10.2935	4.6014
H	6.0682	15.0212	4.2643	H	8.7160	15.6306	4.4679	H	5.8846	10.4499	6.2092
H	4.7739	14.0222	4.9567	H	7.2390	16.5627	4.6941	H	6.7312	9.5910	4.9045

Table S5. Cartesian coordinates (Å) of the structures in **Figure S20** corresponding to the reaction between iC_6^+ and 2 $AlCl_4^-$.

iC_6^+-$AlCl_4^-$ Reactant				iC_6^+ Transition State				$iC_6^=$ $H^+(AlCl_4^-)_2$ Product			
Al	11.6033	14.4160	8.2141	Al	11.4107	19.3434	3.9017	Al	11.7802	13.8446	6.8131
Cl	13.3264	13.1254	7.9096	Cl	9.4486	20.2342	3.8942	Cl	13.8974	13.9258	6.5776
Cl	10.8533	14.9830	6.2578	Cl	12.3037	19.5056	5.8621	Cl	10.7115	14.2106	5.0024
Cl	12.1217	16.1344	9.3847	Cl	12.6656	20.2051	2.3870	Cl	11.0677	14.9669	8.4803
Cl	10.0612	13.2361	9.1910	Cl	11.1728	17.1907	3.5014	Cl	11.3088	11.6697	7.3613
Al	8.4303	9.2591	3.1027	Al	10.7131	14.0751	8.0806	Al	9.3399	8.5228	5.3217
Cl	8.9586	11.0177	2.0020	Cl	12.0713	12.4427	8.0872	Cl	8.3042	7.8928	3.5622
Cl	10.0539	9.0118	4.6682	Cl	11.6019	15.0149	6.0422	Cl	10.2334	10.5290	4.6580
Cl	6.5999	9.4797	4.1791	Cl	8.7097	13.5428	7.6828	Cl	8.0661	8.9538	6.9712
C	10.4763	10.9886	5.7585	C	8.7204	17.1858	6.1788	C	6.6200	11.8264	4.3042
C	10.9909	10.4149	7.0369	C	8.4611	17.4928	7.4654	C	6.9887	12.7146	5.2456
C	9.9919	9.7682	7.9988	C	7.3212	16.9116	8.2599	C	6.4660	12.8600	6.6488
C	11.4974	11.5674	4.8366	H	9.5174	17.7544	5.6951	C	7.2792	11.8219	2.9456
C	9.1465	11.6564	5.7655	C	7.9544	16.2613	5.2719	C	5.5681	10.7628	4.4858
H	11.4504	11.3007	7.5316	C	9.2888	18.5175	8.1989	H	7.7904	13.4080	4.9858
Cl	8.5619	7.4573	1.9745	Cl	11.0520	15.5409	9.5574	Cl	11.0045	7.2978	5.8335
H	11.8407	9.7507	6.8264	H	10.9181	16.1057	5.6940	H	10.7527	11.0975	5.9889
H	9.4837	8.9360	7.4928	H	10.1192	18.8929	7.5853	H	5.7063	12.0982	6.8657
H	9.2205	10.5024	8.2636	H	9.7043	18.0916	9.1243	H	5.9519	13.8314	6.7390
C	10.6904	9.2876	9.2781	H	8.6660	19.3758	8.4964	C	7.5929	12.7912	7.6993
H	11.0988	11.7025	3.8240	H	6.7508	16.1584	7.7050	H	7.7280	10.8383	2.7331
H	12.4232	10.9828	4.8156	H	6.6284	17.7135	8.5616	H	8.0685	12.5815	2.8737
H	11.7292	12.5724	5.2461	H	7.6904	16.4455	9.1864	H	6.5442	12.0076	2.1465
H	8.8470	11.9905	4.7658	H	7.4010	15.5117	5.8513	H	4.7471	10.9006	3.7644
H	9.2810	12.5551	6.4035	H	8.6654	15.6964	4.6499	H	5.1376	10.7509	5.4937
H	8.3593	11.0396	6.2105	C	7.0014	17.0410	4.3412	H	5.9980	9.7671	4.2908
H	11.4743	8.5500	9.0585	H	7.5535	17.7975	3.7656	H	8.3610	13.5549	7.5147
H	11.1608	10.1308	9.8028	H	6.4917	16.3725	3.6340	H	7.2081	12.9515	8.7156
H	9.9785	8.8178	9.9701	H	6.2354	17.5679	4.9250	H	8.0889	11.8126	7.6757

Supplementary Note 1. On the dominant ion-pair speciation

For the reactions detailed in **Figure 2** and **S1**, the amount of TBC (0.027−0.11 mmol) used to initiate disproportionation of isopentane is small compared to the chloroaluminate ionic liquid (1.0 mmol [$EMIM^+$][$Al_2Cl_7^-$]) such that $Al_2Cl_7^-$ remains the dominant anionic species during the reactions, as shown by Raman and ^{27}Al NMR analyses (**Figure S6D** and **S7F**). The active species are envisioned to be *t*-Alk^+-$AlCl_4^-$ ion-pairs, which all originate from the initially formed *i*C_4^+-$AlCl_4^-$ ion-pairs by the reaction between $Al_2Cl_7^-$ and TBC. However, equilibration of *t*-Alk^+-$AlCl_4^-$ ion-pairs with excess $Al_2Cl_7^-$ could also yield *t*-Alk^+-$Al_2Cl_7^-$ ion-pairs via the anion exchange reaction.[33]

To test whether this reaction may be favorable, we performed DFT calculations on the prototype reaction: *i*C_4^+-$AlCl_4^-$ + $Al_2Cl_7^-$ ⇌ $AlCl_4^-$ + *i*C_4^+-$Al_2Cl_7^-$. **Table S6** lists the enthalpy and free energy changes obtained at the B3LYP/aug-cc-pvdz level for association reactions of *i*C_4^+ with $AlCl_4^-$ and $Al_2Cl_7^-$ with calculations of solution phase energetics, $\Delta H_{(DCM)}$ and $\Delta G_{(DCM)}$, also employing the COSMO solvation model to incorporate implicit dichloromethane solvation. Note that the difference between these two association reactions equates to the above exchange reaction and, as such, the results reveal that the *i*C_4^+-$AlCl_4^-$ ion-pair is far more favorable than the *i*C_4^+-$Al_2Cl_7^-$ ion-pair, both in gas and in solution phases. Notably, the free energy change for the anion exchange in DCM solution is 97 kJ/mol. Consequently, the anion exchange reaction to form *i*C_4^+-$Al_2Cl_7^-$ from *i*C_4^+-$AlCl_4^-$ is highly endergonic, rendering its concentration negligible. Therefore, we conclude that *i*C_4^+-$AlCl_4^-$ ion-pairs and, by analogy, *t*-Alk^+-$AlCl_4^-$ ion-pairs constitute the MARI in this system.

Table S6. Thermodynamics for the interaction of *i*C_4^+ with $AlCl_4^-$ or $Al_2Cl_7^-$. ΔH and ΔG (kJ/mol) are calculated using the harmonic oscillator approximation at 298 K and 1 atm. $\Delta H_{(gas)}$ and $\Delta G_{(gas)}$ refer to gas-phase calculation of the isolated complex while $\Delta H_{(DCM)}$ and $\Delta G_{(DCM)}$ employ the COSMO method to include implicit solvation effects of a dichloromethane (DCM) solvent.

Reaction	$\Delta H_{(gas)}$	$\Delta G_{(gas)}$	$\Delta H_{(DCM)}$	$\Delta G_{(DCM)}$
iC_4^+ + $AlCl_4^-$ ⇌ *i*C_4^+-$AlCl_4^-$	-359.10	-302.69	-79.57	-17.95
iC_4^+ + $Al_2Cl_7^-$ ⇌ *i*C_4^+-$Al_2Cl_7^-$	-295.95	-232.02	18.64	78.98

Table S7. Cartesian coordinates (Å) for the structures optimized at B3LYP/aug-cc-pVDZ(C, Al, Cl) and cc-pVDZ(H) employing the COSMO implicit solvent model for dichloromethane (ε= 9.08).

$Al_2Cl_7^-$			
Cl	0.8089	-0.0170	-0.8551
Al	-0.0094	-2.0407	0.0052
Cl	1.8141	-3.1791	0.2730
Cl	-1.2476	-2.8177	-1.5888
Al	0.0322	2.0348	-0.0290
Cl	0.9387	3.3805	-1.4687
Cl	-2.1198	1.9975	-0.1342
Cl	-1.0616	-1.6161	1.8323
Cl	0.8529	2.2514	1.9538

$AlCl_4^-$–iC_4^+			
C	-0.2074	-2.6746	0.1262
C	0.5793	-2.8374	1.4139
C	-1.3703	-3.6438	-0.0075
C	0.6551	-2.5896	-1.1181
H	-0.0781	-2.8312	2.2920
H	1.3399	-2.0562	1.5232
H	1.0882	-3.8137	1.3710
H	-2.0303	-3.6108	0.8683
H	-0.9504	-4.6592	-0.0814
H	-1.9584	-3.4480	-0.9124
H	1.4624	-1.8567	-1.0097
H	0.0641	-2.3480	-2.0090
H	1.1131	-3.5808	-1.2654
Al	0.2169	1.0219	-0.3061
Cl	-1.0689	2.4742	0.5882
Cl	2.0988	0.6390	0.6571
Cl	0.2535	0.9289	-2.4457
Cl	-1.1219	-0.9321	0.3070

iC_4^+			
C	-0.0005	-0.0009	-0.0025
C	1.2498	0.7619	-0.0035
C	-1.2846	0.7019	0.0061
C	0.0342	-1.4648	-0.0033
H	1.2056	1.5463	-0.7813
H	2.1540	0.1533	-0.1005
H	1.2796	1.3412	0.9423
H	-1.2085	1.7852	0.1425
H	-1.9743	0.2405	0.7336
H	-1.7601	0.4867	-0.9755
H	0.7023	-1.8209	-0.8082
H	-0.9491	-1.9423	-0.0554
H	0.5585	-1.7793	0.9232

$AlCl_4^-$			
Al	0.1406	-0.0119	-0.0262
Cl	-1.9727	-0.0179	-0.8405
Cl	0.1456	-1.7281	1.5877
Cl	0.3182	1.8377	1.2677
Cl	1.3504	-0.0768	-1.9691

$Al_2Cl_7^-$–iC_4^+			
C	-0.2325	4.7900	-0.2345
C	1.0847	4.6773	-0.9616
C	-1.1832	5.8207	-0.7980
C	-0.1379	4.7693	1.2707
H	0.9458	4.5983	-2.0462
H	1.6817	3.8349	-0.5957
H	1.6365	5.6083	-0.7511
H	-1.2998	5.7180	-1.8836
H	-0.7340	6.8057	-0.5923
H	-2.1640	5.7849	-0.3094
H	0.4697	3.9319	1.6305
H	-1.1274	4.7420	1.7414
H	0.3578	5.7087	1.5648
Al	0.0279	1.1463	0.0798
Cl	-0.7021	-1.0713	-1.1068
Cl	2.2412	0.9273	0.0010
Cl	-0.7976	1.1496	2.1078
Cl	-1.2273	3.0465	-0.7068
Al	0.0103	-3.5069	-0.3384
Cl	-1.1393	-5.3071	-1.1340
Cl	-0.8170	-3.5041	1.7058
Cl	2.2382	-3.0785	-0.4878

Table S8. Cartesian coordinates (Å) for the structures optimized at B3LYP/aug-cc-pVDZ(C, Al, Cl) and cc-pVDZ(H).

$Al_2Cl_7^-$			
Cl	0.8089	-0.0170	-0.8551
Al	-0.0094	-2.0407	0.0052
Cl	1.8141	-3.1791	0.2730
Cl	-1.2476	-2.8177	-1.5888
Al	0.0322	2.0348	-0.0290
Cl	0.9387	3.3805	-1.4687
Cl	-2.1198	1.9975	-0.1342
Cl	-1.0616	-1.6161	1.8323
Cl	0.8529	2.2514	1.9538

$AlCl_4^-$–*i*C_4^+			
C	-0.2074	-2.6746	0.1262
C	0.5793	-2.8374	1.4139
C	-1.3703	-3.6438	-0.0075
C	0.6551	-2.5896	-1.1181
H	-0.0781	-2.8312	2.2920
H	1.3399	-2.0562	1.5232
H	1.0882	-3.8137	1.3710
H	-2.0303	-3.6108	0.8683
H	-0.9504	-4.6592	-0.0814
H	-1.9584	-3.4480	-0.9124
H	1.4624	-1.8567	-1.0097
H	0.0641	-2.3480	-2.0090
H	1.1131	-3.5808	-1.2654
Al	0.2169	1.0219	-0.3061
Cl	-1.0689	2.4742	0.5882
Cl	2.0988	0.6390	0.6571
Cl	0.2535	0.9289	-2.4457
Cl	-1.1219	-0.9321	0.3070

iC_4^+			
C	-0.0005	-0.0009	-0.0025
C	1.2498	0.7619	-0.0035
C	-1.2846	0.7019	0.0061
C	0.0342	-1.4648	-0.0033
H	1.2056	1.5463	-0.7813
H	2.1540	0.1533	-0.1005
H	1.2796	1.3412	0.9423
H	-1.2085	1.7852	0.1425
H	-1.9743	0.2405	0.7336
H	-1.7601	0.4867	-0.9755
H	0.7023	-1.8209	-0.8082
H	-0.9491	-1.9423	-0.0554
H	0.5585	-1.7793	0.9232

$AlCl_4^-$			
Al	0.0000	-0.0002	-0.0001
Cl	-0.0321	-2.1877	-0.2140
Cl	1.8211	0.6043	1.0724
Cl	-1.7678	0.6512	1.1324
Cl	-0.0211	0.9313	-1.9908

$Al_2Cl_7^-$–*i*C_4^+			
C	-0.2325	4.7900	-0.2345
C	1.0847	4.6773	-0.9616
C	-1.1832	5.8207	-0.7980
C	-0.1379	4.7693	1.2707
H	0.9458	4.5983	-2.0462
H	1.6817	3.8349	-0.5957
H	1.6365	5.6083	-0.7511
H	-1.2998	5.7180	-1.8836
H	-0.7340	6.8057	-0.5923
H	-2.1640	5.7849	-0.3094
H	0.4697	3.9319	1.6305
H	-1.1274	4.7420	1.7414
H	0.3578	5.7087	1.5648
Al	0.0279	1.1463	0.0798
Cl	-0.7021	-1.0713	-1.1068
Cl	2.2412	0.9273	0.0010
Cl	-0.7976	1.1496	2.1078
Cl	-1.2273	3.0465	-0.7068
Al	0.0103	-3.5069	-0.3384
Cl	-1.1393	-5.3071	-1.1340
Cl	-0.8170	-3.5041	1.7058
Cl	2.2382	-3.0785	-0.4878

Supplementary Note 2. Derivation of rate equation based on the core kinetic model

In our core kinetic model (**Figure 5A**), the isopentane disproportionation mechanism is greatly simplified and can be described with the following three elementary steps:

$TBC \overset{k_{Ini}}{\rightarrow} C^{+}$ (Step 1, Initiation via chloride abstraction)

$C^{+} \overset{K_{DP}}{\leftrightarrow} C^{=} + HCl$ (Step 2, Deprotonation equilibrium)

$iC_5 + C^{+} \overset{k_{HT}}{\rightarrow} P + C^{+}$ (Step 3, Hydride transfer)

k_{Ini} and k_{HT} are the rate constants of the initiation via chloride abstraction and hydride transfer, respectively.

K_{DP} is the equilibrium constant of the deprotonation/protonation equilibrium.

In the subsequent sections, the rate equations for the transient and steady-state regimes are derived separately using rational assumptions based on the characteristics of each regime.

1. Transient regime

In the transient regime, the product is formed solely by the hydride transfer step. Thus, the overall reaction rate can be expressed as:

$$r_{\text{transient regime}} = r_{HT} = k_{HT}[C^{+}][iC_5] \quad \text{(Eq. 1)}$$

Our control experiments (**Figure S2B**) show that the isopentane concentration has little impact on the reaction rate. Thus, the rate expression can be simplified to:

$$r_{\text{transient regime}} = r_{HT} = k_{HT}[C^{+}] \quad \text{(Eq. 2)}$$

Initially, the carbenium ions are introduced into the system via chloride abstraction. Our simulation shows that the initiator TBC readily transfers its chloride to an $AlCl_3$*, forming an iC_4^{+}-$AlCl_4^{-}$ ion-pair. It is thus reasonable to assume that the initiation via chloride abstraction is much faster than other elementary steps and also irreversible. Therefore, the initial carbenium ion concentration can be expressed as:

$[C^{+}]_0 = [TBC]_0$ (Eq. 3)

Next, the initial carbenium ions are involved in three elementary steps: deprotonation, protonation, and hydride transfer. In this core model, the simplification is that the carbenium ion only functions as a catalytic chain carrier and is not consumed by the hydride transfer step. Thus, the carbenium ion concentration is only controlled by the deprotonation and protonation steps. The change in carbenium ion concentration can then be expressed as:

$$\frac{d[C^{+}]}{dt} = -k_{DP}[C^{+}] + k_{-DP}[C^{=}][HCl] \quad \text{(Eq. 4)}$$

During the transient regime, the system can be considered as carbenium ion-rich. Therefore, $[C^{=}]$ and [HCl] are negligible. Equation 4 can be simplified to:

$$\frac{d[C^{+}]}{dt} = -k_{DP}[C^{+}] \quad \text{(Eq. 5)}$$

Solve the first-order differential equation (Eq. 5), and the time-dependent carbenium ion concentration can be expressed as:

$$[C^{+}]_t = [C^{+}]_0 \cdot e^{-k_{DP} \cdot t} \quad \text{(Eq. 6)}$$

Insert (Eq. 3) into (Eq. 6):

$$[C^{+}]_t = [TBC]_0 \cdot e^{-k_{DP} \cdot t} \quad \text{(Eq. 7)}$$

The time-dependent reaction rate in the transient regime can be obtained by inserting (Eq. 7) into (Eq. 2):

$$r(t)_{\text{transient regime}} = k_{HT} \cdot [C^{+}]_t = k_{HT} \cdot [TBC]_0 \cdot e^{-k_{DP} \cdot t} \quad \text{(Eq. 8)}$$

The total product formation in the transient regime can be obtained by integrating the time-dependent reaction rate $r(t)$ over the time interval from t = 0 to t

$$\text{Yield}_{\text{transient regime}} = \int_0^t r(t)_{\text{transient regime}} dt = \frac{k_{HT} \cdot [TBC]_0}{k_{DP}} (1 - e^{-k_{DP} \cdot t}) \quad \text{(Eq. 9)}$$

According to this equation, the product formation achieved in the transient regime is determined by three parameters: the initial TBC concentration $[TBC]_0$, the hydride transfer rate constant k_{HT}, and the deprotonation rate constant k_{DP}. The term $[TBC]_0$ linearly scales the maximum product yield achievable in the transient regime, as illustrated in **Figure S1**. Similarly, the term k_{HT} is also proportional to the total product formation in the transient regime. An increase in k_{HT} enhances the rate at which the product is formed, leading to a greater yield during the transient regime. Conversely, a decrease in k_{HT} reduces the yield under the same conditions. The effect of k_{DP} on the transient regime is multifaceted. In the denominator, k_{DP} inversely scales the yield. A

higher k_{DP} reduces the constant pre-factor, lowering the yield of the transient regime. In the exponential term, a larger k_{DP} causes the term $(1 - e^{-k_{DP} \cdot t})$ to approach 1 more rapidly. This means that the product yield reaches its maximum value faster, i.e., the transient regime ends faster. The influence of k_{HT} and k_{DP} on the transient regime agrees well with what we described in **Figure S15C**, **S15D**, and **S15E**.

2. Steady-state regime

In the steady-state regime, the deprotonation equilibrium between $C^{=}$ and C^{+} is established. Then we have

$$K_{DP} = \frac{[C^{=}][HCl]}{[C^{+}]} \qquad \text{(Eq. 10)}$$

, where $K_{DP} = k_{DP}/k_{-DP}$

As all carbenium ions and alkenes in the system result from the TBC addition, we have

$$[C^{+}] + [C^{=}] = [TBC]_0 \qquad \text{(Eq. 11)}$$

Furthermore, since the deprotonation step produces both alkenes and HCl in equimolar amounts, we can conclude that:

$$[C^{=}] = [HCl] \qquad \text{(Eq. 12)}$$

Insert (Eq. 11) and (Eq. 12) into (Eq. 10):

$$[C^{+}] = \frac{2[TBC]_0 + K_{DP} \pm \sqrt{K_{DP}^2 + 4[TBC]_0 \cdot K_{DP}}}{2} \qquad \text{(Eq. 13)}$$

Because all carbenium ions are formed from TBC, $[C^{+}]$ must not exceed $[TBC]_0$, the negative root is chosen:

$$[C^{+}] = \frac{2[TBC]_0 + K_{DP} - \sqrt{K_{DP}^2 + 4[TBC]_0 \cdot K_{DP}}}{2} \qquad \text{(Eq. 14)}$$

Factor K_{DP}^2 from the square root:

$$[C^{+}] = \frac{2[TBC]_0 + K_{DP} - K_{DP}\sqrt{1 + \frac{4[TBC]_0}{K_{DP}}}}{2} \qquad \text{(Eq. 15)}$$

The deprotonation equilibrium heavily favors the alkene formation, K_{DP}>>1; additionally, TBC is added in very small quantities, $[TBC]_0$<<1. Thus, $\frac{4[TBC]_0}{K_{DP}}$<<1. In this case, the second-order Taylor approximation can be applied to the square root:

$$\sqrt{1 + \frac{4[TBC]_0}{K_{DP}}} \approx 1 + \frac{2[TBC]_0}{K_{DP}} - \frac{2[TBC]_0^2}{K_{DP}^2} \qquad \text{(Eq. 16)}$$

Insert (Eq. 16) into (Eq.15), the carbenium concentration can be simplified to:

$$[C^+] = \frac{[TBC]_0{}^2}{K_{DP}} \qquad \text{(Eq. 17)}$$

Like the transient regime, the product is formed solely by the hydride transfer step. Thus, the overall reaction rate can be expressed as

$$r_{\text{steady-state regime}} = r_{HT} = k_{HT}[C^+] \qquad \text{(Eq. 18)}$$

Insert (Eq. 17) into (Eq. 18):

$$r_{\text{steady-state regime}} = \frac{k_{HT} \cdot [TBC]_0{}^2}{K_{DP}} = \frac{k_{HT} \cdot k_{-DP} \cdot [TBC]_0{}^2}{k_{DP}} \qquad \text{(Eq. 19)}$$

According to this rate equation, the reaction rate in the steady-state regime is determined by four parameters: the initial TBC concentration $[TBC]_0$, the hydride transfer rate constant k_{HT}, the deprotonation rate constant k_{DP}, and the protonation rate constant k_{-DP}. The steady-state regime reaction rate exhibits a second-order dependence on the initial TBC concentration. Among the rate constants, k_{HT} and k_{-DP} are positively correlated with the reaction rate, while k_{DP} inversely affects it. Compared to the transient regime, the steady-state regime is additionally influenced by k_{-DP}. These conclusions are in good alignment with the sensitivity analysis for the core model (**Figure S15E** and **S15F**).

References

(1) Zhang, W.; Kim, S.; Wahl, L.; Khare, R.; Hale, L.; Hu, J.; Camaioni, D. M.; Gutiérrez, O. Y.; Liu, Y.; Lercher, J. A. Low-temperature upcycling of polyolefins into liquid alkanes via tandem cracking-alkylation. *Science* **2023**, *379* (6634), 807-811. DOI: 10.1126/science.ade7485.

(2) VandeVondele, J.; Krack, M.; Mohamed, F.; Parrinello, M.; Chassaing, T.; Hutter, J. QUICKSTEP: Fast and accurate density functional calculations using a mixed Gaussian and plane waves approach. *Comput. Phys. Commun.* **2005**, *167* (2), 103-128. DOI: 10.1016/j.cpc.2004.12.014.

(3) Perdew, J. P.; Burke, K.; Ernzerhof, M. Generalized Gradient Approximation Made Simple. *Phys. Rev. Lett.* **1996**, *77* (18), 3865-3868. DOI: 10.1103/PhysRevLett.77.3865.

(4) Grimme, S.; Antony, J.; Ehrlich, S.; Krieg, H. A consistent and accurate ab initio parametrization of density functional dispersion correction (DFT-D) for the 94 elements H-Pu. *J. Chem. Phys.* **2010**, *132* (15). DOI: 10.1063/1.3382344.

(5) Goedecker, S.; Teter, M.; Hutter, J. Separable dual-space Gaussian pseudopotentials. *Phys. Rev. B* **1996**, *54* (3), 1703-1710. DOI: 10.1103/PhysRevB.54.1703.

(6) Nose, S. A unified formulation of the constant temperature molecular dynamics methods *J. Chem. Phys.* **1984**, *81* (1), 511-519. DOI: 10.1063/1.447334.

(7) Carter, E. A.; Ciccotti, G.; Hynes, J. T.; Kapral, R. Constrained reaction coordinate dynamics for the simulation of rare events. *Chem. Phys. Lett.* **1989**, *156* (5), 472-477. DOI: 10.1016/S0009-2614(89)87314-2.

(8) Henkelman, G.; Jónsson, H. Improved tangent estimate in the nudged elastic band method for finding minimum energy paths and saddle points. *J. Chem. Phys.* **2000**, *113* (22), 9978-9985. DOI: 10.1063/1.1323224.

(9) Heyden, A.; Bell, A. T.; Keil, F. J. Efficient methods for finding transition states in chemical reactions: Comparison of improved dimer method and partitioned rational function optimization method. *J. Chem. Phys.* **2005**, *123* (22). DOI: 10.1063/1.2104507.

(10) Valiev, M.; Bylaska, E. J.; Govind, N.; Kowalski, K.; Straatsma, T. P.; Van Dam, H. J. J.; Wang, D.; Nieplocha, J.; Apra, E.; Windus, T. L.; et al. NWChem: A comprehensive and scalable open-source solution for large scale molecular simulations. *Comput. Phys. Commun.* **2010**, *181* (9), 1477-1489. DOI: 10.1016/j.cpc.2010.04.018.

(11) Becke, A. D. Density-functional thermochemistry. III. The role of exact exchange. *J. Chem. Phys.* **1993**, *98* (7), 5648-5652. DOI: 10.1063/1.464913 (acccessed 2/4/2026).

(12) Lee, C.; Yang, W.; Parr, R. G. Development of the Colle-Salvetti correlation-energy formula into a functional of the electron density. *Phys. Rev. B* **1988**, *37* (2), 785-789. DOI: 10.1103/PhysRevB.37.785.

(13) Kendall, R. A.; Dunning, T. H., Jr.; Harrison, R. J. Electron affinities of the first-row atoms revisited. Systematic basis sets and wave functions. *J. Chem. Phys.* **1992**, *96* (9), 6796-6806. DOI: 10.1063/1.462569 (acccessed 2/4/2026).

(14) Klamt, A.; Schüürmann, G. COSMO: a new approach to dielectric screening in solvents with explicit expressions for the screening energy and its gradient. *J. Chem. Soc., Perkin Trans. 2* **1993**, (5), 799-805, 10.1039/P29930000799. DOI: 10.1039/P29930000799.

(15) York, D. M.; Karplus, M. A Smooth Solvation Potential Based on the Conductor-Like Screening Model. *J. Phys. Chem. A* **1999**, *103* (50), 11060-11079. DOI: 10.1021/jp992097l.

(16) Zhang, W.; Yao, H.; Khare, R.; Zhang, P. R.; Yang, B. D.; Hu, W. D.; Ray, D.; Hu, J. Z.; Camaioni, D. M.; Wang, H. M.; et al. Chloride and Hydride Transfer as Keys to Catalytic Upcycling of Polyethylene into Liquid Alkanes. *Angew. Chem., Int. Ed.* **2024**, *63* (17). DOI: 10.1002/anie.202319580.

(17) Weitkamp, J. Catalytic Hydrocracking—Mechanisms and Versatility of the Process. *ChemCatChem* **2012**, *4* (3), 292-306. DOI: 10.1002/cctc.201100315.

(18) Angell, M.; Zhu, G. Z.; Lin, M. C.; Rong, Y. M.; Dai, H. J. Ionic Liquid Analogs of $AlCl_3$ with Urea Derivatives as Electrolytes for Aluminum Batteries. *Adv. Funct. Mater.* **2020**, *30* (4). DOI: 10.1002/adfm.201901928.

(19) Estager, J.; Holbrey, J. D.; Swadźba-Kwaśny, M. Halometallate ionic liquids – revisited. *Chem. Soc. Rev.* **2014**, *43* (3), 847-886. DOI: 10.1039/c3cs60310e.

(20) Gilbert, B.; Olivier-Bourbigou, H.; Favre, F. Chloroaluminate Ionic Liquids: from their Structural Properties to their Applications in Process Intensification. *Oil Gas Sci. Technol.* **2007**, *62* (6), 745-759. DOI: 10.2516/ogst:2007068.

(21) Ng, K. L.; Dong, T.; Anawati, J.; Azimi, G. High-Performance Aluminum Ion Battery Using Cost-Effective $AlCl_3$-Trimethylamine Hydrochloride Ionic Liquid Electrolyte. *Adv. Sustainable Syst.* **2020**, *4* (8). DOI: 10.1002/adsu.202000074.

(22) Hacura, A.; Zerda, T. W.; Kaczmarski, M. Temperature and pressure Raman study of molecular motions in liquid CH_2Cl_2. *J. Raman Spectrosc.* **1981**, *11* (6), 437-441. DOI: 10.1002/jrs.1250110604.

(23) Edwards, H. G. M.; Farwell, D. W.; Johnson, A. F. FT-Raman spectroscopic study of aluminium(III) chloride in acetonitrile and dichloromethane solutions containing water. *J. Mol. Struct.* **1995**, *344* (1-2), 37-44. DOI: 10.1016/0022-2860(94)08413-C.

(24) Gale, R. J.; Gilbert, B.; Osteryoung, R. A. Raman spectra of molten aluminum chloride: 1-butylpyridinium chloride systems at ambient temperatures. *Inorg. Chem.* **1978**, *17* (10), 2728-2729. DOI: 10.1021/ic50188a008.

(25) Jach, F.; Wassner, M.; Bamberg, M.; Brendler, E.; Frisch, G.; Wunderwald, U.; Friedrich, J. A Low-Cost Al-Graphite Battery with Urea and Acetamide-Based Electrolytes. *ChemElectroChem* **2021**, *8* (11), 1928-1929. DOI: 10.1002/celc.202100544.

(26) Eiden, P.; Liu, Q. X.; El Abedin, S. Z.; Endres, F.; Krossing, I. An Experimental and Theoretical Study of the Aluminium Species Present in Mixtures of $AlCl_3$ with the Ionic Liquids [BMP]Tf_2N and [EMIm]Tf_2N. *Chem. - Eur. J.* **2009**, *15* (14), 3426-3434. DOI: 10.1002/chem.200801616.

(27) Ferrara, C.; Dall'Asta, V.; Berbenni, V.; Quartarone, E.; Mustarelli, P. Physicochemical Characterization of $AlCl_3$–1-Ethyl-3-methylimidazolium Chloride Ionic Liquid Electrolytes for Aluminum Rechargeable Batteries. *J. Phys. Chem. C* **2017**, *121* (48), 26607-26614. DOI: 10.1021/acs.jpcc.7b07562.

(28) Zhang, J.; Huang, C. P.; Chen, B. H.; Ren, P. J.; Pu, M. Isobutane/2-butene alkylation catalyzed by chloroaluminate ionic liquids in the presence of aromatic additives. *J. Catal.* **2007**, *249* (2), 261-268. DOI: 10.1016/j.jcat.2007.04.019.

(29) Zhang, W.; Khare, R.; Kim, S.; Hale, L.; Hu, W.; Yuan, C.; Sheng, Y.; Zhang, P.; Wahl, L.; Mai, J.; et al. Active species in chloroaluminate ionic liquids catalyzing low-temperature polyolefin deconstruction. *Nat. Commun.* **2024**, *15* (1), 5785. DOI: 10.1038/s41467-024-49827-4.

(30) Amarasekara, A. S. Acidic Ionic Liquids. *Chem. Rev.* **2016**, *116* (10), 6133-6183. DOI: 10.1021/acs.chemrev.5b00763.

(31) Schaber, J. Easy parameter identifiability analysis with COPASI. *BioSystems* **2012**, *110* (3), 183-185. DOI: 10.1016/j.biosystems.2012.09.003.

(32) Ryan, D. E.; Fuller, J. T., III; Patrick, E. A.; Erickson, J. D.; Speelman, A. L.; Carroll, T. G.; Schenter, G. K.; Ginovska, B.; Raugei, S.; Bullock, R. M.; et al. Mechanistic Insights into Molecular Copper Hydride Catalysis: the Kinetic Stability of CuH Monomers toward Aggregation is a Critical Parameter for Catalyst Performance. *J. Am. Chem. Soc.* **2025**, *147* (17), 14280-14298. DOI: 10.1021/jacs.4c17955.

(33) Shilina, M. I.; Bakharev, R. V.; Petukhova, A. V.; Smirnov, V. V. Generation and IR spectra of ionic and molecular complexes of aluminum chloride with tert- and sec-butyl chlorides. *Russ. Chem. Bull.* **2005**, *54* (1), 149-158. DOI: 10.1007/s11172-005-0231-6.
